# Discovery of a Potent and Highly Selective Dipeptidyl Peptidase IV and Carbonic Anhydrase Inhibitor as “Antidiabesity” Agents Based on Repurposing and Morphing of WB-4101

**DOI:** 10.1021/acs.jmedchem.2c01192

**Published:** 2022-10-06

**Authors:** Angelica Artasensi, Andrea Angeli, Carmen Lammi, Carlotta Bollati, Silvia Gervasoni, Giovanna Baron, Rosanna Matucci, Claudiu T. Supuran, Giulio Vistoli, Laura Fumagalli

**Affiliations:** † Department of Pharmaceutical Sciences “DISFARM”, Università degli Studi di Milano, via Mangiagalli 25, I-20133 Milan, Italy; ‡ Department of Pharmaceutical Sciences “NEUROFARBA”, University of Florence, via Ugo Schiff 6, 50019 Sesto Fiorentino, Florence, Italy; § Department of Physics, Citt. Universitaria, University of Cagliari, I-09042 Cagliari, Monserrato, Italy; ∥ Department of Pharmacology and Toxicology “NEUROFARBA”, University of Florence, Viale Pieraccini 6, 50134 Florence, Italy

## Abstract

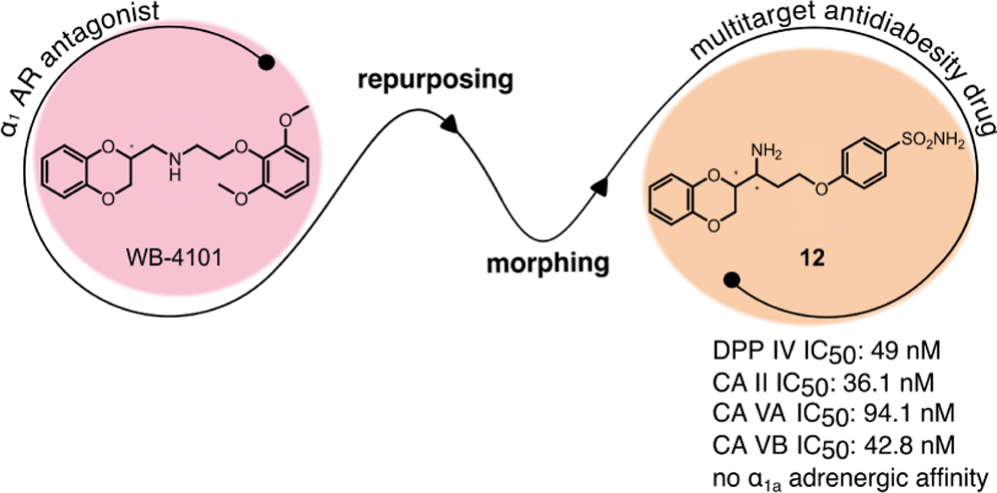

The management of patients with type 2 diabetes mellitus (T2DM) is shifting from cardio-centric to weight-centric or, even better, adipose-centric treatments. Considering the downsides of multidrug therapies and the relevance of dipeptidyl peptidase IV (DPP IV) and carbonic anhydrases (CAs II and V) in T2DM and in the weight loss, we report a new class of multitarget ligands targeting the mentioned enzymes. We started from the known α_1_-AR inhibitor WB-4101, which was progressively modified through a tailored morphing strategy to optimize the potency of DPP IV and CAs while losing the adrenergic activity. The obtained compound **12** shows a satisfactory DPP IV inhibition with a good selectivity CA profile (DPP IV IC_50_: 0.0490 μM; CA II *K_i_* 0.2615 μM; CA VA *K_i_* 0.0941 μM; CA VB *K_i_* 0.0428 μM). Furthermore, its DPP IV inhibitory activity in Caco-2 and its acceptable pre-ADME/Tox profile indicate it as a lead compound in this novel class of multitarget ligands.

## Introduction

Type 2 diabetes mellitus (T2DM) is a chronic condition characterized by the dysregulation of carbohydrate, lipid, and protein metabolism and results from impaired insulin secretion, insulin resistance, or a combination of both. Of the three major types of diabetes, T2DM is far more common (accounting for more than 90% of all cases) than either type 1 diabetes mellitus (T1DM) or gestational diabetes.^1^

Globally, 6.28% of the world’s population is affected by T2DM. Developed regions such as Western Europe show higher prevalence rates that continue to increase despite public health measures. The distribution of T2DM generally matches the socioeconomic development even though the burden of suffering due to diabetes is rapidly increasing in lower-income countries.^2^

Thus, T2DM is recognized as a global public health concern, which directly impacts human life and health expenditures. With regard to the cost of diabetes care, it is 3.2 times greater than the average per capita healthcare expenditure, even reaching 9.4 times in the presence of comorbidities. As a matter of fact, over time, diabetes can damage the heart, blood vessels, eyes, kidneys, and nerves. The damages occur more frequently in patients affected by T2DM since symptoms are less marked than those revealed to T1DM; thus, the disease may be diagnosed several years after the onset. Moreover, T2DM incidence has increased in lockstep with the obesity pandemic during the last half-century.^3^ In fact, the numbers of individuals with T2DM parallel the numbers of adults, with obesity generating a worldwide dual epidemic, which is an important public health issue.^4^ The detrimental health effects of diabetes and obesity are well known so much that they are described by the term “diabesity”.

For these reasons and considering that the two conditions share key pathophysiological mechanisms, the management of patients with T2DM is shifting from a cardio-centric goal to a new weight-centric, or, even better, adipose-centric treatment goal.^5^ Since diabetes is characterized by a complex physiopathology, pharmacological monotherapies have proven ineffective in controlling blood glucose levels and other comorbidities. Therefore, therapeutic treatment is frequently done through drug combinations, which operate with different mechanisms of action^6^ but with potential drug–drug interaction risks. Moreover, a complex combinatorial regimen is one of the leading causes of nonadherence to therapeutic recommendations^7^ and this represents a serious concern since compliance is a *conditio sine qua non* for improving outcomes for patients with diabetes.

In a state of metabolic disturbance, like the one affecting both diabetic and obese patients, multitarget drugs that concomitantly normalize glycemia and inhibit the progression of comorbidities could be a useful option for the management of these conditions. Given the relevance of dipeptidyl peptidase IV (DPP IV) and carbonic anhydrase isoforms II and V (CAs II and V) roles in the pathology of T2DM as well as in the weight loss, multitarget ligands able to modulate these enzymes could represent a promising therapeutic approach for antidiabesity treatment. DPP IV plays an important role in maintaining glucose homeostasis since it is responsible for the inactivation of the incretin hormones, namely, the glucose-dependent insulinotropic polypeptide hormone (GIP) and the glucagon-like peptide 1 (GLP-1).^8^ These endocrine hormones are released from the gut in response to intraluminal carbohydrates, and they are implicated in numerous desirable pancreatic actions, including insulin secretion and gene expression stimulation, increasing β-cell survival, enhancing β-cell glucose sensitivity, and reducing glucagon production.^9,10^

Human carbonic anhydrases (hCAs) are ubiquitous zinc-metalloenzymes, which act as efficient catalysts for the reversible hydration of carbon dioxide to bicarbonate and protons. They are involved in many conditions either physiological or pathological such as electrolyte secretion and biosynthetic reactions like gluconeogenesis, lipogenesis, and ureagenesis. In particular, isoforms II and V play a significant role in metabolic processes such as gluconeogenesis.^11^ CA II is the most physiologically relevant isoform and it has been found in several organs/tissues. Several studies demonstrated that CA II interacts with a variety of membrane-bound carriers to regulate the cytoplasmic pH, such as the sodium/hydrogen exchanger (NHE1)^12^ or the sodium bicarbonate cotransporter (NBC1).^13^ Recent investigation shows that NHE1/CA II metabolon complex is exacerbated in diabetic cardiomyopathy of ob^–/–^ mice, which may lead to perturbation of intracellular pH and Na^+^ and Ca^2+^ concentrations, contributing to cardiovascular anomalies. Moreover, the enhanced NHE1/CA II metabolon activity was correlated with an increased CA II expression in hypertrophic and functionally impaired ob^–/–^ mice hearts.^14^ Evidence proves also that CA II is overexpressed in diabetic ischemic human myocardium.

CA V is, among all of the isoforms, the only one located in the mitochondria. There are two mitochondrial CAs with different tissue distributions and they are usually referred to as CA VA and CA VB. These isoforms influence many physiological processes, like CO_2_ transport, bone resorption, gluconeogenesis, production of body fluids, lipogenesis, ureagenesis, and de novo synthesis of HCO_3_^–^ within the mitochondrial compartment.^15^ Mitochondrial HCO_3_^–^ is essential for pyruvate carboxylase in the gluconeogenic or in lipogenic pathways and for carbamoyl phosphate synthetase I in the ureagenesis process in the liver. This is because HCO_3_^–^ cannot permeate the inner mitochondria membrane and no bicarbonate transporter (SLC4A family) is located on this membrane. Therefore, HCO_3_^–^ supplied by CA VA/VB is thus critical for pyruvate carboxylase to convert pyruvate to oxaloacetate in the mitochondrion. Then, the tricarboxylate transporter transports the oxaloacetate to the cytosol, where it is implicated in the synthesis of fatty acids (lipogenesis).^16^ Furthermore, CA V is also most likely implicated in the glucose-induced secretion of insulin.^17^ There are at least two possible mechanisms by which mitochondrial CAs could participate in the regulation of insulin secretion. In detail, as already mentioned, CA V provides HCO_3_^–^ for pyruvate carboxylase, which is abundantly expressed in the mitochondrial islet cells, and it is important in the pyruvate–malate shuttle, which provides NADPH for normal β-cell functions, like glucose-induced secretion of insulin, as demonstrated by a study of MacDonald et al.^18^ Furthermore, the control of mitochondrial calcium concentrations is a second way through which CA V may be connected to insulin secretion. As observed by Kennedy et al., calcium ions have a fundamental role in the energy requirements for the exocytosis of insulin from β-cell.^19^

In addition, there is clear evidence that the inhibition of both the isoforms VA and VB leads to considerable weight loss.^11,20,21^ Given these considerations, DPP IV and CA (II and V) can be considered good targets to treat concomitantly T2DM and obesity, which are often linked. Therein, a new class of multitarget ligands addressed on DPP IV and CA (II and V) is reported. The derivates have been designed by combining a repurposing strategy and a subsequent morphing process. Indeed, drug repurposing has been recognized as a good tool to speed up the drug discovery and drug development pipeline, while morphing allows molecular properties to be progressively improved through rational drug design.

## Results and Discussion

### Compound Design

The rational design of the multitarget compounds targeting DPP IV and CAs started from the observation that the specific inhibitors for these enzymes share some key features. In detail, nearly all DPP IV inhibitors include a basic center, which interacts with Glu205 and Glu206 of the DPP IV S2 subsite. The presence of amino groups in CA inhibitors is well tolerated and characterizes the structure of some clinically used CA inhibitors (such as brinzolamide and dorzolamide).^22^

Notably, the presence of a basic group seems to favor the CA II selectivity, as evidenced by several CA II inhibitors used for ocular disorders.^23,24^

Likewise, the sulfonamide moiety is a key feature shared by many CA inhibitors due to the chelating properties toward the catalytic Zn^++^ ion of these enzymes. Moreover, the sulfonamide moiety is included in some known DPP IV inhibitors (e.g., fluoroomarigliptin) and X-ray studies revealed that it is accommodated within the extended S2 subsite where it can interact with Arg358.^25,26^

Based on these premises, the first step of our project involved the identification of suitable scaffolds able to include both the amino and the sulfonamide groups in an arrangement that could be convenient for both targets.

Considering that the CA binding sites are generally more promiscuous and can accommodate structurally heterogeneous inhibitors, at the beginning the design was focused on DPP IV. The starting point of our analyses was a recent study,^27^ which evidences a certain degree of similarity between DPP IV inhibitors and adrenergic ligands, as confirmed by the promising DPP IV inhibition of some Ephedra’s alkaloids. Based on our prior experience in the field, we decided to focus on the possibility of repurposing known α_1_-AR antagonists as DPP IV inhibitors.

**Figure 1:**
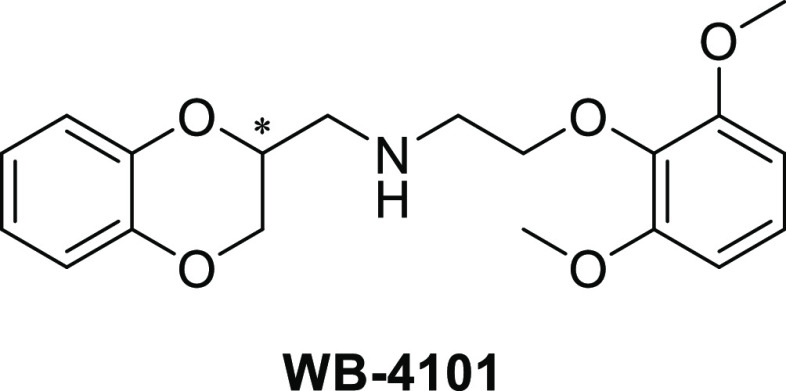
Molecular structures of WB-4101.

**Figure 2:**
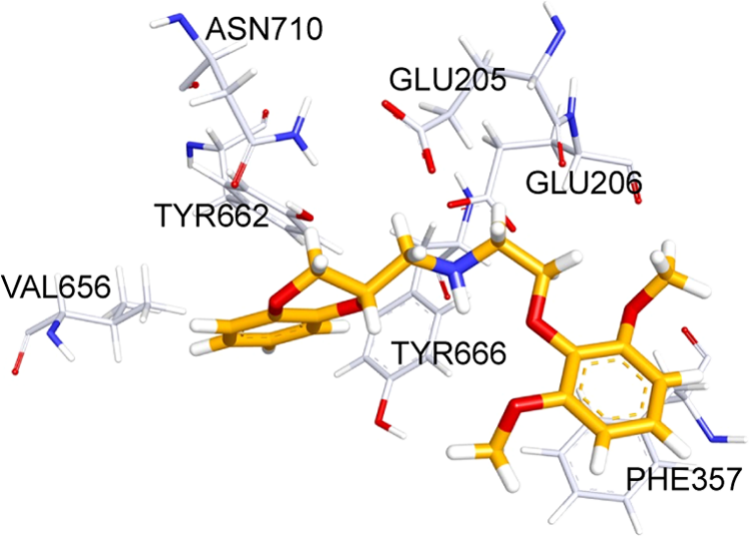
Docking pose of (S)-WB-4101 in the binding site of DPP IV (PDB ID: 1X70).

Among the various amenable α_1_-AR antagonists, we chose as template WB-4101 (2-[(2,6-dimethoxyphenoxyethyl)aminomethyl]-1,4-benzodioxane (Figure 1) for several reasons: (a) preliminary docking simulations showed that it is conveniently harbored within the DPP IV cavity where it elicits clear interactions with some of the key residues of the S1 and S2 subsites (see Figure 2); (b) our laboratory has accumulated significant experience in the synthesis of this compound and of its derivatives;^28−30^ (c) we learned through previous studies that were also confirmed by the literature how to reduce or abrogate the α _1_-AR affinity by substituting the para position of the phenoxy ring.^31,32^

Clearly, we do not expect that this repurposed molecule could be active *per se* on the targets and, however, this should not be actually desirable due to its strong effect on the adrenergic receptors. Thus, we decided to modify WB-4101 as little as possible to gain inhibition toward DPP IV and CAs while losing the adrenergic affinity. These tailored modifications have been introduced by a progressive morphing strategy that provides the major advantage of scanning the specific contribution of each modification besides allowing the modulation of the activity toward the old and new targets.^33,34^ Specifically, the introduced modifications regarded two portions of the structure of WB-4101: the already mentioned para position of the 2,6-dimethoxyphenoxy ring and the secondary amine function.

At first, the para position of the 2,6-dimethoxyphenoxy ring was substituted by the sulfonamide group, which should have a beneficial effect on both CAs and DPP IV while reducing adrenergic affinity.

**Scheme 1:**
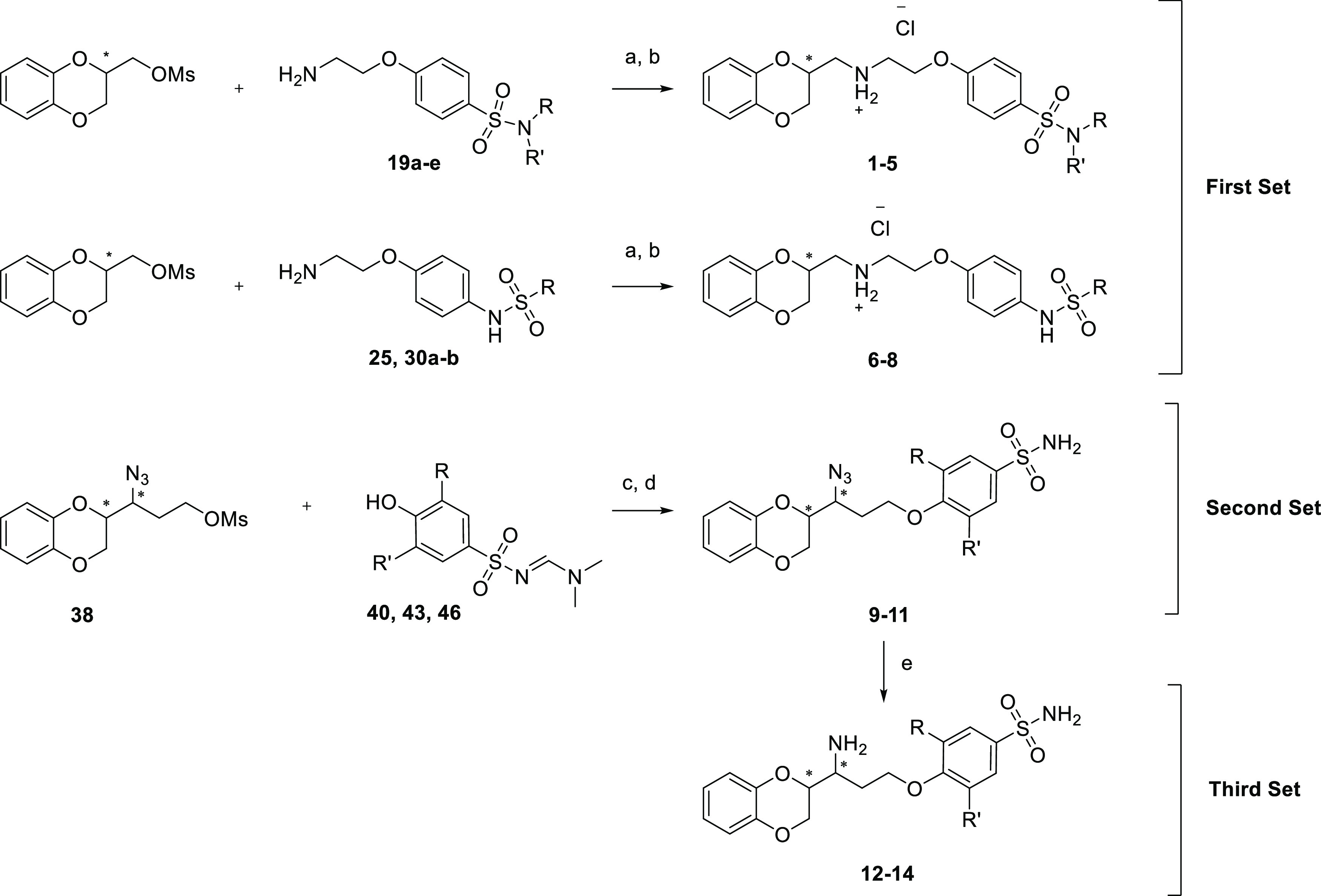
General Synthetic Procedure for Compounds **1**–**14** Reagents and conditions: (a) triethylamine (TEA), isopropyl alcohol (IPA), reflux, (b) diethyl ether hydrochloride, 0 °C, (c) K_2_CO_3_, scaffold **40**, **43**, or **46**, *N*,*N*-dimethylformamide (DMF), 90 °C, (d) NH_2_NH_2_·H_2_O, MeOH, room temperature (RT), and (e) NH_2_NH_2_·H_2_O, PdO, MeOH, reflux.

This modification was supported by preliminary docking simulations on the resolved CA II structure (see Figure S1, Supporting Information), which showed that the sulfonamide group is able to properly chelate Zn^++^ and that the WB-4101 analogue is conveniently accommodated within the CA II cavity where it mostly stabilizes hydrophobic contacts. Similarly, docking simulations on DPP IV revealed that the para-sulfonamide group does not affect the already shown binding mode of WB-4101 even though the introduced sulfonamide moiety fails to properly contact Arg358 (see Figure S2, Supporting Information). We also designed compounds characterized by reversed sulfonamide moieties to elongate the para-substituents in an attempt to reach Arg358. Subsequently, besides maintaining the sulfonamide residue, we decided to transform the secondary amine present in WB-4101 into a primary amine, also designing the corresponding azide derivatives. Such a modification besides being suggested by the observation that gliptins generally include a primary amino group or a nitrile residue has been driven by the continuous feedback coming from biological assays. Therefore, different WB-4101 derivatives have been designed and synthesized. The compounds described therein can be divided into three sets (Scheme 1). Compounds belonging to the first set differ very little from WB-4101 since the two ortho methoxy groups were removed and the aromatic ring was decorated by different *para*-sulfonamide moieties. The substitution in the para position was chosen to abrogate or at least diminish the alpha-adrenoceptor activity, as already demonstrated. The second set covers the compounds that feature the azide group instead of the secondary amine and one, two, or no methoxy group on the phenoxy ring substituted at the para position with a primary sulfonamide. The third set includes the analogues of the second set that are characterized by a primary amine in lieu of the azide function. These modifications were inspired by the promising docking investigation and common features shared by drugs already available on the market.

### Chemistry

According to the structure, the designed compounds can be divided into three sets: the first one includes the derivatives characterized by a secondary amine; the second one comprises the ligands endowed by an azide group; and the third covers the analogues of the second set, which carries a primary amine instead of the azide moiety.

**Scheme 2:**
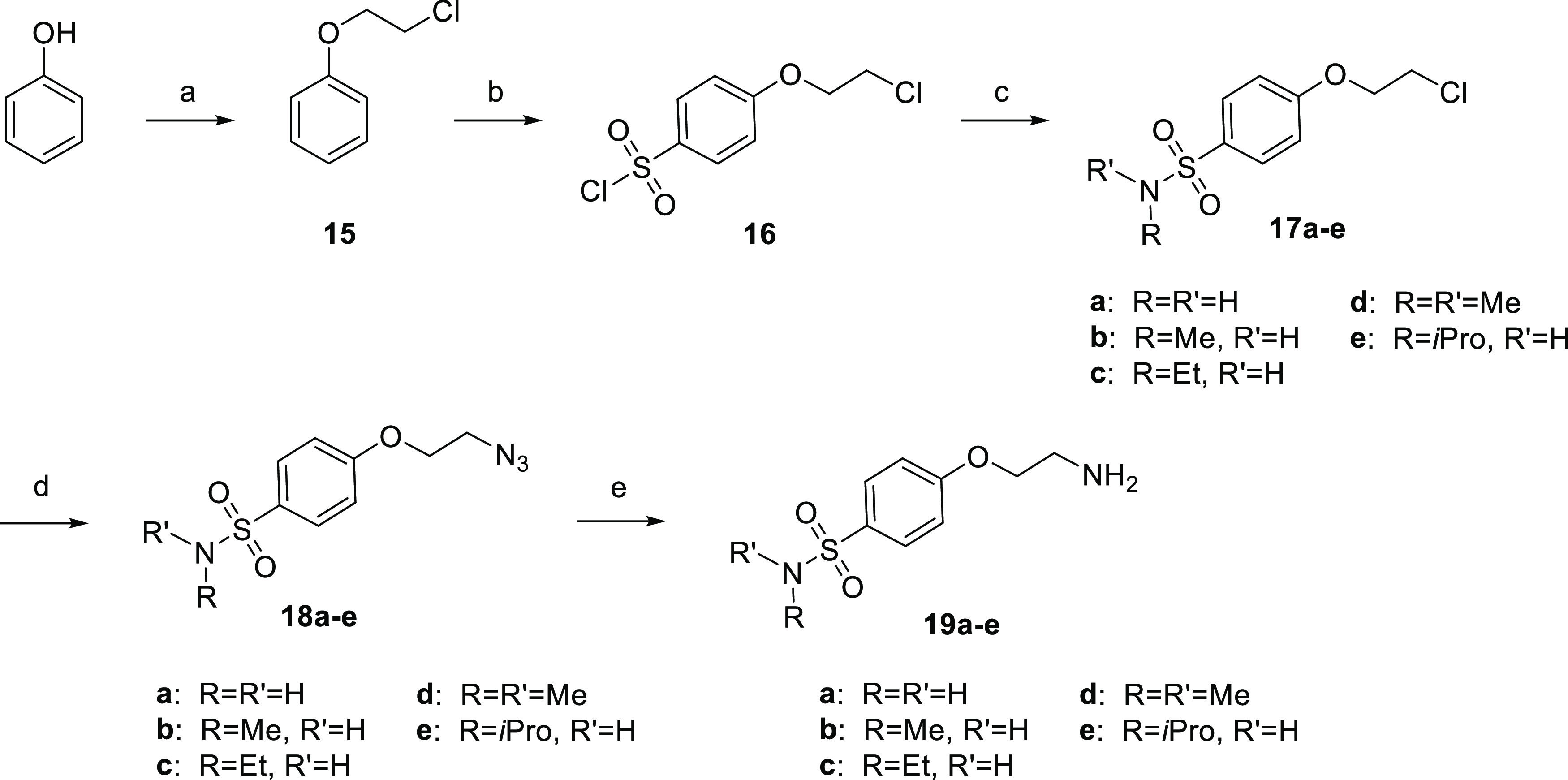
Synthesis of Intermediates **19a**–**e** Reagents and conditions: (a) 1-bromo-2-chloroethane, tetrabutylammonium bromide (TBAB), NaOH, dichloromethane (DCM), RT; (b) chlorosulfonic acid, DCM, −10 °C; (c) HNRR’, tetrahydrofuran (THF), 0 °C; (d) NaN_3_, KI, DMF/H_2_O 3:1, 90 °C; and (e) NH_2_NH_2_·H_2_O, PdO, MeOH, reflux.

For all three sets, the phenoxy ring is substituted at the para position, and for the second and third sets, it is also decorated with one or two methoxy groups. The first set of compounds **1**–**8** was synthesized by *N*-alkylation of 4-(2-aminoethoxy)benzenesulfonamide (**19a**), 4-(2-aminoethoxy)-*N*-methylbenzenesulfonamide (**19b**), 4-(2-aminoethoxy)-*N*-ethylbenzenesulfonamide (**19c**), 4-(2-aminoethoxy)-*N*,*N*-dimethylbenzenesulfonamide (**19d**), 4-(2-aminoethoxy)-*N*-isopropylbenzenesulfonamide (**19e**), *N*-(4-(2-aminoethoxy)phenyl)methanesulfonamide (**25**), *N*-(4-(2-aminoethoxy)phenyl)ethanesulfonamide (**30a**), and *N*-(4-(2-aminoethoxy)phenyl)-2-methylpropan-1-sulfonamide (**30b**) with 2-(*R,S*)-mesyloxymethyl-1,4-benzodioxane.^28^ The second set that includes compounds **9**–**11** was synthesized through a nucleophilic substitution between *N*,*N*-dimethylaminomethylene-4-hydroxybenzenesulfonamide (**40**), *N*,*N*-dimethylaminomethylene-3-methoxy-4-hydroxybenzenesulfonamide (**43**), *N*,*N*-dimethylaminomethylene-3,5-dimethoxy-4-hydroxybenzenesulfonamide (**46**), and 1-azido-1-(1,4-benzodioxan)-propylmethanesulfonate (**38**) followed by the deprotection of the sulfonamide moiety. The third group of derivatives **12**–**14** was obtained through a catalytic reduction of compounds **9**–**11**. The synthesis of the building blocks **19a**–**e** is illustrated in Scheme 2.

**Scheme 3:**
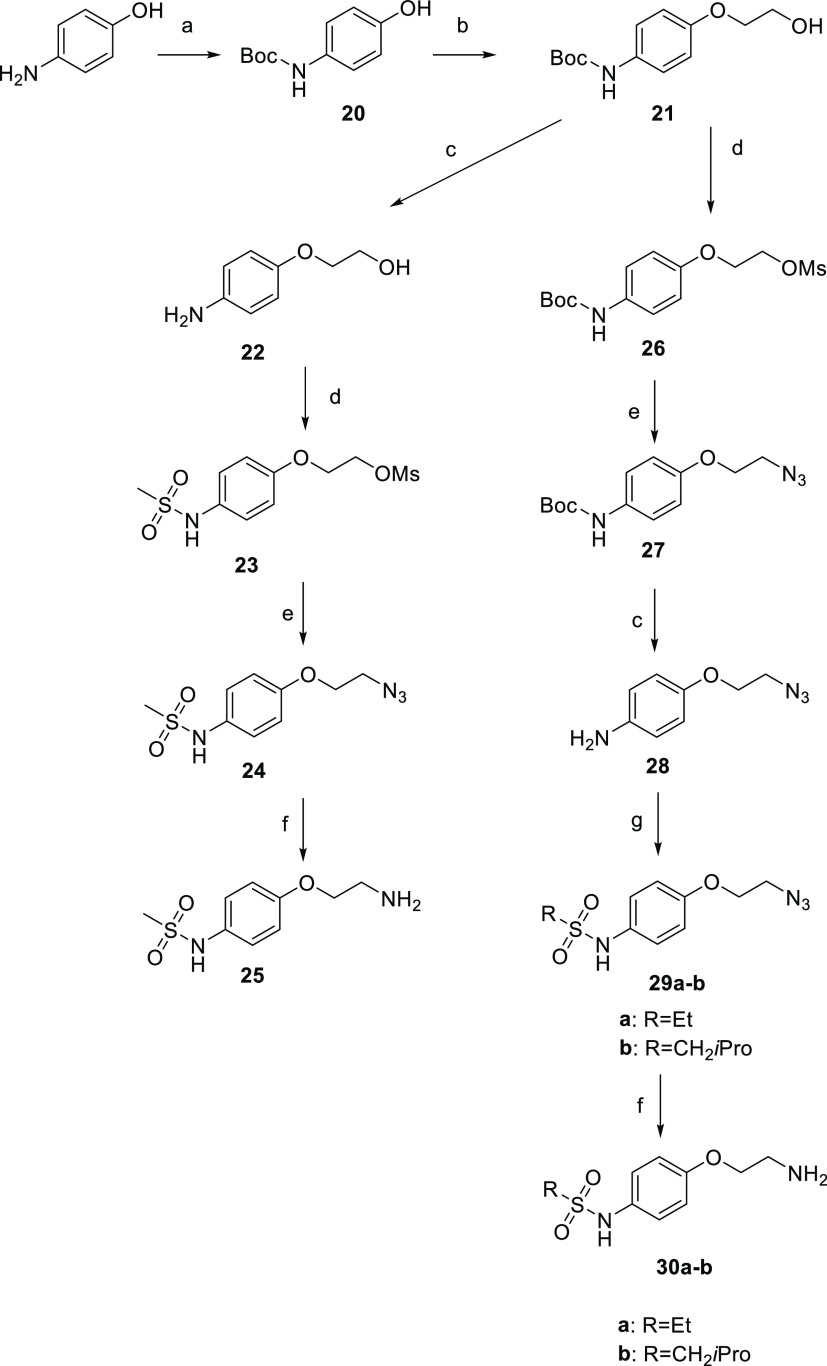
Synthesis of the Intermediates **25** and **30a,b** Reagents and conditions: (a) Boc_2_O, THF, 0 °C; (b) ethylene carbonate, K_2_CO_3_, DMF, reflux; (c) MeOH·HCl, 50 °C; (d) MsCl, TEA, DCM, 0 °C; (e) NaN_3_, KI, DMF/H_2_O 3:1, 90 °C; (f) NH_2_NH_2_·H_2_O, PdO, MeOH, reflux; and (g) RSO_2_Cl, TEA, DCM, 0 °C.

Phenol was reacted with 1-bromo-2-chloroethane to give 2-chloroethoxybenzene (**15**), which underwent an acylation in the para position with chlorosulfonic acid. Afterward, the nucleophilic substitution with different amines gave the corresponding sulfonamide moiety (**17a**–**e**). Through a subsequent substitution with sodium azide followed by a reduction using hydrazine, synthons **19a**–**e** were obtained. The synthesis of building block **25** is reported in Scheme 3.

The synthetic strategy started from the protection of the amino group of commercially available *p*-aminophenol with di-*tert*-butyl dicarbonate (**20**). Subsequently, the hydroxyl moiety underwent an hydroxyalkoxylation to provide *tert*-butyl (4-hydroxyethoxy)phenyl)carbamate (**21**), which is also shared by the synthetic strategy planned to obtain **30a** and **30b**. The synthetic approach used to obtain **25** continued using methanol hydrochloride to deprotect the amino group, which was mesylated with methanesulfonyl chloride as well as the hydroxy group to provide **23**. Then, the nucleophilic substitution with sodium azide and the eventual reduction with hydrazine gave amine **25**.

The synthetic route to obtain **30a** and **30b** started from the shared synthon **21** whose hydroxyl function was mesylated (**26**) and subsequently substituted by sodium azide (**27**).

The deprotection of the amine function (**28**) was achieved by treatment with methanol hydrochloride. Then, through the reaction with ethane- or isobutene-sulfonyl chloride, the sulfonamide synthons (**29a**–**b**) were obtained. The azide function was then reduced with hydrazine giving amine **30a**–**b**, which were condensed with 2-mesyloxymethyl-1,4-benzodioxane giving the free secondary amine derivatives, which gave compounds **7** and **8** after the treatment with diethyl ether hydrochloride.

**Scheme 4:**
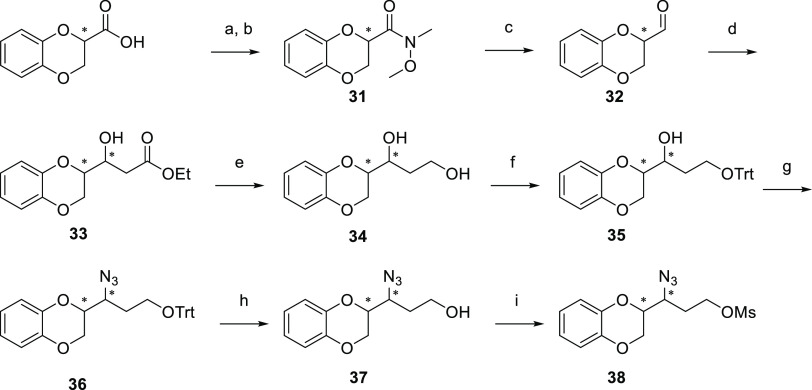
Synthesis of Intermediate **38** Reagents and conditions: (a) SOCl_2_, DCM, reflux; (b) *N*,*O*-dimethylhydroxylamine, DCM, RT; (c) LiAlH_4_, THF, −20 °C; (d) Zn, TBDMSiCl, ethyl bromoacetate, THF, reflux; (e) LiAlH_4_, THF, −10 °C; (f) TrtCl, TEA, DCM, RT; (g) PPh_3_, diethyl azodicarboxylate (DEAD), diphenylphosphorylazide (DPPA), THF, RT; (h) Amberlist 15, DCM/MeOH, reflux; and(i) MsCl, TEA, DCM, RT.

The preparation of compounds **9**–**14** shared a common key **38** for which the synthetic route is illustrated in Scheme 4. The treatment of 1,4-benzodioxan-2-carboxylic acid with *N*,*O*-dimethylhydroxylamine, preceded by the conversion into the corresponding acyl chloride, led to the obtainment of the Weinreb amide (**31**), which was reduced and transformed into **32**. The aldehydic function underwent a Reformatsky reaction with ethyl bromoacetate, accomplishing the β-hydroxy ester (**33**). The reduction of the ester moiety afforded the primary alcohol (**34**), which was protected with trityl chloride (**35**). After the Mitsunobu reaction, which produced the azide derivative (**36**), the deprotection of trityl ether was achieved *via* treatment with Amberlyst 15 (**37**).

**Scheme 5:**
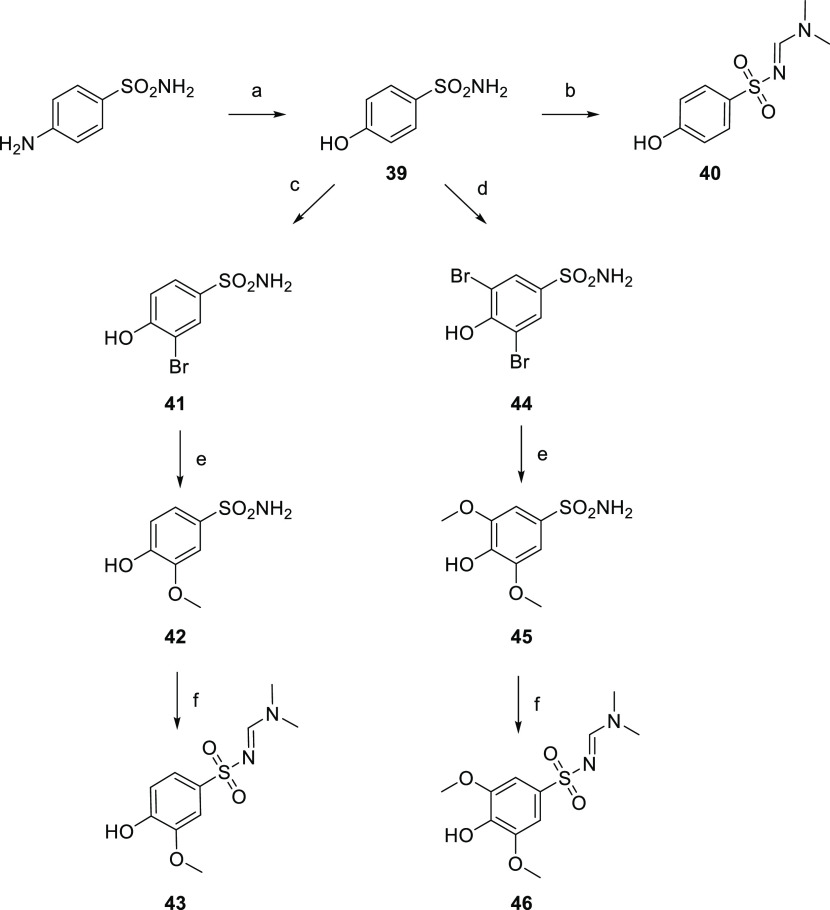
Synthesis of Intermediates **40**, **43**, and **46** Reagents and conditions: (a) NaNO_2_, H_2_SO_4_, H_2_O, reflux; (b) DMF-DMA, DMF, RT; (c) NBS, DMF, 0 °C; (d) Br_2_, DCM/EtOH, RT; (e) Na, CuI, MeOH, reflux; and (f) DMF-DMA, DMF, RT.

Hence, the hydroxyl moiety was mesylated to achieve **38**. The synthesis of intermediate **40** shown in Scheme 5 started from the commercially available sulfonamide whose amine function was converted into a hydroxy one (**39**) *via* the Sandmeyer reaction. Consequently, **40** was prepared from 4-hydroxybenzenesulfonamide by the protection of the sulfonamide moiety as *N*-sulfonylformamidine. The synthetic strategy to achieve scaffold **43** is shown in Scheme 5. Regioselective monobromination of 4-hydroxybenzenesulfonamide gave compound **41**, which was treated with sodium methoxide and CuI at reflux to obtain **42**. Consequently, **43** was achieved *via* protection of the sulfonamide group with *N*,*N*-dimethylformamide dimethyl acetal. The obtainment of scaffold **46**, shown in Scheme 5, started from 4-hydroxybenzenesulfonamide (**39**), which underwent ortho-dibromination. The treatment of intermediate **44** with sodium methoxide and CuI at reflux led to the obtainment of **45**. The protection of the sulfonamide moiety as *N*-sulfonylformamidine accomplished scaffold **46**.

### DPP IV Inhibition

**Figure 3:**
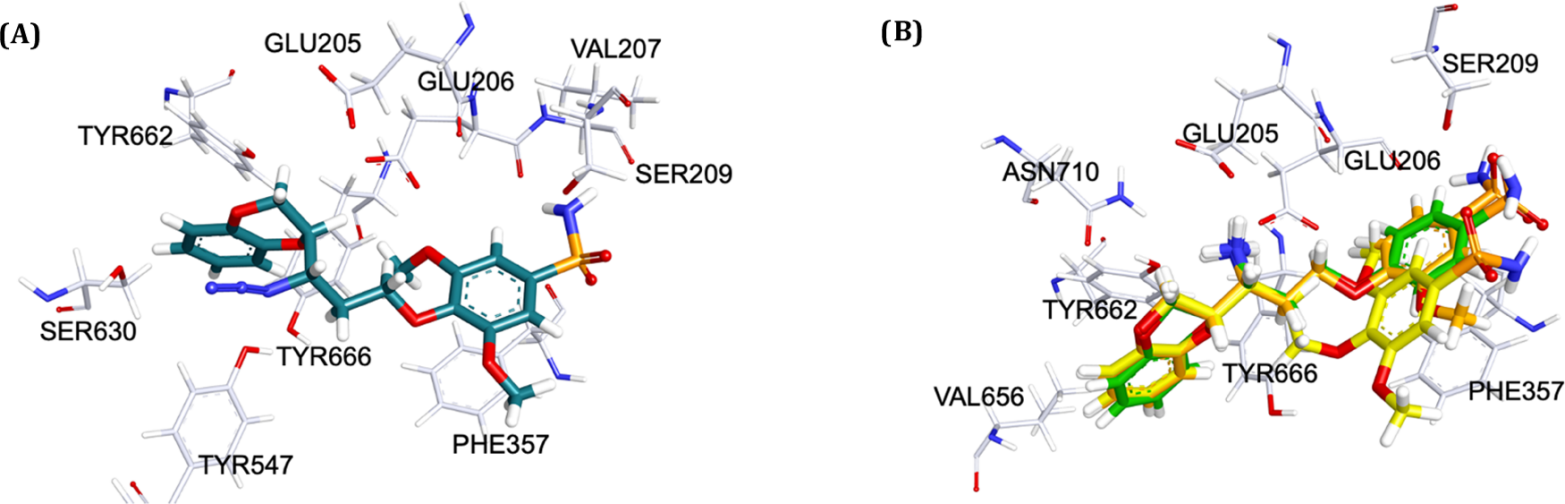
Proposed binding modes of the morphed compounds in the binding site of DPP IV (PDB ID: 1X70). (A) Binding pose of compound (*R*,*S*) **11**. (B) Superimposition of the third group of compounds: (*S*,*R*) **12** (green), (*S*,*R*) **13** (orange), and **14** (*S*,*R*) (yellow).

The DPP IV inhibition activities of the entire sets of compounds were carried out on purified recombinant human DPP IV using conditions previously optimized.^35^ Sitagliptin and WB-4101 were included in the sets for comparison. The inhibition data are listed in Table 1. The group of these multitarget ligands, which carries a secondary amine function (**1**–**8**), does not show a noticeable inhibitory activity for DPP IV. Indeed, the data expressed as micromolar concentration reported in Table 1 highlight the absence of activity toward this enzyme for all derivatives except compounds **6** and **8**, even if their profile is still not satisfactory. This trend can be partially explained by the weaker Glu–dyad interaction established by the secondary amine. Moreover, compounds **5**–**8** that feature reversed sulfonamide moieties appended at the para position of the phenoxy ring are inactive or show weak inhibition as well. Compounds belonging to both the second and the third sets (**9**–**14**), which are characterized by the presence of an azide or a primary amine moiety, show a different trend of inhibition. The biological results display that derivative compounds **9** and **10** weakly inhibit DPP IV, while **11** has a strong activity on DPP IV. The docking results of this compound exhibit a distinctive binding mode with the azide moiety close to the Ser630 (Figure 3A). Compounds **12** and **14** possess a nanomolar potency comparable to that shown by the commercially available DPP IV inhibitor sitagliptin. This strong activity could be explained by the docking poses (Figure 3B); indeed, they assume a conformation that stabilizes all of the key interactions with the binding site like the primary amine group near the two key residues, namely, Glu205 and Glu206.

**Table 1:**
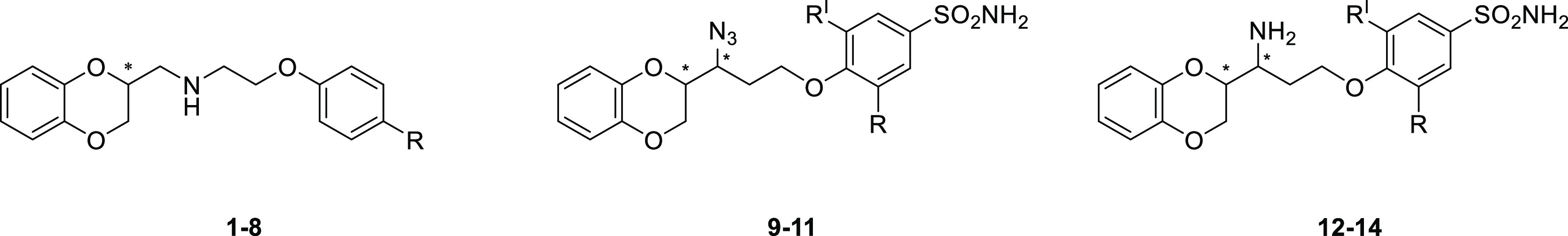
Inhibition Activity of the Tested Compounds for DPP IV and for Human CA Isoforms hCA I, II, IV, VA, VB, and IX

			IC_50_^a^ (μM)	*K_i_*^b^ μM					
compd	R	R^I^	DPP IV	CA I	CA II	CA IV	CA VA	CA VB	CA IX
**1**	–SO_2_NH_2_		>700	0.0849	0.0072	0.2908	0.0779	0.0895	0.0203
**2**	–SO_2_NHCH_3_		>700	>10	>10	>10	>10	>10	>10
**3**	–SO_2_NHCH_2_CH_3_		>700	>10	>10	>10	>10	>10	>10
**4**	–SO_2_N(CH_3_)_2_		>700	>10	>10	>10	>10	>10	>10
**5**	–NHSO_2_*i*Pr		>700	>10	>10	>10	>10	>10	>10
**6**	–NHSO_2_CH_3_		300.2 ± 25.2	>10	>10	>10	>10	>10	>10
**7**	–NHSO_2_CH_2_CH_3_		>700	>10	>10	>10	>10	>10	>10
**8**	–NHSO_2_CH_2_*i*Pr		326.6 ± 10.4	>10	>10	>10	>10	>10	>10
**9**	–H	–H	669.3 ± 23.5	8.7050	0.8625	4.5510	0.6588	0.1828	0.3280
**10**	–OCH_3_	–H	326.6 ± 10.4	0.9718	0.3497	6.1440	0.0775	0.3866	0.1106
**11**	–OCH_3_	–OCH_3_	0.0332 ± 0.0058	6.1340	4.8900	4.5640	0.0896	0.1550	0.7454
**12**	–H	–H	0.0490 ± 0.0045	0.2615	0.0361	3.034	0.0941	0.0428	0.1328
**13**	–OCH_3_	–H	57.44 ± 3.2	0.9140	0.8426	0.4783	0.4560	0.4097	0.0311
**14**	–OCH_3_	–OCH_3_	0.0283 ± 0.0035	5.9600	4.3100	4.8400	4.3460	0.2317	0.9549
WB-4101			>700	>10	>10	>10	>10	>10	>10
Sitagliptin			0.0088 ± 0.0011	>10	>10	>10	>10	>10	>10
AAZ			ND	0.2500	0.0121	0.0740	0.0630	0.0540	0.0257

a Mean from three different assays.

b Mean from three different assays by a stopped-flow technique (errors were in the range of ±5–10% of the reported values), ND; not determined.

However, if we compare the binding pose of all of these derivatives, **12**, **13**, and **14**, we observe an almost perfect overlay between the three molecular structures, making the biological results of **13** quite unexpected. In addition, all inhibitors were further stabilized by π–π stacking interactions with Tyr662 and Phe357. In the case of compound **14**, the decoration of the para benzenesulfonamide with two methoxy moieties reinforces the interaction with Phe357 (Figure 3B), enhancing the inhibitor activity. Furthermore, as assumed, by considering all compounds **9**–**14**, a significant role seems to be played by the substituent on the phenoxy ring since the decoration by the methoxy groups results in a general positive contribution to the inhibition activity.

### CA Inhibition

The CA inhibition profiles of all of the compounds (**1**–**14**) were evaluated on several common isoforms, namely, hCA I, II, IV, V (A and B), and IX. Acetazolamide (AAZ) was used as positive control, while sitagliptin and WB-4101 were included in the sets for comparison. The choice of these isoforms was based on their sequence homology to better evaluate the selectivity profile. An applied photophysics stopped-flow instrument was used for assaying the CA-catalyzed CO_2_ hydration activity.^37^ The results are listed in Table 1 expressed as micromolar concentrations.

The primary sulfonamide moiety of compound **1** shows strong inhibitor activity on the physiologically dominant isoform hCA II (*K_i_* 7.2 nM) with comparable potency to that of the AAZ (*K_i_* of 12.1 nM). Moreover, this compound has also shown a good selectivity profile against hCA II. Derivatives of the second and third groups show affinity comparable to that of compound **1**, thus indicating that the added primary amine, as well as the azide function, does not affect the interaction with hCAs. Compounds **10** and **11** effectively and selectively inhibit the mitochondrial isoform hCA VA with inhibition constants ranging in the nanomolar range between 77.5 and 89.6 nM. Both these compounds bear the azide moiety and have at least one methoxy group on the phenoxy ring. Moreover, dimethoxy derivatives in the second and third generations are generally less efficacious on hCAs in comparison to the compounds of the same wave, leading to the conclusion that this substitution is disadvantageous on the CAs while being quite influential on the DPP IV inhibition. The reasons behind this behavior might be found in the different electronic properties of the sulfonamide moiety, which is now bound to a more electron-rich system, which influences its chelating capability.^38^ Furthermore, the increased steric hindrance due to the two methoxy groups can significantly influence the approach to the protein binding site.^39^ Among this generation, compound **12**, which bears a primary amine moiety and has no decoration on the phenoxy ring, targets hCA II, VA, and VB with selectivity over the cytosolic isozymes (selectivity ratio hCA I/II of 7.24 and hCA IX/II of 3.68) all in the low nanomolar ranges (36.1, 94.1, and 42.8, respectively).

### α_1a_-AR Binding Assay of Active Compounds

The most active compounds that combined DPP IV and CAs (II and/or V) inhibitory activity, namely, **11** and **12**, which, however, derived from repurposing and morphing an adrenergic ligand, were tested on human cloned α_1a_-AR expressed in HEK293 cells. Since WB-4101 is a potent α_1_ antagonist, this adrenoreceptor subtype was chosen to assess the loss of adrenergic affinity.

As highlighted by the data reported in Figure S3 (see the Supporting Information), the compounds did not show activity on α_1a_. More in detail, the data reported the absence of the binding affinity of compound **11** toward α_1a_-AR, while derivative **12** exhibits poor affinity (p*K_i_* of 6.61) for the same subtype. Such results confirm that the incorporation of a moiety in the para position of the phenoxy ring of WB-4101 abrogates or at least lowers the adrenergic affinity.

### ADME Prediction

**Figure 4:**
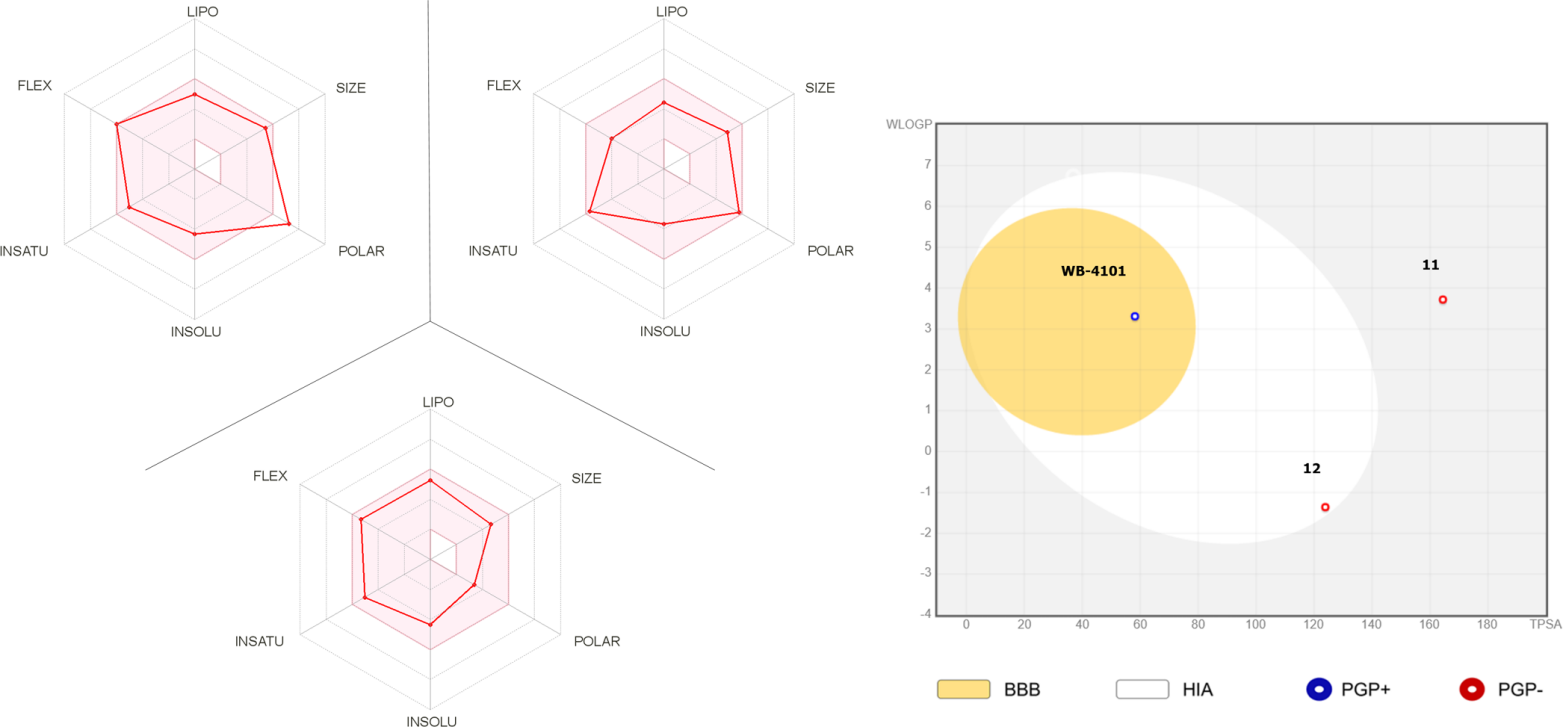
(A) Bioavailability radar plot of compounds **11, 12**, and **WB-4101** (B) using Swiss ADME predictor. Bioavailability radar plot of druglikeness where the pink area characterizes the ideal range for the properties lipophilicity (LIPO), size (SIZE), polarity (POLAR), solubility (INSOLU), saturation (INSATU), and flexibility (FLEX). (B) A boiled-egg graphic where compounds inside the yolk indicate access through the blood–brain barrier (BBB) and those inside white indicate human intestinal absorption (HIA), and blue or red dots represent the prediction of the *P*-glycoprotein substrate (PGP+) or *P*-glycoprotein nonsubstrate (PGP−), respectively.

Pharmacokinetics, druglikeness, and medicinal chemistry friendliness have been evaluated by Swiss ADME.^40^ At a first glance from the bioavailability radar plot (Figure 4), the properties of compound **11** (Figure 4A) are not entirely comprised of the pink area but comparable to drugs containing warheads.

In addition, compound **11** shows very poor gastrointestinal absorption. Conversely, compound **12** shows a high gastrointestinal absorption and it gratifyingly falls into the pink area (Figure 4B) indicating the suitable physicochemical space for oral bioavailability. The ilog *P* (log Po/w) is 0.00, the Xlog P3 (log Po/w) is 2.22, the Wlog *P* (log Po/w) is −1.36, the Mlog *P* (log Po/w) is −3.12, the SILICOS-IT (log Po/w) is 0.93, and the consensus log Po/w is −0.97. Considering the log *P* values, overall compound **12** has a poor lipophilic character. Furthermore, not least of all, compound **12** shows an enhanced druglikeness profile compared to WB-4101 (see Supporting Information Table S1).

**Figure 5:**
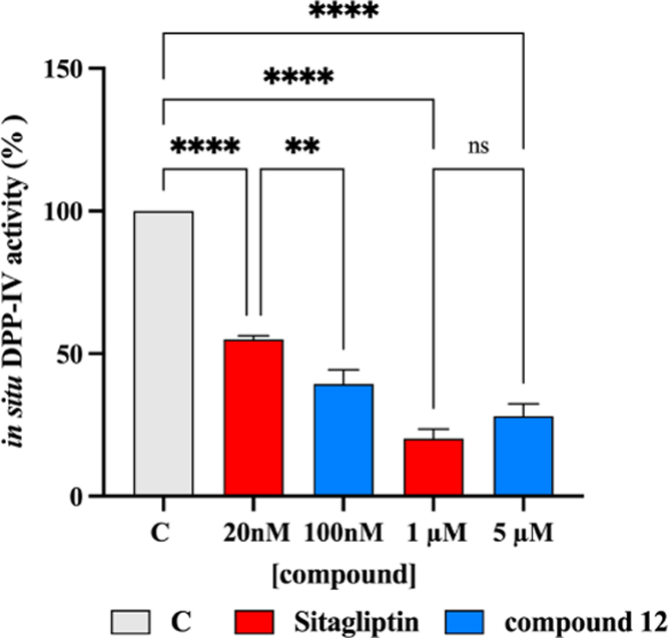
In situ inhibition of the DPP IV activity expressed by nondifferentiated Caco-2 cells after 60 min of treatment. The data are represented as the means ± standard deviation (SD) of three independent experiments, performed in triplicate. Statistical analysis was performed by one-way analysis of variance (ANOVA), followed by Tukey’s post hoc test. ns: not significant; (**), *p* < 0.01, (****) *p* < 0.0001; C: control cells. Red bars: sitagliptin, positive control, at 1.0 μM. Blue bars: compound **12**.

Regarding water solubility, the results coming from the three methods applied by the web tool assess that the screened compounds are water-soluble. Moreover, compound **12** shows high gastrointestinal absorption and does not permeate the blood–brain barrier. In addition, compound **12** is not a *P*-gp substrate; thus, the excretion will not be an issue and it does not inhibit anyone of the isoenzymes, namely, CYP1A2, CYP2C19, CYP2C9, CYP2D6, and CYP3A4, considered by Swiss ADME, thus excluding the chance of accumulation, drug–drug interaction, and subsequent toxicity. Additionally, compound **12** reports no PAINS alerts. The MetaClass^41^ approach suggests a reasonable metabolic stability for compound **12** since it identifies only two possible reactions, namely, redox reactions on carbon atoms and acetylation of the primary amino group. Based on the overall data collected, compound **12** was selected for additional assays to frame it as a lead compound of this novel class of multitarget ligands. As a first step, compound **12** was tested in Caco-2 cells that express high levels of DPP IV on their cellular membranes.^42^ Experiments were performed using sitagliptin as a reference compound. Notably, considering that, as indicated in Table 1, the sitagliptin is almost five times more active than compound **12;** the concentrations used for the cell-based evaluation were 20.0 nM and 1.0 μM for sitagliptin, and 100.0 nM and 5.0 μM for compound **12**. Figure 5 indicates that sitagliptin inhibited cellular DPP IV activity by 44.93 ± 1.21 and 79.69 ± 3.23% at 20 nM and 1.0 μM, respectively, *vs* untreated cells, whereas compound **12** inhibited cellular DPP IV activity by 60.65 ± 4.92 and 71.86 ± 4.27% at 100.0 nM and 5.0 μM, respectively, *vs* untreated cells (Figure 5). Statistical analysis indicated that at a higher concentration no significant differences were observed between compound **12** and sitagliptin. The encouraging results, afforded by Caco-2 cells, confirm the activity on DPP IV even in a more complex environment.

#### In Vitro Pre-ADME/Tox Profiling

##### Solubility

The solubility of compound **12** was determined in phosphate-buffered saline at a pH of 7.4 through a suitable liquid chromatography and tandem mass spectrometry (LC-MS/MS) method, as described in the Experimental Section. Compound **12** shows a soluble concentration of 224 μM, which satisfies one of the prerequisites for good bioavailability to encourage oral dosing as the possible route of administration.

##### Hepatic Microsome Stability

**Figure 6:**
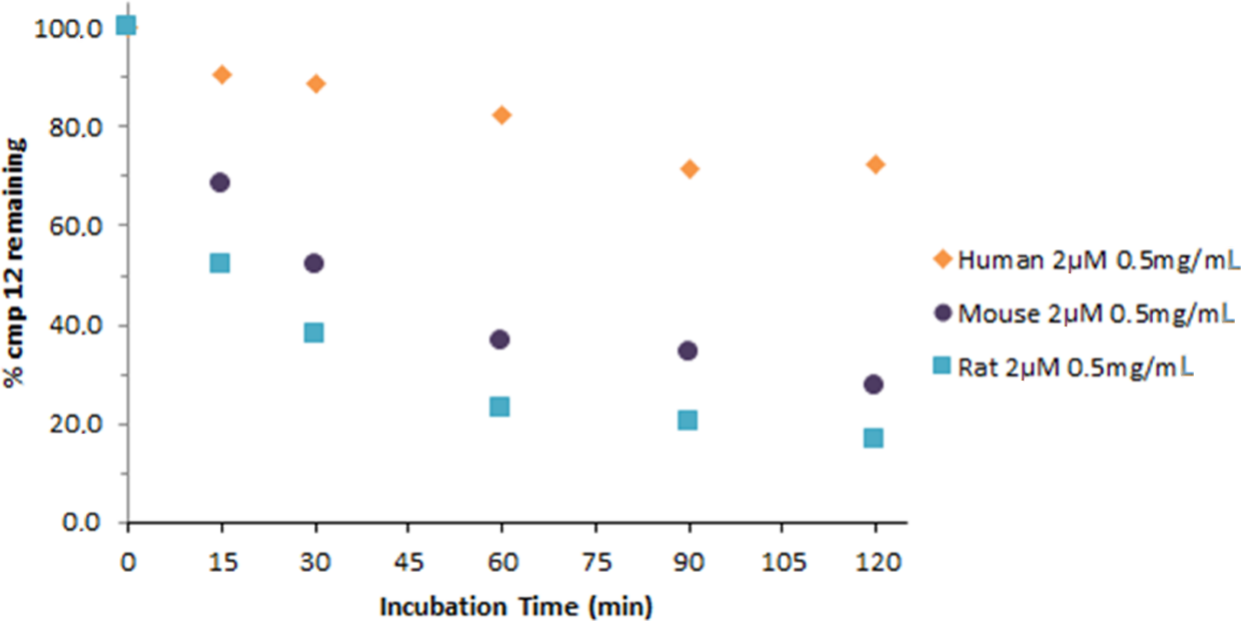
Metabolic stability in rat, mouse, and human liver microsomes of compound **12**.

To predict the metabolic fate as well as the susceptibility of compound **12** to phase I metabolism following the *in vivo* administration, it was incubated with human (Sekisui XenoTech, LLC), CD-1 mouse (BioIVT), and Sprague-Dawley (SD) rat liver microsomes (Corning), as described in the Experimental Section. As reported in Figure 6, compound **12** exhibited modest stability in mouse and rat microsomes, with 27.5 and 16.7% of the compound remaining, respectively, after 120 min of incubation. In human liver microsomes, compound **12** exhibited very high stability, with 72.6% remaining after 120 min of incubation.

#### Permeability

**Figure 7:**
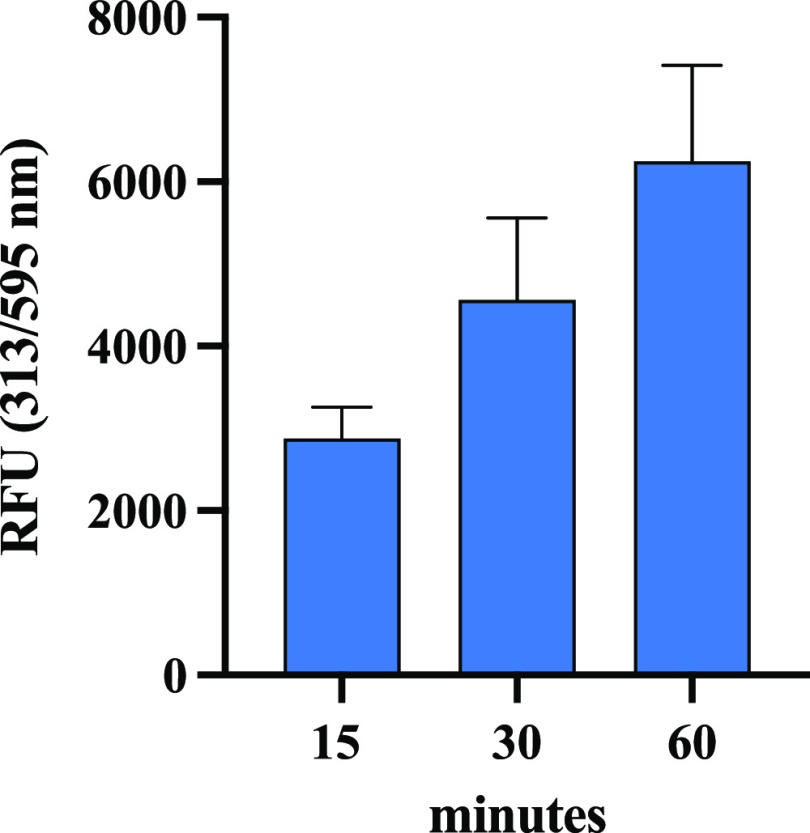
Fluorescence signals (bars normalized using the Janus Green staining) of human intestinal Caco-2 cells, which internalized compound **12** as a function of time.

The ability of compound **12** to be uptaken by Caco-2 cells was assessed, exploiting its natural behavior to emit fluorescence at 313 nm after excitation at 250 nm using a fluorescent plate reader. Notably, Caco-2 cells were treated with compound **12** at 1.0 μM or vehicle for 15, 30, and 60 min. Fluorescence signals emitted at 313 nm by the compound localized at the intracellular level as a function of time have been normalized for the total amount of cells stained using Janus Green (OD 595 nm); therefore, the relative fluorescence unit (RFU) 313/595 nm after 15, 30, and 60 min of incubation was calculated. The RFU 313/595 nm of the blank samples, which represents the cellular background, was subtracted from each normalized fluorescence signal. Results showed that compound **12** successfully permeates the human intestinal cells as a function of time. In particular, compound **12** is absorbed by Caco-2 cells up to 2874 ± 383.1, 4562 ± 993.2, and 6250 ± 1163RFU after 15, 30, and 60 min, respectively (Figure 7).

#### Cytotoxicity

The cytotoxicity of the active compound **12** was determined. Cell viability was assessed by the quantitative colorimetric method of the MTT reduction on Caco-2 and HepG2 cells. As reported in Figure S4 (see the Supporting Information), both the cell lines maintain their metabolic activity when treated with compound **12**.

## Conclusions

T2DM is steadily growing in high-income countries as well as in low-income countries. Moreover, the numbers of patients who suffer from T2DM parallel those affected by obesity, giving reasons for reframing the treatment of T2DM. Thus, new therapeutic approaches able to target diabesity are needed. Given the relevance of the DPP IV/CA (II and V) roles in both T2DM and obesity, multitarget ligands able to modulate these enzymes could represent a novel and promising therapeutic approach. Repositioning of WB-4101 and morphing its structure have been the strategies applied to obtain potent and selective multitarget ligands against DPP IV and CAs (isoforms II and V). In this context, rational multitarget optimization strategies have relied on computational models and systematic exploration of the structure–activity relationship (SAR) on each individual target. The designed sets of molecules, which result from the morphing of the phenoxy ring and the variation of the secondary amine function of WB-4101, can be roughly divided into three generations. All of the compounds have been synthesized and investigated for their inhibitory activity on the selected targets, namely, CA II, V, DPP IV, and off-targets (CA I, IV, and IX). The first generation of derivatives possesses no multitarget modulatory efficacy, despite a remarkable CA II inhibitor potency shown by compound **1**. Compound **11** from the second set and compounds **13** and **14**, which belong to the third group, inhibit DPP IV at nanomolar concentrations, which are comparable to sitagliptin. In addition, they show an interesting CA inhibitor profile, enriched by satisfactory selectivity. On the basis of the structure–activity relationships, a primary amine function closely connected to a rigid substructure, which is, in turn, joined to a *p*-hydroxybenzenesulfonamide portion, results in a crucial feature, to adequately modulate the selected enzymes.

The satisfactory DPP IV inhibition and the selectivity CA profile together with the acceptable ADME prediction confirmed by *in vitro* pre-ADME/Tox profiling indicate compound **12** as the potential lead of this novel class of multitarget ligands even if the design and synthesis of other new morphed derivatives of WB-4101 will help to further understand the key recognition features of the target enzymes. Albeit the characteristics, the issue of fixed activity as a multitarget ligand must be carefully considered to maintain the modulation capacity over the selected targets.

The design strategy applied in this study emphasizes that repurposing analyses can also be successfully used as a preliminary step to identify druglike starting scaffolds, which are amenable to further improvements by finely tuned and progressive modifications.

This new class of molecules paves the way for the development of a promising and reframed therapeutic approach for the treatment of T2DM and obesity.

## Experimental Section

### Chemistry

All chemicals and solvents were purchased from Merck KGaA, Darmstadt, Germany, and TCI and used as commercially distributed. All purifications were performed by flash chromatography using prepacked Biotage Sfär columns or silica gel (particle size 40–63 μm, Merck) on an Isolera (Biotage, Uppsala, Sweden) apparatus. Thin-layer chromatography (TLC) analyses were performed on aluminum plates precoated with silica gel 60 matrix with a fluorescent indicator and visualized in a TLC UV cabinet followed by an appropriate staining reagent. The content of solvents in eluent mixtures is given as v/v percentage. *R_f_* values are given for guidance. ^1^H NMR (300 MHz) and ^13^C NMR (75 MHz) spectra were recorded on a Varian NMR System 300 MHz spectrometer. Chemical shifts (δ) are reported in parts per million (ppm) relative to the residual solvent (CHCl_3_, MeOH, or dimethyl sulfoxide (DMSO)) as an internal standard. Melting points were determined by a Buchi Melting Point B-540 apparatus. The purity determination for all of the final compounds was performed on a high-performance liquid chromatography (HPLC) Varian ProStar liquid chromatography system (Varian) consisting of a solvent delivery module model 240 and a diode array detector Varian ProStar 335. Varian (Galaxy 1.9.3.2) software was used to calculate the chromatographic parameters and to record the chromatograms. The elution conditions for each analysis are reported in the Supporting Information (SI). The purity of all tested compounds was higher than 95% and was determined on a Phenomenex Gemini C_18_ column (250 mm, 4.6 mm, 5 μm, Phenomenex). High-resolution mass spectra were acquired with an LTQ Orbitrap XL mass spectrometer (Thermo Scientific, Milan, Italy) equipped with an ESI source: full MS spectra were acquired in profile mode by the FT analyzer in a scan range of *m*/*z* 120–800, using a resolution of 30,000 full width at half-maximum (FWHM) at *m*/*z* 400. The purity for all the compounds was >95%.

#### [((2-(4-Sulfonamidephenoxy)ethyl)amino)-methyl]-1,4-benzodioxane Hydrochloride (**1**)

4-(2-Aminoethoxy)benzenesulfonamide **19a** (414 mg, 1.91 mmol) and 2-mesyloxymethyl-1,4-benzodioxane (425 mg, 1.74 mmol) were dissolved in 20 mL of 2-propanol. Triethylamine (0.24 mL, 1.74 mmol) was added to the solution, and the resulting mixture was refluxed, under stirring, until TLC indicated the disappearance of the starting material. Afterward, the solvent was evaporated in vacuo, the crude was dissolved in ethyl acetate, and filtered. The filtrate was washed with a 10% aqueous solution of NaHCO_3_ and water. The organic phase was dried over anhydrous sodium sulfate, filtered, and evaporated in vacuo, affording a yellow oil. Flash chromatography (dichloromethane/methanol 95:5 + 0.5% aq. NH_3_) was performed to obtain 67 mg (0.18 mmol) of the pure [((2-(4-sulfonamidephenoxy)ethyl)amino)-methyl]-1,4-benzodioxane as a light yellow oil. TLC (dichloromethane/methanol 95:5 + 1% aq. NH_3_); *R_f_* = 0.35. ^1^H NMR (CDCl_3_): δ 7.86 (d, *J* = 8.9 Hz, 2H), 6.98 (d, *J* = 8.9 Hz, 2H), 6.90–6.81 (m, 4H), 4.78 (s, 2H), 4.32–4.24 (m, 2H), 4.14 (t, *J* = 5.1 Hz, 2H), 4.04 (dd, *J* = 11.6, 7.6 Hz, 1H), 3.10 (t, *J* = 5.1 Hz, 2H), 2.98 (dd, *J* = 8.8, 5.7 Hz, 2H). A solution of the amine (67 mg, 0.18 mmol) in 2.5 mL of 2.0 M hydrogen chloride solution in diethyl ether was stirred overnight. The reaction mixture was diluted with ethyl ether, and the solid was filtered and washed with cooled ethyl ether to obtain the desired product **1** as a pale yellow solid (35.57 mg, 0.09 mmol). Mp 232 °C. HRMS: *m*/*z* 365.1165 [M + H]^+^. ^1^H NMR (CD_3_OD): δ 7.87 (d, *J* = 8.5 Hz, 2H), 7.14 (d, *J* = 8.5 Hz, 2H), 7.00–6.80 (m, 4H), 4.63 (ddt, *J* = 9.4, 6.4, 2.3 Hz, 1H), 4.41 (t, *J* = 4.9 Hz, 2H), 4.35 (dd, *J* = 11.6, 2.3 Hz, 1H), 4.06 (dd, *J* = 11.6, 6.4 Hz, 1H), 3.62 (t, *J* = 4.9 Hz, 2H), 3.56–3.37 (m, 2H). ^13^C NMR (CD_3_OD): δ 160.51, 127.95, 121.94, 121.69, 117.14, 117.03, 114.33, 69.09, 64.74, 63.11.

#### [((2-(4-Methylsulfonamidephenoxy)ethyl)amino)-methyl]-1,4-benzodioxane Hydrochloride (**2**)

4-(2-Aminoethoxy)-*N*-methylbenzenesulfonamide **19b** (170 mg, 0.74 mmol) and 2-mesyloxymethyl-1,4-benzodioxane (164 mg, 0.67 mmol) were dissolved in 7 mL of 2-propanol. Triethylamine (0.093 mL, 0.67 mmol) was added to the solution, and the resulting mixture was refluxed, under stirring, until TLC indicated the disappearance of the starting material. Afterward, the solvent was evaporated in vacuo, and the crude was dissolved in dichloromethane and washed with a 10% aqueous solution of NaHCO_3_ and water. The organic phase was dried over anhydrous sodium sulfate, filtered, and evaporated in vacuo, affording a yellow oil. Flash chromatography (dichloromethane/methanol 90:10 + 1% aq. NH_3_) was performed to obtain 66 mg (0.17 mmol) of the pure [((2-(4-methylsulfonamidephenoxy)ethyl)amino)-methyl]-1,4-benzodioxane as a light yellow oil. TLC (dichloromethane/methanol 95:5 + 1% aq. NH_3_); *R_f_* = 0.40. ^1^H NMR (CDCl_3_): δ 7.78 (d, *J* = 8.9 Hz, 2H), 6.98 (d, *J* = 8.9 Hz, 2H), 6.90–6.79 (m, 4H), 5.05 (s, 1H), 4.32–4.24 (m, 2H), 4.14 (t, *J* = 5.1 Hz, 2H), 4.04 (dd, *J* = 11.6, 7.6 Hz, 1H), 3.10 (t, *J* = 5.1 Hz, 2H), 2.98 (dd, *J* = 8.8, 5.7 Hz, 2H), 2.59 (s, 3H). A solution of the amine (66 mg, 0.17 mmol) in 2 mL of 2.0 M hydrogen chloride solution in diethyl ether was stirred overnight. The reaction mixture was diluted with ethyl ether, and the solid was filtered and washed with cooled ethyl ether to obtain the desired product **2** as a pale yellow solid (20.24 mg, 0.05 mmol). Mp 161 °C. HRMS: *m*/*z* 379.1318 [M + H]^+^. ^1^H NMR (CD_3_OD): δ 7.80 (d, *J* = 8.9 Hz, 2H), 7.18 (d, *J* = 8.9 Hz, 2H), 6.99–6.83 (m, 4H), 4.64 (m, 1H), 4.41 (t, *J* = 4.9 Hz, 2H), 4.35 (dd, *J* = 11.6, 2.4 Hz, 1H), 4.06 (dd, *J* = 11.6, 6.4 Hz, 1H), 3.62 (t, *J* = 4.9 Hz, 2H), 3.58–3.36 (m, 2H), 2.50 (s, 3H). ^13^C NMR (CD_3_OD): δ 160.93, 142.94, 141.59, 132.03, 129.02, 121.90, 121.66, 117.17, 117.02, 114.60, 69.10, 64.77, 63.17, 27.77.

#### [((2-(4-Ethylsulfonamidephenoxy)ethyl)amino)-methyl]-1,4-benzodioxane Hydrochloride (**3**)

4-(2-Aminoethoxy)-*N*-ethylbenzenesulfonamide **19c** (428 mg, 1.75 mmol) and 2-mesyloxymethyl-1,4-benzodioxane (389 mg, 1.59 mmol) were dissolved in 18 mL of 2-propanol. Triethylamine (0.22 mL, 1.59 mmol) was added to the solution, and the resulting mixture was refluxed, under stirring, until TLC indicated the disappearance of the starting material. Afterward, the solvent was evaporated in vacuo, and the crude was dissolved in dichloromethane and washed with a 10% aqueous solution of NaHCO_3_ and water. The organic phase was dried over anhydrous sodium sulfate, filtered, and evaporated in vacuo, affording a yellow oil. Flash chromatography (dichloromethane/methanol 97:3 + 0.5% aq. NH_3_) was performed to obtain 124 mg (0.32 mmol) of the pure [((2-(4-ethylsulfonamidephenoxy)ethyl)amino)-methyl]-1,4-benzodioxane as an orange oil. TLC (dichloromethane/methanol 95:5 + 0.5% aq. NH_3_); *R_f_* = 0.50. ^1^H NMR (CDCl_3_): δ 7.79 (d, *J* = 8.9 Hz, 2H), 6.98 (d, *J* = 8.9 Hz, 2H), 6.87 (m, 4H), 4.32–4.24 (m, 2H), 4.14 (t, *J* = 5.1 Hz, 2H), 4.04 (dd, *J* = 11.6, 7.6 Hz, 1H), 3.10 (t, *J* = 5.1 Hz, 2H), 3.06–2.90 (m, 4H), 1.10 (t, *J* = 7.2 Hz, 3H). A solution of the amine (124 mg, 0.32 mmol) in 4 mL of 2.0 M hydrogen chloride solution in diethyl ether was stirred overnight. The reaction mixture was diluted with ethyl ether, and the solid was filtered and washed with cooled ethyl ether to obtain the desired product **3** as a pale yellow solid (65.58 mg, 0.15 mmol). Mp 167 °C. HRMS: *m*/*z* 393.1473 [M + H]^+^. ^1^H NMR (CD_3_OD): δ 7.81 (d, *J* = 8.9 Hz, 2H), 7.17 (d, *J* = 8.9 Hz, 2H), 7.02–6.82 (m, 4H), 4.64 (m, 1H), 4.41 (t, *J* = 4.9 Hz, 2H), 4.35 (dd, *J* = 11.6, 2.4 Hz, 1H), 4.06 (dd, *J* = 11.6, 6.4 Hz, 1H), 3.62 (t, *J* = 4.9 Hz, 2H), 3.58–3.36 (m, 2H), 2.86 (q, *J* = 7.1 Hz, 2H), 1.05 (t, *J* = 7.1 Hz, 3H). ^13^C NMR (CD_3_OD): δ 160.89, 142.93, 141.62, 133.20, 128.83, 121.87, 121.66, 117.23, 116.99, 114.64, 69.11, 64.81, 63.20, 37.58, 13.81.

#### [((2-(4-Dimethylsulfonamidephenoxy)ethyl)amino)-methyl]-1,4-benzodioxane Hydrochloride (**4**)

4-(2-Aminoethoxy)-*N*,*N*-dimethylbenzenesulfonamide **19d** (261 mg, 1.07 mmol) and 2-mesyloxymethyl-1,4-benzodioxane (238 mg, 0,97 mmol) were dissolved in 10 mL of 2-propanol. Triethylamine (0.13 mL, 0.97 mmol) was added to the solution, and the resulting mixture was refluxed, under stirring, until TLC indicated the disappearance of the starting material. Afterward, the solvent was evaporated in vacuo, and the crude was dissolved in dichloromethane and washed with a 10% aqueous solution of NaHCO_3_ and water. The organic phase was dried over anhydrous sodium sulfate, filtered, and evaporated in vacuo, affording an orangish oil. Flash chromatography (dichloromethane/methanol 97:3 + 0.5% aq. NH_3_) was performed to obtain 96 mg (0.24 mmol) of the pure [((2-(4-ethylsulfonamidephenoxy)ethyl)amino)-methyl]-1,4-benzodioxane 22 as an orange oil. TLC (dichloromethane/methanol 95:5 + 0.5% aq. NH_3_); *R_f_* = 0.61. ^1^H NMR (CDCl_3_): δ 7.71 (d, *J* = 8.9 Hz, 2H), 7.01 (d, *J* = 8.9 Hz, 2H), 6.91–6.80 (m, 4H), 4.36–4.24 (m, 2H), 4.14 (t, *J* = 5.1 Hz, 2H), 4.04 (dd, *J* = 11.6, 7.6 Hz, 1H), 3.10 (t, *J* = 5.1 Hz, 2H), 2.98 (dd, *J* = 8.8, 5.7 Hz, 2H), 2.68 (s, 6H). A solution of the amine (96 mg, 0.24 mmol) in 3 mL of 2.0 M hydrogen chloride solution in diethyl ether was stirred overnight. The reaction mixture was diluted with ethyl ether, and the solid was filtered and washed with cooled ethyl ether to obtain the desired product **4** as a pale yellow solid (79.94 mg, 0.19 mmol). Mp 177 °C. HRMS: *m*/*z* 393.1475 [M + H]^+^. ^1^H NMR (CD_3_OD): δ 7.76 (d, *J* = 8.9 Hz, 2H), 7.22 (d, *J* = 8.9 Hz, 2H), 7.01–6.81 (m, 4H), 4.62 (m, 1H), 4.41 (t, *J* = 4.9 Hz, 2H), 4.35 (dd, *J* = 11.6, 2.3 Hz, 1H), 4.06 (dd, *J* = 11.6, 6.4 Hz, 1H), 3.64 (t, *J* = 4.9 Hz, 2H), 3.57–3.37 (m, 2H), 2.65 (s, 6H). ^13^C NMR (CD_3_OD): δ 161.28, 142.94, 141.58, 129.78, 127.94, 121.91, 121.67, 117.16, 117.02, 114.68, 9.11, 64.77, 63.20, 36.87.

#### [((2-(4-Isopropylsulfonamidephenoxy)ethyl)amino)-methyl]-1,4-benzodioxane Hydrochloride (**5**)

4-(2-Aminoethoxy)-*N*-isopropylbenzenesulfonamide **19e** (718 mg, 2.78 mmol) and 2-mesyloxymethyl-1,4-benzodioxane (617 mg, 2.53 mmol) were dissolved in 30 mL of 2-propanol. Triethylamine (0.35 mL, 2.53 mmol) was added to the solution, and the resulting mixture was refluxed, under stirring, until TLC indicated the disappearance of the starting material. Afterward, the solvent was evaporated in vacuo, and the crude was dissolved in dichloromethane and washed with a 10% aqueous solution of NaHCO_3_ and water. The organic phase was dried over anhydrous sodium sulfate, filtered, and evaporated in vacuo, affording an orangish oil. Flash chromatography (dichloromethane/methanol 97:3 + 0.5% aq. NH_3_) was performed to obtain 30 mg (0.07 mmol) of the pure [((2-(4-isopropylsulfonamidephenoxy)ethyl)amino)-methyl]-1,4-benzodioxane as a pale yellow oil. TLC (dichloromethane/methanol 95:5 + 0.5% aq. NH_3_); *R_f_* = 0.60. ^1^H NMR (CDCl_3_): δ 7.80 (d, *J* = 8.9 Hz, 2H), 6.98 (d, *J* = 8.9 Hz, 2H), 6.91–6.80 (m, 4H), 4.36–4.24 (m, 2H), 4.14 (t, *J* = 5.1 Hz, 2H), 4.04 (dd, *J* = 11.6, 7.6 Hz, 1H), 3.43 (sep, *J* = 6.6 Hz, 1H), 3.10 (t, *J* = 5.1 Hz, 2H), 2.98 (dd, *J* = 8.8, 5.7 Hz, 2H), 1.07 (d, *J* = 6.6 Hz, 6H). A solution of the amine (30 mg, 0.07 mmol) in 2.5 mL of 2.0 M hydrogen chloride solution in diethyl ether was stirred overnight. The reaction mixture was diluted with ethyl ether, and the solid was filtered and washed with cooled ethyl ether to obtain the desired product **5** as a pale yellow solid (18.95 mg, 0.04 mmol). Mp 169 °C HRMS: *m*/*z* 407.1639 [M + H]^+^. ^1^H NMR (CD_3_OD): δ 7.83 (d, *J* = 8.9 Hz, 2H), 7.16 (d, *J* = 8.9 Hz, 2H), 7.00–6.83 (m, 4H), 4.63 (m, 1H), 4.41 (t, *J* = 4.9 Hz, 2H), 4.35 (dd, *J* = 11.6, 2.3 Hz, 1H), 4.06 (dd, *J* = 11.6, 6.4 Hz, 1H), 3.64 (t, *J* = 4.9 Hz, 2H), 3.57–3.37 (m, 2H), 3.30 (sep, *J* = 6.6 Hz, 1H), 1.01 (d, *J* = 6.6 Hz, 6H). ^13^C NMR (CD_3_OD): δ 160.74, 142.94, 141.58, 134.69, 128.75, 121.92, 121.67, 117.15, 117.02, 114.49, 69.10, 64.75, 63.13, 45.43, 22.31.

#### [((2-(4-Methylsulfonylamidephenoxy)ethyl)amino)-methyl]-1,4-benzodioxane Hydrochloride (**6**)

*N*-(4-(2-Aminoethoxy)phenyl)methanesulfonamide **25** (238 mg, 1.03 mmol) and 2-mesyloxymethyl-1,4-benzodioxane (229 mg, 0.94 mmol) were dissolved in 10 mL of 2-propanol. Triethylamine (0.13 mL, 0.94 mmol) was added to the solution, and the resulting mixture was refluxed, under stirring, until TLC indicated the disappearance of the starting material. Afterward, the solvent was evaporated in vacuo, and the crude was dissolved in dichloromethane and washed with a 10% aqueous solution of NaHCO_3_ and water. The organic phase was dried over anhydrous sodium sulfate, filtered, and evaporated in vacuo, affording an orangish oil. Flash chromatography (dichloromethane/methanol 98:2 + 0.5% aq. NH_3_) was performed to obtain 61 mg (0.16 mmol) of the pure [((2-(4-methylsulfonylamidephenoxy)ethyl)amino)-methyl]-1,4-benzodioxane as a pale yellow oil. TLC (dichloromethane/methanol 95:5 + 0.5% aq. NH_3_); *R_f_* = 0.44. ^1^H NMR (CDCl_3_): δ 7.18 (d, *J* = 8.9 Hz, 2H), 6.97–6.80 (m, 6H), 4.41–4.25 (m, 2H), 4.12–4.00 (m, 3H), 3.11–3.05 (m, 2H), 3.03–2.92 (m, 5H). A solution of the amine (61 mg, 0.16 mmol) in 2 mL of 2.0 M hydrogen chloride solution in diethyl ether was stirred overnight. The reaction mixture was diluted with ethyl ether, and the solid was filtered and washed with cooled ethyl ether to obtain the desired product **6** as a pale yellow solid (42.4 mg, 0.10 mmol). Mp 200 °C (decomposition). HRMS: *m*/*z* 379.1319 [M + H]^+^. ^1^H NMR (CD_3_OD): δ 7.23 (d, *J* = 8.9 Hz, 2H), 7.06–6.83 (m, 6H), 4.63 (m, 1H), 4.40–4.29 (m, 3H), 4.06 (dd, *J* = 11.6, 6.4 Hz, 1H), 3.58 (t, *J* = 5.0 Hz, 2H), 3.55–3.34 (m, 2H), 2.89 (s, 3H). ^13^C NMR (CD_3_OD): δ 155.55, 142.93, 141.59, 131.74, 123.56, 121.90, 121.67, 117.17, 117.01, 115.08, 69.09, 64.77, 63.02, 37.39.

#### [((2-(4-Ethylsulfonylamidephenoxy)ethyl)amino)-methyl]-1,4-benzodioxane Hydrochloride (**7**)

*N*-(4-(2-Aminoethoxy)phenyl)ethanesulfonamide **30a** (525 mg, 2.15 mmol) and 2-mesyloxymethyl-1,4-benzodioxane (477 mg, 1.95 mmol) were dissolved in 20 mL of 2-propanol. Triethylamine (0.27 mL, 1.95 mmol) was added to the solution, and the resulting mixture was refluxed, under stirring, until TLC indicated the disappearance of the starting material. Afterward, the solvent was evaporated in vacuo, and the crude was dissolved in dichloromethane to remove by filtration the unreacted *N*-(4-(2-aminoethoxy)phenyl)ethanesulfonamide. The organic phase was washed with a 10% aqueous solution of NaHCO_3_ and water and dried over anhydrous sodium sulfate, filtered, and evaporated in vacuo, affording an orangish oil. Flash chromatography (dichloromethane/methanol 95:5 + 0.5% aq. NH_3_) was performed to obtain 114.0 mg (0.29 mmol) of the pure [((2-(4-ethylsulfonylamidephenoxy)ethyl)amino)-methyl]-1,4-benzodioxane as a pale yellow oil. TLC (dichloromethane/methanol 90:10 + 1% aq. NH_3_); *R_f_* = 0.42. ^1^H NMR (CDCl_3_): δ 7.17 (d, *J* = 8.9 Hz, 2H), 6.95–6.76 (m, 6H), 6.40 (s, 1H), 4.37–4.25 (m, 2H), 4.11–3.98 (m, 3H), 3.17–2.87 (m, 6H), 1.37 (t, *J* = 7.4 Hz, 3H). A solution of the amine (114 mg, 0.29 mmol) in 4 mL of 2.0 M hydrogen chloride solution in diethyl ether was stirred overnight. The reaction mixture was diluted with ethyl ether, and the solid was filtered and washed with cooled ethyl ether to obtain the desired product **7** as a pale yellow solid (21.6 mg, 0.05 mmol). Mp 192 °C (decomposition). HRMS: *m*/*z* 393.1473 [M + H]^+^. ^1^H NMR (CD_3_OD): δ 7.21 (d, *J* = 8.9 Hz, 2H), 7.02–6.81 (m, 6H), 4.62 (ddt, *J* = 9.5, 6.5, 2.8 Hz), 4.37–4.28 (m, 3H), 4.05 (dd, *J* = 11.6, 6.5 Hz, 1H), 3.58 (t, *J* = 5.0 Hz, 2H), 3.53–3.35 (m, 2H), 3.01 (q, *J* = 7.4 Hz, 2H), 1.30 (t, *J* = 7.4 Hz, 3H). ^13^C NMR (CD_3_OD): δ 155.26, 142.93, 141.59, 131.79, 123.00, 121.90, 121.66, 117.16, 117.01, 115.07, 69.08, 64.76, 63.01, 44.69, 6.96.

#### [((2-(4-Isobutylsulfanilaminophenoxy)ethyl)amino)-methyl]-1,4-benzodioxane Hydrochloride (**8**)

*N*-(4-(2-Aminoethoxy)phenyl)-2-methylpropan-1-sulfonamide **30b** (658 mg, 2.42 mmol) and 2-mesyloxymethyl-1,4-benzodioxane (445 mg, 1.82 mmol) were dissolved in 20 mL of 2-propanol. Triethylamine (0.25 mL, 1.82 mmol) was added to the solution, and the resulting mixture was refluxed, under stirring, until TLC indicated the disappearance of the starting material. Afterward, the solvent was evaporated in vacuo, and the crude was dissolved in dichloromethane to remove by filtration the unreacted *N*-(4-(2-aminoethoxy)phenyl)-2-methylpropan-1-sulfonamide. The organic phase was washed with a 10% aqueous solution of NaHCO_3_ and water and dried over anhydrous sodium sulfate, filtered, and evaporated in vacuo, affording an orangish oil. Flash chromatography (dichloromethane/methanol 97:3 + 0.5% aq. NH_3_) was performed to obtain 150 mg (0.36 mmol) of the pure [((2-(4-isobutylsulfanilaminophenoxy)ethyl)amino)-methyl]-1,4-benzodioxane as a pale yellow oil. TLC (dichloromethane/methanol 90:10 + 0.5% aq. NH_3_); *R_f_* = 0.44. ^1^H NMR (CDCl_3_): δ 7.16 (d, *J* = 8.9 Hz, 2H), 6.95–6.8 (m, 6H), 6.38 (s, 1H), δ 4.35–4.24 (m, 2H), 4.16–4.01 (m, 3H), 3.07 (t, *J* = 5.2 Hz, 2H), 3.02–2.84 (m, 4H), 2.28 (m, 1H), 1.08 (d, *J* = 6.8 Hz, 6H). A solution of the amine (150 mg, 0.36 mmol) in 4.5 mL of 2.0 M hydrogen chloride solution in diethyl ether was stirred overnight. The reaction mixture was diluted with ethyl ether, and the solid was filtered and washed with cooled ethyl ether to obtain the desired product **8** as a pale yellow solid (165 mg, 0.36 mmol). Mp 206 °C (decomposition). HRMS: *m*/*z* 421.1785 [M + H]^+^. ^1^H NMR (CD_3_OD): δ 7.21 (d, *J* = 8.9 Hz, 2H), 7.05–6.83 (m, 6H), δ 4.64 (ddt, *J* = 9.4, 6.5, 3.0 Hz, 1H), 4.37–4.27 (m, 3H), 4.06 (dd, *J* = 11.6, 6.5 Hz, 1H), 3.59 (t, *J* = 5.0 Hz, 2H), 3.56–3.37 (m, 2H), 2.89 (d, *J* = 6.4 Hz, 2H), 2.20 (m, 1H), 1.04 (d, *J* = 6.7 Hz, 6H). ^13^C NMR (CD_3_OD): δ 155.30, 142.93, 141.62, 131.75, 125.90, 122.98, 121.86, 121.65, 117.20, 116.99, 115.10, 69.09, 64.79, 63.03, 57.98, 24.53, 21.34.

#### 4-(3-Azido-3-(1,4-benzodioxan)propoxy)benzenesulfonamide (**9**)

*N*,*N*-Dimethylaminomethylene-4-hydroxybenzenesulfonamide **40** (18 mg, 0.077 mmol) was treated with 12 mg of K_2_CO_3_ (0.080 mmol) in 0.5 mL of dimethylformamide, and the suspension was stirred at room temperature for 30 min under a nitrogen atmosphere. Then, a solution of 1-azido-1-(1,4-benzodioxan)-propylmethanesulfonate **38** (22 mg, 0.07 mmol) in dimethylformamide (1 mL) was added dropwise and the reaction was stirred at 40 °C under stirring until TLC indicated the disappearance of the starting material. The reaction mixture was quenched with cold water, and the precipitate formed was collected by filtration and dried under vacuo to afford 25 mg of *N*,*N*-dimethylaminomethylene-4-(3-azido-3-(1,4-benzodioxan)-propoxy)benzenesulfonamide (0.056 mmol). TLC (dichloromethane/methanol 98:2); *R_f_* = 0.56. Mp 178 °C. ^1^H NMR (CDCl_3_): δ 8.12 (s, 1H), 7.83 (d, *J* = 8.8 Hz, 2H), 6.99–6.82 (m, 6H), 4.25 (m, 5H), 3.83 (m, 1H), 3.12 (s, 2H), 3.01 (s, 2H), 2.20 (m, 2H). Hydrazine monohydrate (0.52 μL, 1.09 mmol) was added at room temperature to a stirred mixture of 52 mg of the *N*-sulfonylformamidine compound (0.109 mmol) in methanol (1.5 mL). The reaction mixture was stirred under stirring until TLC indicated the disappearance of the starting material. Then, the solvent was removed to obtain 45 mg of the pure product **9** (0.109 mmol) as a pale yellow solid. TLC (dichloromethane/methanol 98:2); *R_f_* = 0.44. Mp 107 °C. HRMS: *m*/*z* 389.0923 [M + H]^−^. ^1^H NMR (CD_3_OD): δ 7.93–7.79 (d, *J* = 9.0 Hz, 2H), 7.15–7.05 (d, *J* = 9.0 Hz, 2 H), 7.15–7.05 (m, 4H), 4.61–4.25 (m, 5H), 3.91–3.67 (m, 1H), 2.51–2.35 (m, 1H), 2.37–2.22 (m,1H). ^13^C NMR (CD_3_OD): δ 161.52, 143.27, 142.63, 135.63, 127.85, 121.35, 121.29, 116.87, 116.79, 116.69, 114.23, 75.63, 65.07, 64.46, 58.35, 29.31.

#### 3-Methoxy-4-(3-azido-3-(1,4-benzodioxan)propoxy)benzenesulfonamide (**10**)

*N*,*N*-Dimethylaminomethylene-3-methoxy-4-hydroxybenzenesulfonamide **43** (45 mg, 0.17 mmol) was treated with 25 mg of K_2_CO_3_ (0.18 mmol) in 0.5 mL of dimethylformamide, and the suspension was stirred at room temperature for 30 min under a nitrogen atmosphere. Then, a solution of 1-azido-1-(1,4-benzodioxan)-propylmethanesulfonate **38** (50 mg, 0.16 mmol) in dimethylformamide (1.5 mL) was added dropwise and the reaction was stirred at 40 °C until TLC indicated the disappearance of the starting material The reaction mixture was quenched with cold water, and the white precipitate formed was collected by filtration and dried under vacuo to afford 52 mg (0.11 mmol) of the desired compound *N*,*N*-dimethylaminomethylene-3-methoxy-4-(3-azido-3-(1,4-benzodioxan)propoxy)benzenesulfonamide. TLC (dichloromethane/methanol 98:2); *R_f_* = 0.51. Mp 180 °C. ^1^H NMR (CD_3_OD): δ 8.16 (s, 1H), 7.11 (s, 2H), 6.96–6.70 (m, 4H), 4.43 3.97 (m, 5H), 3.87–3.74 (m, 1H), 3.83–3.68 (m, 6H), 3.17 (s, 3H), 3.00 (s, 3H), 2.28–2.02 (m, 1H), 2.00–1.77 (m, 1H). Hydrazine monohydrate (0.17 mL, 3.58 mmol) was added at room temperature to a stirred mixture of the *N*-sulfonylformamidine compound (181 mg, 0.36 mmol) in methanol (4 mL). The reaction mixture was stirred until TLC indicated the disappearance of the starting material. Then, the solvent was removed to obtain 161 mg of the pure product **10** (0.35 mmol) as a pale yellow solid. TLC (dichloromethane/methanol 98:2); *R_f_* = 0.37. Mp 114 °C. HRMS: *m*/*z* 419.1027 [M + H]^−. 1^H NMR (CD_3_OD): δ 7.48 (dd, *J* = 8.4, 2.2 Hz, 1H), 7.42 (d, *J* = 2.1 Hz, 1H), 7.08 (dd, *J* = 8.4, 3.5 Hz, 1H), 6.90–6.76 (m, 4H), 4.43–4.32 (m, 1H), 4.31–4.14 (m, 4H), 3.97–3.87 (m, 1H), 3.81 (s, *J* = 8.7 Hz, 3H), 2.36–2.21 (m, 1H), 2.20–2.07 (m, 1H). ^13^C NMR (CD_3_OD): δ 153.23, 143.39, 143.24, 138.90, 121.07, 116.90, 116.57, 103.23, 75.55,70.11, 65.56, 55.43, 49.06, 32.46.

#### 3,5-Dimethoxy-4-(3-azido-3-(1,4-benzodioxan)propoxy)benzenesulfonamide (**11**)

*N*,*N*-Dimethylaminomethylene-3,5-dimethoxy-4-hydroxybenzenesulfonamide **46** (125 mg, 0.43 mmol) was treated with 62 mg of K_2_CO_3_ (0.45 mmol) in 1 mL of dimethylformamide, and the suspension was stirred at room temperature for 30 min under a nitrogen atmosphere. Then, a solution of 1-azido-1-(1,4-benzodioxan)-propylmethanesulfonate **38** (122 mg, 0.39 mmol) in dimethylformamide (3 mL) was added dropwise and the reaction was stirred at 40 °C until TLC indicated the disappearance of the starting material. The reaction mixture was quenched with cold water, and the white precipitate formed was collected by filtration and dried under vacuo to afford 163 mg (0.32 mmol) of the pure product *N*,*N*-dimethylaminomethylene-3,5-dimethoxy-4-(3-azido-3-(1,4-benzodioxan)propoxy)benzenesulfonamide. TLC (dichloromethane/methanol 95:5); *R_f_* = 0.88. Mp 223 °C (degradation). ^1^H NMR (CD_3_OD): δ 8.16 (s, 1H), 7.11 (s, 2H), 6.96–6.70 (m, 4H), 4.43 3.97 (m, 5H), 3.87–3.74 (m, 1H), 3.83–3.68 (m, 6H), 3.17 (s, 3H), 3.00 (s, 3H), 2.28–2.02 (m, 1H), 2.00–1.77 (m, 1H). Hydrazine hydrate (0.17 mL, 3.58 mmol) was added at room temperature to a stirred mixture of 159 mg of the *N*-sulfonylformamidine compound (0.31 mmol) in methanol (4 mL). The reaction mixture was stirred until TLC indicated the disappearance of the starting material. Then, the mixture was evaporated to dryness under reduced pressure to obtain 161 mg of the pure product **11** (0.35 mmol) as a white solid. TLC (dichloromethane/methanol 98:2); *R_f_* = 0.48. Mp = 128 °C. HRMS: *m*/*z* 449.1142 [M + H]^−^. ^1^H NMR (CD_3_OD): δ 7.20 (s, 2H), 7.00–6.71 (m, 4H), 4.41–4.00 (m, 5H), 3.84 (m, 6H), 3.77–3.70 (m, 1H), 2.25–2.00 (m, 1H), 2.02–1.65 (m, 1H). 13C NMR (CD_3_OD): δ 153.23, 143.39, 143.24, 138.90, 121.07, 116.90, 116.57, 103.23, 75.55, 70.11, 65.56, 55.43, 49.06, 32.46.

#### 4-(3-Amino-3-(1,4-benzodioxan)propoxy)benzenesulfonamide (**12**)

To a solution of 178 mg of 4-(3-azido-3-(1,4-benzodioxan)propoxy)benzenesulfonamide **9** (0.45 mmol) in methanol (4 mL) was added 200 μL of hydrazine hydrate (4.50 mmol) and PdO (55 mg, 0.45 mmol). The mixture was then heated at reflux until TLC indicated the disappearance of the starting material. After that, the solvent was evaporated under reduced pressure to obtain 115 mg of the pure product **12** (0.31 mmol) as a white solid. TLC (dichloromethane/methanol 98:2 + 1% NH_3_); *R_f_* = 0.20. Mp 172 °C. HRMS: *m*/*z* 365.1166 [M + H]^+^. ^1^H NMR (CD_3_OD): δ 7.85 (d, *J* = 8.9 Hz, 2H), 7.10 (d, *J* = 8.9 Hz, 2H), 7-04–6.77 (m, 4H), 4.59–4.38 (m, 2H), 4.38–4.23 (m, 2H), 4.23–4.03 (m, 1H), 3.86–3.65 (m, 1H), 2.49–2.36 (m, 1H), 2.36–2.18 (m, 1H). ^13^C NMR (CD_3_OD): δ 161.02, 143.08, 141.94, 127.85, 121.93, 121.57, 117.10, 116.88, 114.27, 72.39, 64.42, 63.91, 49.34, 28.78.

#### 3-Methoxy-4-(3-amino-3-(1,4-benzodioxan)propoxy)benzenesulfonamide (**13**)

To a solution of 62 mg of 3-methoxy-4-(3-azido-3-(1,4-benzodioxan)propoxy)benzenesulfonamide **10** (0.13 mmol) in methanol (1.5 mL) was added 63 μL of hydrazine hydrate (1.3 mmol) and PdO (16 mg, 0.13 mmol). The mixture was then heated at reflux until TLC indicated the disappearance of the starting material. After that, the solvent was evaporated under reduced pressure to obtain 41 mg of the pure product **13** (0.10 mmol) as a pinkish solid. TLC (dichloromethane/methanol 98:2 + 1% NH_3_); *R_f_* = 0.12. Mp 125 °C. HRMS: *m*/*z* 395.1271 [M + H]^+^. ^1^H NMR (CD_3_OD): 7.48 (dd, *J* = 8.4, 2.2 Hz, 1H), 7.42 (d, *J* = 2.2 Hz, 1H), 7.08 (d, *J* = 8.4 Hz, 1H), 6.87–6.75 (m, 4H), 4.40 (d, *J* = 9.2 Hz, 1H), 4.33–4.19 (m, 2H), 4.16–4.03 (m, 2H), 3.82 (s, 3H), 2.25–2.10 (m, 1H), 2.09–1.91 (m, 1H). ^13^C NMR (CD_3_OD): δ 151.77, 148.26, 147.33, 139.67, 121.58, 120.84, 117.85, 117.46, 115.91, 110.73, 79.54, 67.60, 66.98, 56.78, 48.64, 32.48.

#### 3,5-Dimethoxy-4-(3-amino-3-(1,4-benzodioxan)propoxy)benzenesulfonamide (**14**)

To a solution of 161 mg of 3,5-dimethoxy-4-(3-azido-3-(1,4-benzodioxan)propoxy)benzenesulfonamide **11** (0.35 mmol) in methanol (4 mL) was added 0.17 mL of hydrazine hydrate (3.5 mmol) and PdO (44 mg, 0.36 mmol). The mixture was then heated at reflux until the starting material was consumed (TLC monitoring). After that, the solvent was evaporated under reduced pressure to obtain 124 mg of the pure product **14** (0.29 mmol) as a pinkish solid. TLC (dichloromethane/methanol 98:2 + 1% NH_3_); *R_f_* = 0.20. Mp *=* 138 °C. HRMS: *m*/*z* 425.1371 [M + H]^+. 1^H NMR (CD_3_OD): δ 7.22 (s, 2H), 6.92–6.75 (m, 4H), 4.50–4.29 (m, 1H), 4.29–4.01 (m, 4H), 3.88 (s, 6H), 3.49–3.36 (m, 1H), 2.28–2.01 (m, 1H), 2.02–1.77 (m, 1H). ^13^C NMR (CD_3_OD): δ 153.23, 143.39, 143.23, 138.90, 121.07, 116.90, 116.56, 103.21, 76.04, 75.54, 70.10, 65.56, 55.43, 32.46.

#### 2-Chloroethoxybenzene (**15**)

TBAB (685.0 g, 2.13 mmol) was added to a stirred solution of 2.00 g of phenol (21.25 mmol) in dichloromethane (40 mL) and 2.5 N NaOH (85 mL). After 30 min, 1-bromo-2-chloroethane (7.1 mL, 85.90 mmol) was added dropwise and the resulting mixture was stirred for 48 h at room temperature. Afterward, the phases were separated and the organic one was sequentially washed with brine, dried over anhydrous sodium sulfate, filtered, and concentrated under reduced pressure, affording a crude that was purified through silica gel flash chromatography (cyclohexane/ethyl acetate 8:2). The pure product **15** was isolated as a colorless oil (1.58 g, 10.09 mmol). TLC (cyclohexane/ethyl acetate 7:3); *R_f_* = 0.6. ^1^H NMR (CDCl_3_): δ 7.36–7.24 (m, 2H), 7.02–6.89 (m, 3H), 4.24 (t, *J* = 5.9 Hz, 2H), 3.82 (t, *J* = 5.9 Hz, 2H).

#### 4-(2-Chloroethoxy)benzenesulfonyl Chloride (**16**)

2-Chloroethoxybenzene **17** (3.00 g, 19.16 mmol) was dissolved in 50 mL of dichloromethane, and after the solution was cooled to −10 °C, chlorosulfonic acid (2.55 mL, 38.32 mmol) was added dropwise. The resulting reaction was stirred at −10 °C for 2 h and another hour at room temperature. Afterward, the reaction mixture was quenched with ice and extracted with dichloromethane, and the organic phase was dried over anhydrous sodium sulfate and filtered. The solvent was evaporated in vacuo, providing 3.44 g (13.48 mmol) of the desired product **17** as a pink oil. ^1^H NMR (CDCl_3_): δ 7.99 (d, *J* = 9.1 Hz, 2H), 7.07 (d, *J* = 9.1 Hz, 2H), 4.34 (t, *J* = 5.7 Hz, 2H), 3.86 (t, *J* = 5.7 Hz, 2H).

#### 4-(2-Chloroethoxy)benzenesulfonamide (**17a**)

Gaseous ammonia was bubbled in an ice-cooled solution of 4-(2-chloroethoxy)benzenesulfonyl chloride **16** (850 mg, 3.33 mmol) in 20 mL of dichloromethane until saturation. The reaction mixture was stirred for 45 min. Afterward, ammonium chloride was removed by filtration, and dichloromethane was evaporated under vacuum to obtain the pure product **17a** as a white solid (730 mg, 3.12 mmol). Mp 115 °C. ^1^H NMR (CDCl_3_): δ 7.88 (d, *J* = 9.0 Hz, 2H), 7.00 (d, *J* = 9.0 Hz, 2H), 4.75 (s, 2H), 4.29 (t, *J* = 5.8 Hz, 2H), 3.84 (t, *J* = 5.8 Hz, 2H).

#### 4-(2-Azidoethoxy)benzenesulfonamide (**18a**)

Sodium azide (2.03 g, 31.22 mmol) and KI (52 mg, 0.31 mmol) were added to a solution of 4-(2-chloroethoxy)benzenesulfonamide **17a** (736 mg, 3.12 mmol) in dimethylformamide (10 mL) and water (3 mL). Then, the mixture was refluxed, under stirring, until TLC indicated the disappearance of the starting material. Afterward, the mixture was diluted with water and extracted with diethyl ether. The organic phase was dried over anhydrous sodium sulfate, filtered, and evaporated in vacuo, affording 685 mg (2.83 mmol) of the pure product **18a** as a colorless oil. TLC (dichloromethane/methanol 95:5); *R_f_* = 0.5. ^1^H NMR (CDCl_3_): δ 7.88 (d, *J* = 9.0 Hz, 2H), 7.01 (d, *J* = 9.0 Hz, 2H), 4.90 (s, 2H), 4.21 (t, *J* = 5.9 Hz, 2H), 3.65 (t, *J* = 5.9 Hz, 2H).

#### 4-(2-Aminoethoxy)benzenesulfonamide (**19a**)

Palladium(II) oxide (34.28 mg, 0.28 mmol) and hydrazine hydrate (1.38 mL, 28.30 mmol) were added to a solution of 4-(2-azidoethoxy)benzenesulfonamide **18a** (685 mg, 2.83 mmol) in methanol (25 mL). Then, the mixture was refluxed, under stirring, until TLC indicated the disappearance of the starting material. Afterward, the catalyst was removed by filtration, and methanol was evaporated in vacuo. The resulting crude was dissolved in ethyl acetate, and the organic phase was extracted with a 10% aqueous solution of HCl. After being basified to pH 10 with a 10 M solution of KOH, the aqueous layer was further extracted with ethyl acetate. The organic phase was dried over anhydrous sodium sulfate, filtered, and evaporated in vacuo, affording 410 mg (1.91 mmol) of the pure product **20a** as a white solid. TLC (dichloromethane/methanol 90:10 + 1% aq. NH_3_); *R_f_* = 0.2. Mp 139 °C. ^1^H NMR (CD_3_OD): δ 7.83 (d, *J* = 9.0 Hz, 2H), 7.07 (d, *J* = 9.0 Hz, 2H), 4.08 (t, *J* = 5.3 Hz, 2H), 3.03 (t, *J* = 5.3 Hz, 2H).

#### 4-(2-Chloroethoxy)-*N*-methylbenzenesulfonamide (**17b**)

Methylamine solution (2.0 M) in tetrahydrofuran (10 mL, 20.00 mmol) was added to an ice-cooled solution of 4-(2-chloroethoxy)benzenesulfonyl chloride **16** (500 mg, 1.96 mmol) in tetrahydrofuran (2 mL). Afterward, the solid was removed by filtration, and the solvent was evaporated under vacuum to obtain the pure product **17b** as a yellow oil (449 mg, 1.80 mmol). ^1^H NMR (CDCl_3_): δ 7.81 (d, *J* = 8.8 Hz, 2H), 7.01 (d, *J* = 8.8 Hz, 2H), 4.29 (t, *J* = 5.8 Hz, 2H), 3.84 (t, *J* = 5.8 Hz, 2H), 2.65 (s, 3H).

#### 4-(2-Azidoethoxy)-*N*-methylbenzenesulfonamide (**18b**)

Sodium azide (1.14 g, 17.53 mmol) and KI (30 mg, 0.18 mmol) were added to a solution of 4-(2-chloroethoxy)-*N*-methylbenzenesulfonamide **17b** (440 mg, 1.76 mmol) in dimethylformamide (6 mL) and water (2 mL). Then, the mixture was refluxed, under stirring, until TLC indicated the disappearance of the starting material. Afterward, the mixture was diluted with water and extracted with diethyl ether. The organic phase was dried over anhydrous sodium sulfate, filtered, and evaporated in vacuo, affording 451 mg (1.76 mmol) of the pure product **18b** as a pale yellow oil. TLC (dichloromethane/methanol 95:5); *R_f_* = 0.55. ^1^H NMR (CDCl_3_): δ 7.78 (d, *J* = 8.9 Hz, 2H), 6.98 (d, *J* = 8.9 Hz, 2H), 4.19 (t, *J* = 6.2 Hz, 2H), 3.62 (t, *J* = 6.2 Hz, 2H), 2.61 (s, 3H).

#### 4-(2-Aminoethoxy)-*N*-methylbenzenesulfonamide (**19b**)

Palladium(II) oxide (21.54 mg, 0.18 mmol) and hydrazine hydrate (0.86 mL, 17.60 mmol) were added to a solution of 4-(2-azidoethoxy)-*N*-methylbenzenesulfonamide **18b** (451 mg, 1.76 mmol) in methanol (6 mL). Then, the mixture was refluxed, under stirring, until TLC indicated the disappearance of the starting material. Afterward, the catalyst was removed by filtration, and methanol was evaporated in vacuo. The resulting crude was dissolved in ethyl acetate, and the organic phase was extracted with a 10% aqueous solution of HCl. After being basified to pH 10 with a 10 M solution of KOH, the aqueous layer was further extracted with ethyl acetate. The organic phase was dried over anhydrous sodium sulfate, filtered, and evaporated in vacuo, affording 170 mg (0.74 mmol) of the pure product **19b** as an orange oil. TLC (dichloromethane/methanol 90:10 + 1% aq. NH_3_); *R_f_* = 0.15. ^1^H NMR (CD_3_OD): δ 7.83 (d, *J* = 9.0 Hz, 2H), 7.07 (d, *J* = 9.0 Hz, 2H), 4.08 (t, *J* = 5.3 Hz, 2H), 3.03 (t, *J* = 5.3 Hz, 2H), 2.50 (s, 3H).

#### [((2-(4-Methylsulfonamidephenoxy)ethyl)amino)-methyl]-1,4-benzodioxane Hydrochloride (**2**)

4-(2-Aminoethoxy)-*N*-methylbenzenesulfonamide **19b** (170 mg, 0.74 mmol) and 2-mesyloxymethyl-1,4-benzodioxane (164 mg, 0.67 mmol) were dissolved in 7 mL of 2-propanol. Triethylamine (0.093 mL, 0.67 mmol) was added to the solution, and the resulting mixture was refluxed, under stirring, until TLC indicated the disappearance of the starting material. Afterward, the solvent was evaporated in vacuo, and the crude was dissolved in dichloromethane and washed with a 10% aqueous solution of NaHCO_3_ and water. The organic phase was dried over anhydrous sodium sulfate, filtered, and evaporated in vacuo, affording a yellow oil. Flash chromatography (dichloromethane/methanol 90:10 + 1% aq. NH_3_) was performed to obtain 66 mg (0.17 mmol) of the pure product as a light yellow oil. TLC (dichloromethane/methanol 95:5 + 1% aq. NH_3_); *R_f_* = 0.40. ^1^H NMR (CDCl_3_): δ 7.78 (d, *J* = 8.9 Hz, 2H), 6.98 (d, *J* = 8.9 Hz, 2H), 6.90–6.79 (m, 4H), 5.05 (s, 1H), 4.32–4.24 (m, 2H), 4.14 (t, *J* = 5.1 Hz, 2H), 4.04 (dd, *J* = 11.6, 7.6 Hz, 1H), 3.10 (t, *J* = 5.1 Hz, 2H), 2.98 (dd, *J* = 8.8, 5.7 Hz, 2H), 2.59 (s, 3H).

A solution of [((2-(4-methylsulfonamidephenoxy)ethyl)amino)-methyl]-1,4-benzodioxane (66 mg, 0.17 mmol) in 2 mL of 2.0 M hydrogen chloride solution in diethyl ether was stirred overnight. The reaction mixture was diluted with ethyl ether, and the solid was filtered and washed with cooled ethyl ether to obtain the desired product **2** as a pale yellow solid (20.24 mg, 0.05 mmol). Mp 161 °C. HRMS: *m*/*z* 379.1318 [M + H]^+^. ^1^H NMR (CD_3_OD): δ 7.80 (d, *J* = 8.9 Hz, 2H), 7.18 (d, *J* = 8.9 Hz, 2H), 6.99–6.83 (m, 4H), 4.64 (m, 1H), 4.41 (t, *J* = 4.9 Hz, 2H), 4.35 (dd, *J* = 11.6, 2.4 Hz, 1H), 4.06 (dd, *J* = 11.6, 6.4 Hz, 1H), 3.62 (t, *J* = 4.9 Hz, 2H), 3.58–3.36 (m, 2H), 2.50 (s, 3H). ^13^C NMR (CD_3_OD): δ 160.93, 142.94, 141.59, 132.03, 129.02, 121.90, 121.66, 117.17, 117.02, 114.60, 69.10, 64.77, 63.17, 27.77.

#### 4-(2-Chloroethoxy)-*N*-ethylbenzenesulfonamide (**17c**)

Ethylamine solution (2.0 M) in tetrahydrofuran (9 mL, 18.00 mmol) was added to an ice-cooled solution of 4-(2-chloroethoxy)benzenesulfonyl chloride **16** (458 mg, 1.8 mmol) in tetrahydrofuran (0.5 mL). Afterward, the solid was removed by filtration, and the solvent was evaporated under vacuum to obtain the pure product **17c** as an orangish oil (474 mg, 1.8 mmol). ^1^H NMR (CDCl_3_): δ 7.81 (d, *J* = 8.9 Hz, 2H), 7.00 (d, *J* = 8.9 Hz, 2H), 4.29 (t, *J* = 5.8 Hz, 2H), 3.84 (t, *J* = 5.7 Hz, 2H), 2.99 (q, *J* = 7.2 Hz, 2H), 1.10 (t, *J* = 7.2 Hz, 3H).

#### 4-(2-Azidoethoxy)-*N*-ethylbenzenesulfonamide (**18c**)

Sodium azide (1.17 g, 18.00 mmol) and KI (30 mg, 0.18 mmol) were added to a solution of 4-(2-chloroethoxy)-*N*-methylbenzenesulfonamide **17c** (474 mg, 1.80 mmol) in dimethylformamide (5 mL) and water (2 mL). Then, the mixture was refluxed, under stirring, until TLC indicated the disappearance of the starting material. Afterward, the mixture was diluted with water and extracted with diethyl ether. The organic phase was dried over anhydrous sodium sulfate, filtered, and evaporated in vacuo, affording 486 mg (1.80 mmol) of the pure product **18c** as a pale yellow oil. TLC (dichloromethane/methanol 99:1); *R_f_* = 0.32. ^1^H NMR (CDCl_3_): δ 7.81 (d, *J* = 8.7 Hz, 2H), 7.00 (d, *J* = 8.7 Hz, 2H), 4.20 (t, *J* = 6.2 Hz, 2H), 3.64 (t, *J* = 6.2 Hz, 2H), 2.95 (q, *J* = 7.1 Hz, 2H), 1.10 (t, *J* = 7.1 Hz, 3H).

#### 4-(2-Aminoethoxy)-*N*-ethylbenzenesulfonamide (**19c**)

Palladium(II) oxide (22.04 mg, 0.18 mmol) and hydrazine hydrate (0.88 mL, 18.00 mmol) were added to a solution of 4-(2-azidoethoxy)-*N*-ethylbenzenesulfonamide **18c** (486 mg, 1.80 mmol) in methanol (15 mL). Then, the mixture was refluxed, under stirring, until TLC indicated the disappearance of the starting material. Afterward, the catalyst was removed by filtration, and methanol was evaporated in vacuo. The resulting crude was dissolved in ethyl acetate, and the organic phase was extracted with a 10% aqueous solution of HCl. After being basified to pH 10 with a 10 M solution of KOH, the aqueous layer was further extracted with ethyl acetate. The organic phase was dried over anhydrous sodium sulfate, filtered, and evaporated in vacuo, affording 428 mg (1.75 mmol) of the pure product **19c** as an orange oil. TLC (dichloromethane/methanol 95:5 + 1% aq. NH_3_); *R_f_* = 0.22. ^1^H NMR (CD_3_OD): δ 7.76 (d, *J* = 8.9 Hz, 2H), 7.09 (d, *J* = 8.9 Hz, 2H), 4.08 (t, *J* = 5.3 Hz, 2H), 3.02 (t, *J* = 5.3 Hz, 2H), 2.85 (q, *J* = 7.1 Hz, 2H), 1.04 (t, *J* = 7.1 Hz, 3H).

#### [((2-(4-Ethylsulfonamidephenoxy)ethyl)amino)-methyl]-1,4-benzodioxane Hydrochloride (**3**)

4-(2-Aminoethoxy)-*N*-ethylbenzenesulfonamide **19c** (428 mg, 1.75 mmol) and 2-mesyloxymethyl-1,4-benzodioxane (389 mg, 1.59 mmol) were dissolved in 18 mL of 2-propanol. Triethylamine (0.22 mL, 1.59 mmol) was added to the solution, and the resulting mixture was refluxed, under stirring, until TLC indicated the disappearance of the starting material. Afterward, the solvent was evaporated in vacuo, and the crude was dissolved in dichloromethane and washed with a 10% aqueous solution of NaHCO_3_ and water. The organic phase was dried over anhydrous sodium sulfate, filtered, and evaporated in vacuo, affording a yellow oil. Flash chromatography (dichloromethane/methanol 97:3 + 0.5% aq. NH_3_) was performed to obtain 124 mg (0.32 mmol) of the pure product as an orange oil. TLC (dichloromethane/methanol 95:5 + 0.5% aq. NH_3_); *R_f_* = 0.50. ^1^H NMR (CDCl_3_): δ 7.79 (d, *J* = 8.9 Hz, 2H), 6.98 (d, *J* = 8.9 Hz, 2H), 6.87 (m, 4H), 4.32–4.24 (m, 2H), 4.14 (t, *J* = 5.1 Hz, 2H), 4.04 (dd, *J* = 11.6, 7.6 Hz, 1H), 3.10 (t, *J* = 5.1 Hz, 2H), 3.06–2.90 (m, 4H), 1.10 (t, *J* = 7.2 Hz, 3H).

A solution of [((2-(4-ethylsulfonamidephenoxy)ethyl)amino)-methyl]-1,4-benzodioxane (124 mg, 0.32 mmol) in 4 mL of 2.0 M hydrogen chloride solution in diethyl ether was stirred overnight. The reaction mixture was diluted with ethyl ether, and the solid was filtered and washed with cooled ethyl ether to obtain the desired product **3** as a pale yellow solid (65.58 mg, 0.15 mmol). Mp 167 °C. HRMS: *m*/*z* 393.1473 [M + H]^+^. ^1^H NMR (CD_3_OD): δ 7.81 (d, *J* = 8.9 Hz, 2H), 7.17 (d, *J* = 8.9 Hz, 2H), 7.02–6.82 (m, 4H), 4.64 (m, 1H), 4.41 (t, *J* = 4.9 Hz, 2H), 4.35 (dd, *J* = 11.6, 2.4 Hz, 1H), 4.06 (dd, *J* = 11.6, 6.4 Hz, 1H), 3.62 (t, *J* = 4.9 Hz, 2H), 3.58–3.36 (m, 2H), 2.86 (q, *J* = 7.1 Hz, 2H), 1.05 (t, *J* = 7.1 Hz, 3H). ^13^C NMR (CD_3_OD): δ 160.89, 142.93, 141.62, 133.20, 128.83, 121.87, 121.66, 117.23, 116.99, 114.64, 69.11, 64.81, 63.20, 37.58, 13.81.

#### 4-(2-Chloroethoxy)-*N*,*N*-dimethylbenzenesulfonamide (**17d**)

Dimethylamine solution (2.0 M) in tetrahydrofuran (9 mL, 18.00 mmol) was added to an ice-cooled solution of 4-(2-chloroethoxy)benzenesulfonyl chloride **16** (458 mg,.1.80 mmol) in tetrahydrofuran (0.5 mL). Afterward, the solid was removed by filtration, and the solvent was evaporated under vacuum to obtain the pure product **17d** as a pale yellow oil (396 mg, 1.50 mmol). ^1^H NMR (CDCl_3_): δ 7.73 (d, *J* = 8.9 Hz, 2H), 7.02 (d, *J* = 8.9 Hz, 2H), 4.30 (t, *J* = 5.7 Hz, 2H), 3.85 (t, *J* = 5.7 Hz, 2H), 2.68 (s, 6H).

#### 4-(2-Azidoethoxy)-*N*,*N*-dimethylbenzenesulfonamide (**18d**)

Sodium azide (975 mg, 15.00 mmol) and KI (25 mg, 0.15 mmol) were added to a solution of 4-(2-chloroethoxy)-*N*,*N*-dimethylbenzenesulfonamide **17d** (396 mg, 1.50 mmol) in dimethylformamide (5 mL) and water (2 mL). Then, the mixture was refluxed, under stirring, until TLC indicated the disappearance of the starting material. Afterward, the mixture was diluted with water and extracted with diethyl ether. The organic phase was dried over anhydrous sodium sulfate, filtered, and evaporated in vacuo, affording 290 mg (1.07 mmol) of the pure product **18d** as a pale yellow oil. TLC (dichloromethane/methanol 99:1); *R_f_* = 0.70. ^1^H NMR (CDCl_3_): δ 7.72 (d, *J* = 8.9 Hz, 2H), 7.03 (d, *J* = 8.9 Hz, 2H), 4.21 (t, *J* = 5.7 Hz, 2H), 3.64 (t, *J* = 5.7 Hz, 2H), 2.68 (s, 6H).

#### 4-(2-Aminoethoxy)-*N*,*N*-dimethylbenzenesulfonamide (**19d**)

Palladium(II) oxide (13.47 mg, 0.11 mmol) and hydrazine hydrate (0.52 mL, 10.70 mmol) were added to a solution of 4-(2-azidoethoxy)-*N*,*N*-dimethylbenzenesulfonamide **18d** (290 mg, 1.07 mmol) in methanol (9 mL). Then, the mixture was refluxed, under stirring, until TLC indicated the disappearance of the starting material. Afterward, the catalyst was removed by filtration, and methanol was evaporated in vacuo. The resulting crude was dissolved in ethyl acetate, and the organic phase was extracted with a 10% aqueous solution of HCl. After being basified to pH 10 with a 10 M solution of KOH, the aqueous layer was further extracted with ethyl acetate. The organic phase was dried over anhydrous sodium sulfate, filtered, and evaporated in vacuo, affording 261 mg (1.07 mmol) of the pure product **19d** as a pale yellow solid. TLC (dichloromethane/methanol 90:10 + 1% aq. NH_3_); *R_f_* = 0.22. Mp 69 °C. ^1^H NMR (CD_3_OD): δ 7.71 (d, *J* = 8.9 Hz, 2H), 7.15 (d, *J* = 8.9 Hz, 2H), 4.10 (t, *J* = 5.3 Hz, 2H), 3.03 (t, *J* = 5.3 Hz, 2H), 2.64 (s, 6H).

#### 4-(2-Chloroethoxy)-*N*-isopropylbenzenesulfonamide (**17e**)

Isopropylamine (0.63 mL, 7.41 mmol) was added to an ice-cooled solution of 4-(2-chloroethoxy)benzenesulfonyl chloride **16** (860 mg, 3.37 mmol) in dichloromethane (20 mL). Afterward, the solid was removed by filtration, and the solvent was evaporated under vacuum to obtain the pure product **17e** as a pale yellow oil (936 mg, 3.37 mmol). ^1^H NMR (CDCl_3_): δ 7.82 (d, *J* = 8.9 Hz, 2H), 6.98 (d, *J* = 8.9 Hz, 2H), 4.28 (t, *J* = 5.7 Hz, 2H), 3.84 (t, *J* = 5.7 Hz, 2H), 3.42 (sep, *J* = 6.6 Hz, 1H), 1.06 (d, *J* = 6.6 Hz, 6H).

#### 4-(2-Azidoethoxy)-*N*-isopropylbenzenesulfonamide (**18e**)

Sodium azide (2.19 g, 33.7 mmol) and KI (56 mg, 0.34 mmol) were added to a solution of 4-(2-chloroethoxy)-*N*-isopropylbenzenesulfonamide **17e** (936 mg, 3.37 mmol) in dimethylformamide (10 mL) and water (2 mL). Then, the mixture was refluxed, under stirring, until TLC indicated the disappearance of the starting material. Afterward, the mixture was diluted with water and extracted with diethyl ether. The organic phase was dried over anhydrous sodium sulfate, filtered, and evaporated in vacuo, affording 841 mg (2.96 mmol) of the pure product **18e** as a pale yellow oil. TLC (dichloromethane/methanol 90:10 + 1% aq. NH_3_); *R_f_* = 0.13. ^1^H NMR (CDCl_3_): δ 7.82 (d, *J* = 8.9 Hz, 2H), 6.99 (d, *J* = 8.9 Hz, 2H), 4.19 (t, *J* = 5.7 Hz, 2H), 3.64 (t, *J* = 5.7 Hz, 2H), 3.43 (sep, *J* = 6.6 Hz, 1H), 1.07 (d, *J* = 6.6 Hz, 6H).

#### 4-(2-Aminoethoxy)-*N*-isopropylbenzenesulfonamide (**19e**)

Palladium(II) oxide (36.73 mg, 0.30 mmol) and hydrazine hydrate (1.44 mL, 29.60 mmol) were added to a solution of 4-(2-azidoethoxy)-*N*-isopropylbenzenesulfonamide **18e** (841 mg, 2.96 mmol) in methanol (25 mL). Then, the mixture was refluxed, under stirring, until TLC indicated the disappearance of the starting material. Afterward, the catalyst was removed by filtration, and methanol was evaporated in vacuo. The resulting crude was dissolved in ethyl acetate, and the organic phase was extracted with a 10% aqueous solution of HCl. After being basified to pH 10 with a 10 M solution of KOH, the aqueous layer was further extracted with ethyl acetate. The organic phase was dried over anhydrous sodium sulfate, filtered, and evaporated in vacuo, affording 718 mg (2.78 mmol) of the pure product **19e** as a pale yellow solid. TLC (dichloromethane/methanol 90:10 + 1% aq. NH_3_); *R_f_* = 0.13. Mp 119 °C ^1^H NMR (CD_3_OD): δ 7.78 (d, *J* = 8.9 Hz, 2H), 7.09 (d, *J* = 8.9 Hz, 2H), 4.08 (t, *J* = 5.3 Hz, 2H), 3.02 (t, *J* = 5.3 Hz, 2H), 3.30 (sep, *J* = 6.6 Hz, 1H), 1.00 (d, *J* = 6.6 Hz, 6H).

#### *tert*-Butyl (4-Hydroxyphenyl)carbamate (**20**)

*p-*Aminophenol (5.0 g, 45.82 mmol) was added to an ice-cooled solution of Boc_2_O (11.0 g, 50.40 mmol) in tetrahydrofuran (50 mL). The reaction mixture was stirred at 0 °C until TLC indicated the disappearance of the starting material. Afterward, the solvent was evaporated in vacuo and the crude was dissolved in ethyl acetate. The organic phase was washed with water and dried over anhydrous sodium sulfate, filtered, and evaporated in vacuo, affording 9.59 g (45.82 mmol) of the pure product **20** as a white solid. TLC (cyclohexane/ethyl acetate 7:3); *R_f_* = 0.86. Mp 145 °C. ^1^H NMR (CDCl_3_): δ 7.18 (d, *J* = 8.8 Hz, 2H), 6.74 (d, *J* = 8.8 Hz, 2H), 6.32 (s, 1H), 1.50 (s, 9H).

#### *tert*-Butyl (4-Hydroxyethoxy)phenyl)carbamate (**21**)

Ethylene carbonate (5.26 g, 59.73 mmol) and K_2_CO_3_ (8.26 g, 59.77 mmol) were added to a solution of *tert*-butyl (4-hydroxyphenyl)carbamate **20** (5.0 g, 23.89 mmol) in 50 mL of dimethylformamide. The resulting mixture was refluxed, under stirring, until TLC indicated the disappearance of the starting material. Afterward, the solvent was evaporated in vacuo and the crude was dissolved in ethyl acetate. The organic phase was washed with a 10% aqueous solution of NaOH and brine and then dried over anhydrous sodium sulfate, filtered, and evaporated in vacuo, affording a brownish oil. Flash chromatography (cyclohexane/ethyl acetate 8:2) was performed to obtain 3.27 g (12.93 mmol) of the pure product **21** as a brown oil. TLC (cyclohexane/ethyl acetate 6:4); *R_f_* = 0.80. ^1^H NMR (CDCl_3_): δ 7.18 (d, *J* = 8.9 Hz, 2H), 6.90 (d, *J* = 8.9 Hz, 2H), 4.05 (t, *J* = 4.5 Hz, 2H), 3.93 (t, *J* = 4.5 Hz, 2H), 2.75 (s, 1H), 1.50 (s, 9H).

#### 2-(4-Aminophenoxy)ethanol hydrochloride (**22**)

A solution of *tert*-butyl (4-hydroxyethoxy)phenyl)carbamate **21** (610 mg, 2.41 mmol) in 5 mL of 2.0 M hydrogen chloride solution in methanol was stirred at reflux until TLC indicated the disappearance of the starting material. Afterward, the solvent was evaporated in vacuo, affording 369 mg (1.94 mmol) of the desired product **22** as a white solid. TLC (cyclohexane/ethyl acetate 6:4); *R_f_* = 0. Mp 72 °C. ^1^H NMR (CD_3_OD): δ 7.33 (d, *J* = 9.1 Hz, 2H), 7.11 (d, *J* = 9.1 Hz, 2H), 4.05 (t, *J* = 4.5 Hz, 2H), 3.93 (t, *J* = 4.5 Hz, 2H), 2.75 (s, 1H).

#### (2-(4-Methylsulfonamide)phenoxy)ethyl Methanesulfonate (**23**)

Triethylamine (0.27 mL, 1.97 mmol) was added to a solution of 2-(4-aminophenoxy)ethanol **22** (369 mg, 1.94 mmol) in dichloromethane (10 mL), and the reaction solution was stirred for 20 min. Then, triethylamine (0.55 mL, 3.94 mmol) and mesyl chloride (0.31 mL, 3.94 mmol) were added dropwise to the ice-cooled reaction solution. The mixture was stirred at room temperature until TLC indicated the disappearance of the starting material. Afterward, the reaction was diluted with dichloromethane and the organic phase was washed with a 10% aqueous solution of HCl and then with brine. The organic phase was dried over anhydrous sodium sulfate and filtered, and the solvent was evaporated in vacuo, providing the pure product **23** as 402 mg of a white solid (1.30 mmol). TLC (dichloromethane/methanol 95:5 + 1% aq. NH_3_); *R_f_* = 0.70. Mp 137 °C. ^1^H NMR (CDCl_3_): δ 7.21 (d, *J* = 7.9 Hz, 2H), 6.91 (d, *J* = 7.9 Hz, 2H), 4.57 (t, *J* = 3.6 Hz, 2H), 4.24 (t, *J* = 3.6 Hz, 2H), 3.10 (s, 3H), 2.96 (s, 3H).

#### *N*-(4-(2-Azidoethoxy)phenyl)methanesulfonamide (**24**)

Sodium azide (832 mg, 12.80 mmol) was added to a solution of 2-(4-methylsulfonamide)phenoxy)ethyl methanesulfonate **23** (396 mg, 1.28 mmol) in dimethylformamide (26 mL) and water (9 mL). Then, the mixture was refluxed, under stirring, until TLC indicated the disappearance of the starting material. Afterward, the mixture was diluted with water and extracted with diethyl ether. The organic phase was dried over anhydrous sodium sulfate, filtered, and evaporated in vacuo, affording 219 mg (0.86 mmol) of the pure product **24** as an orangish oil. TLC (dichloromethane/methanol 99:1); *R_f_* = 0.80. ^1^H NMR (CDCl_3_): δ 7.22 (d, *J* = 8.9 Hz, 2H), 6.90 (d, *J* = 8.9 Hz, 2H), 4.13 (t, *J* = 5.0 Hz, 2H), 3.60 (t, *J* = 5.0 Hz, 2H), 2.91 (s, 3H).

#### *N*-(4-(2-Aminoethoxy)phenyl)methanesulfonamide (**25**)

Palladium(II) oxide (13.47 mg, 0.11 mmol) and hydrazine hydrate (0.53 mL, 10.9 mmol) were added to a solution of *N*-(4-(2-azidoethoxy)phenyl) methanesulfonamide **24** (841 mg, 2.96 mmol) in methanol (9 mL). Then, the mixture was refluxed, under stirring, until TLC indicated the disappearance of the starting material. Afterward, the catalyst was removed by filtration, and methanol was evaporated in vacuo, affording 238 mg (1.03 mmol) of the pure product **25** as a pale yellow oil. TLC (dichloromethane/methanol 90:10 + 1% aq. NH_3_); *R_f_* = 0.09. ^1^H NMR (CD_3_OD): δ 7.19 (d, *J* = 8.9 Hz, 2H), 6.94 (d, *J* = 8.9 Hz, 2H), 4.00 (t, *J* = 5.3 Hz, 2H), 3.01 (t, *J* = 5.3 Hz, 2H), 2.87 (s, 3H).

#### 2-(4-((*tert*-Butoxycarbonyl)amino)phenoxy)ethyl Methanesulfonate (**26**)

*tert*-Butyl (4-hydroxyethoxy)phenyl)carbamate **21** (1.0 g, 3.95 mmol) was dissolved in dichloromethane (10 mL), and triethylamine (0.66 mL, 4.74 mmol) was added, and after the reaction mixture was cooled down to 0 °C, mesyl chloride (0.37 mL, 4.74 mmol) was added dropwise. The solution was stirred at room temperature until TLC indicated the disappearance of the starting material. The reaction mixture was washed with a 10% aqueous solution of HCl and then with brine. The organic phase was dried over anhydrous sodium sulfate and filtered, and the solvent was evaporated in vacuo, providing the pure product **26** as 1.31 g of a yellow oil (3.95 mmol). TLC (cyclohexane/ethyl acetate 6:4); *R_f_* = 0.8. Mp 125 °C. ^1^H NMR (CDCl_3_): δ 7.18 (d, *J* = 8.9 Hz, 2H), 6.90 (d, *J* = 8.9 Hz, 2H), 4.57 (t, *J* = 3.6 Hz, 2H), 4.24 (t, *J* = 3.6 Hz, 2H), 3.10 (s, 3H), 1.50 (s, 9H).

#### *tert*-Butyl (4-(2-Azidoethoxy)phenyl)carbamate (**27**)

Sodium azide (592 mg, 9.10 mmol) was added to a solution of 2-(4-((*tert*-butoxycarbonyl)amino)phenoxy)ethyl methanesulfonate **26** (300 mg, 0.91 mmol) in dimethylformamide (18 mL) and water (6 mL). Then, the mixture was refluxed, under stirring, until TLC indicated the disappearance of the starting material. Afterward, the mixture was diluted with water and extracted with diethyl ether. The organic phase was dried over anhydrous sodium sulfate, filtered, and evaporated in vacuo, affording 238 mg (0.85 mmol) of the pure product **27** as an orangish oil. TLC (dichloromethane/methanol 99:1); *R_f_* = 0.7. ^1^H NMR (CDCl_3_): δ 7.28 (d, *J* = 9.0 Hz, 2H), 6.86 (d, *J* = 9.0 Hz, 2H), 6.36 (s, 1H*), 4.12 (t, *J* = 5.0 Hz, 2H), 3.58 (t, *J* = 5.0 Hz, 2H), 1.51 (s, 9H).

#### 4-(2-Azidoethoxy)aniline Hydrochloride (**28**)

A solution of *tert*-butyl (4-(2-azidoethoxy)phenyl)carbamate **27** (768 mg, 2.76 mmol) in 5.3 mL of 2.0 M hydrogen chloride solution in methanol was stirred at reflux until TLC indicated the disappearance of the starting material. Afterward, the solvent was evaporated in vacuo, affording 491.83 mg (2.74 mmol) of the desired product **28** as a white solid. TLC (cyclohexane/ethyl acetate 6:4); *R_f_* = 0. Mp 72 °C. ^1^H NMR (CD_3_OD): δ 7.33 (d, *J* = 9.1 Hz, 2H), 7.11 (d, *J* = 9.1 Hz, 2H), 4.21 (t, *J* = 5.0 Hz, 2H), 3.62 (t, *J* = 5.0 Hz, 2H).

#### *N*-(4-(2-Azidoethoxy)phenyl)ethanesulfonamide (**29a**)

Triethylamine (0.36 mL, 2.56 mmol) was added to a solution of 4-(2-azidoethoxy)aniline **28** (369 mg, 1.94 mmol) in dichloromethane (10 mL), and the reaction solution was stirred for 20 min. Then, triethylamine (0.39 mL, 2.82 mmol) and ethanesulfonyl chloride (0.27 mL, 2.82 mmol) were added dropwise to the ice-cooled reaction solution. The mixture was stirred at room temperature until TLC indicated the disappearance of the starting material. Afterward, the reaction was diluted with dichloromethane and the organic phase was washed with a 10% aqueous solution of HCl and then with brine. The organic phase was dried over anhydrous sodium sulfate and filtered, and the solvent was evaporated in vacuo, providing the pure product **29a** as 590 mg of red oil (2.18 mmol). TLC (dichloromethane/methanol 97:3 + 1% aq. NH_3_); *R_f_* = 0.55. ^1^H NMR (CDCl_3_): δ 7.20 (d, *J* = 8.9 Hz, 2H), 6.89 (d, *J* = 8.9 Hz, 2H), 6.51 (s, 1H), 4.13 (t, *J* = 4.9 Hz, 2H), 3.59 (t, *J* = 4.9 Hz, 2H), 3.06 (q, *J* = 7.4 Hz, 2H), 1.37 (t, *J* = 7.4 Hz, 3H).

#### *N*-(4-(2-Aminoethoxy)phenyl)ethanesulfonamide (**30a**)

Palladium(II) oxide (26.93 mg, 0.22 mmol) and hydrazine hydrate (1.05 mL, 21.50 mmol) were added to a solution of *N*-(4-(2-azidoethoxy)phenyl)ethanesulfonamide **29a** (580 mg, 2.15 mmol) in methanol (18 mL). Then, the mixture was refluxed, under stirring, until TLC indicated the disappearance of the starting material. Afterward, the catalyst was removed by filtration, and methanol was evaporated in vacuo, affording 527 mg (2.15 mmol) of the pure product **30a** as a yellow oil. TLC (dichloromethane/methanol 90:10 + 1% aq. NH_3_); *R_f_* = 0.16. ^1^H NMR (CD_3_OD): δ 7.19 (d, *J* = 8.2 Hz, 2H), 6.93 (d, *J* = 8.2 Hz, 2H), 4.82 (s, 1H), 4.00 (t, *J* = 5.3 Hz, 2H), 3.05–2.95 (m, 4H), 1.30 (t, *J* = 7.4 Hz, 3H).

#### *N*-(4-(2-azidoethoxy)phenyl)-2-methylpropan-1-sulfonamide (**29b**)

Triethylamine (0.38 mL, 2.76 mmol) was added to a solution of 4-(2-azidoethoxy)aniline **28** (593 mg, 3.33 mmol) in dichloromethane (10 mL), and the reaction solution was stirred for 20 min. Then, triethylamine (0.42 mL, 3.04 mmol) and isobutanesulfonyl chloride (0.4 mL, 3.04 mmol) were added dropwise to the ice-cooled reaction solution. The mixture was stirred at room temperature until TLC indicated the disappearance of the starting material. Afterward, the reaction was diluted with dichloromethane and the organic phase was washed with 10% aqueous solution of HCl and then with brine. The organic phase was dried over anhydrous sodium sulfate and filtered, and the solvent was evaporated in vacuo, providing the pure product **29b** as 679 mg of a brown oil (2.28 mmol). TLC (dichloromethane/methanol 97:3 + 1% aq. NH_3_); *R_f_* = 0.67. ^1^H NMR (CDCl_3_): δ 7.19 (d, *J* = 8.9 Hz, 2H), 6.90 (d, *J* = 8.9 Hz, 2H), 6.50 (s, 1H), 4.14 (t, *J* = 4.9 Hz, 2H), 3.60 (t, *J* = 4.9 Hz, 2H), 2.92 (d, *J* = 6.7 Hz, 2H), 2.27 (m, 1H), 1.08 (d, *J* = 6.7 Hz, 6H).

#### *N*-(4-(2-Aminoethoxy)phenyl)-2-methylpropan-1-sulfonamide (**30b**)

Palladium(II) oxide (28.16 mg, 0.23 mmol) and hydrazine hydrate (1.11 mL, 22.80 mmol) were added to a solution of *N*-(4-(2-azidoethoxy)phenyl)ethanesulfonamide **29b** (679 mg, 2.28 mmol) in methanol (18 mL). Then, the mixture was refluxed, under stirring, until TLC indicated the disappearance of the starting material. Afterward, the catalyst was removed by filtration, and methanol was evaporated in vacuo, affording 621 mg (2.28 mmol) of the pure product **30b** as an orange oil. TLC (dichloromethane/methanol 90:10 + 0.5% aq. NH_3_); *R_f_* = 0.32. ^1^H NMR (CD_3_OD): δ 7.17 (d, *J* = 8.5 Hz, 2H), 6.93 (d, *J* = 8.5 Hz, 2H), 4.00 (t, *J* = 5.3 Hz, 2H), 3.02 (t, *J* = 5.3 Hz, 2H), 2.87 (d, *J* = 6.7 Hz, 2H), 2.20 (m, 1H), 1.04 (d, *J* = 6.7 Hz, 6H).

#### *N*-Methoxy-*N*-methyl-1,4-benzodioxan-2-carboxamide (**31**)

Thionyl chloride (3.26 mL, 44.74 mmol) was added dropwise to an ice-cooled solution of 1,4-benzodioxan-2-carboxylic acid (4.03 g, 22.37) in 40 mL of dichloromethane. The mixture was stirred at reflux until NMR indicated the disappearance of the starting material. Afterward, the solvent was evaporated in vacuo, and the crude was dissolved in 30 mL of dichloromethane and cooled down to 0 °C. Then, *N*-dimethylhydroxylamine hydrochloride (3.27 g, 33.56 mmol) was added in small amounts to the mixture and the reaction was stirred until TLC indicated the disappearance of the starting material. Afterward, the reaction was diluted with additional dichloromethane, washed three times with 10% aqueous solution of NaHCO_3_ and brine, dried over anhydrous sodium sulfate, and filtered. The solvent was then evaporated in vacuo, providing the pure product **31** as a yellow solid (4.69 g, 21.00 mmol). ^1^H NMR *O*-acyl chloride (CDCl_3_): δ 7.07–6.86 (m, 4H), 5.10 (m, 1H), 4.75 (dd, *J* = 11.8, 2.8 Hz, 1H), 4.34 (dd, *J* = 11.8, 2.8 Hz, 1H). TLC (cyclohexane/ethyl acetate 7:3 + 1% formic acid); *R_f_* = 0.5. Mp 87 °C. ^1^H NMR (CDCl_3_): δ 7.04–6.81 (m, 4H), 5.12–4.98 (m, 1H), 4.44 (dd, *J* = 11.4, 2.5 Hz, 1H), 4.26 (dd, *J* = 11.4, 7 Hz, 1H), 3.8 (s, 3H), 3.27 (s, 3H).

#### 1,4-Benzodioxan-2-carboxyaldehyde (**32**)

Under a nitrogen atmosphere, a solution of *N*-methoxy-*N*-methyl-1,4-benzodioxan-2-carboxamide **31** (2 g, 8.46 mmol) in 32 mL of tetrahydrofuran was added dropwise to a suspension of 425 mg of LiAlH_4_ (11.20 mmol) in tetrahydrofuran (8 mL) and allowed to cool down to −20 °C. The reaction mixture was stirred at the same temperature until TLC indicated the disappearance of the starting material. Afterward, the excess of LiAlH_4_ was quenched by slowly adding 10% aqueous solution of HCl and diluted with dichloromethane. After the separation of the phases, the aqueous layer was further extracted with dichloromethane. The reunited organic phases were washed with brine, dried over anhydrous sodium sulfate, and filtered. The solvent was evaporated in vacuo, providing the pure product **32** as a yellow oil (1.38 g, 8.46 mmol). TLC (cyclohexane/ethyl acetate 7:3); *R_f_* = 0.55. ^1^H NMR (CDCl_3_): δ 9.77 (s, 1H), 7.09–6.78 (m, 4H), 4.69–4.56 (m, 1H), 4.35 (dd, *J* = 4.5, 1.0 Hz, 2H). The neat product quickly degraded at room temperature but was stable for several days if dissolved in dichloromethane (ca. 20% w/v).

#### Ethyl 3-(1,4-Benzodioxan)-3-hydroxypropanoate (**33**)

Under a nitrogen atmosphere, 1.21 g of zinc (18.52 mmol) and 209 mg of TBDMSiCl (1.39 mmol) were suspended in 12 mL of tetrahydrofuran. The mixture was stirred at 50 °C for 40 min. Then, a solution of 1.52 g of 1,4-benzodioxan-2-carboxyaldehyde **32** (9.26 mmol) and 1.54 mL of ethyl bromoacetate (13.89 mmol) in 24 mL of tetrahydrofuran was added dropwise to the suspension. The reaction mixture was stirred at 50 °C until TLC indicated the disappearance of the starting material. Afterward, the solution was diluted with ethyl acetate and the organic layer was sequentially washed with 10% aqueous solution of HCl, 10% aqueous solution of NaHCO_3_, saturated solution of Na_2_SO_3_, and finally with brine. The organic phase was dried over anhydrous sodium sulfate and filtered, and the solvent was evaporated in vacuo, providing the pure product **33** as a pale yellow oil (2.08, 8.24 mmol). TLC (cyclohexane/ethyl acetate 8:2); *R_f_* = 0.53. ^1^H NMR (CDCl_3_): δ 6.99–6.71 (m, 4H), 4.42 (dd, *J* = 11.3, 2.2 Hz,1H), 4.27–4.00(m, 4H), 2.89 (dd, *J* = 17.0, 2.8 Hz, 1H), 2.62 (dd, *J* = 17.0, 8.6 Hz, 1 Hz), 1.29 (t, *J* = 7.2 Hz, 3H).

#### 1-(1,4-Benzodioxan)-1,3-propanediol (**34**)

Under a nitrogen atmosphere, a solution of ethyl 3-(1,4-benzodioxan)-3-hydroxypropanoate **33** (1.59 g, 6.30 mmol) in 20 mL of tetrahydrofuran was added dropwise to a suspension of 298 mg of LiAlH_4_ (7.87 mmol) in tetrahydrofuran (5 mL) and allowed to cool down to −10 °C. The reaction mixture was stirred at the same temperature until TLC indicated the disappearance of the starting material. Afterward, the excess of LiAlH_4_ was quenched by slowly adding a 10% aqueous solution of HCl and diluted with dichloromethane. After the separation of the phases, the aqueous layer was further extracted with dichloromethane. The reunited organic phases were washed with brine, dried over anhydrous sodium sulfate, and filtered. The solvent was evaporated in vacuo, affording a yellow oil. Flash chromatography (cyclohexane/ethyl acetate 1:1) was performed to obtain 630 mg (2.99 mmol) of the pure **34** as a white solid. TLC (cyclohexane/ethyl acetate 1:1); *R_f_* = 0.36, 0,34. Mp 61 °C. ^1^H NMR (CD_3_OD): δ 6.93–6.65 (m, 4H), 4.37 (dd, *J* = 11.3, 2.1 Hz, 1H), 4.07, (*J* = 11.3, 6.9 Hz, 1H), 4.00–3.82 (m, 2H), 3.76 (dd, *J* = 7.5, 5.7 Hz, 2H), 2.12–1.94 (m, 1H), 1.78–1.61 (m, 1H). ^1^H NMR (CD_3_OD): δ 6.91–6.74 (m, 4H), 4.32 (dd, *J* = 10.4, 1.1 Hz, 1H), 4.13–3.92 (m, 3H), 3.75 (t, *J* = 6.3, 2H), 1.93–1.81 (m, 2H).

#### 1-(1,4-Benzodioxan)-3-trityloxy-1-propanol (**35**)

Triethylamine (0.50 mL, 3.52 mmol) was added dropwise to an ice-cooled solution of 673 mg of 1-(1,4-benzodioxan)-1,3-propanediol **34** (3.20 mmol) and was dissolved in 10 mL of dichloromethane. The reaction mixture was warmed to room temperature, and then a solution of trityl chloride (981 g, 3.52 mmol) in 15 mL of dichloromethane was added dropwise to the solution. Then, the mixture was stirred, until TLC indicated the disappearance of the starting material. Afterward, the mixture was diluted with dichloromethane, and the organic phase was washed with brine, dried over anhydrous sodium sulfate, filtered, and evaporated in vacuo, affording 1.39 g (3.07 mmol) of the pure product **35** as a brown oil. TLC (cyclohexane/ethyl acetate 1:1). *R_f_* = 0.92. ^1^H NMR (CD_3_OD): δ 7.48–7.03 (m, 15H), 6.92–6.72 (m, 4H), 4.34 (dd, *J* = 11.3, 2.1 Hz, 1H), 4.08 (dd, *J* = 11.3, 6.6 Hz, 1H), 4.02–3.83 (m, 2H), 3.37–3.20 (m,2H), 2.20–2.03 (m, 1H), 1.76 (m, 1H).

#### 1-Azido-1-(1,4-benzodioxan)-3-trityloxypropane (**36**)

Nitrogen was bubbled in a solution of 1-(1,4-benzodioxan)-3-trityloxy-1-propanol **35** (1.23 g, 2.83 mmol) in 22 mL of dry toluene for 15 min. Then, triphenyl phosphine (816 mg, 3.11 mmol) and diethyl azodicarboxylate solution of 40 wt % in toluene (2.57 mL, 5.66 mmol) were added to the ice-cooled solution. After the reaction mixture was stirred at 0 °C for an hour, 1.22 mL of DPPA (5.66 mmol) was added dropwise. Then, the reaction mixture was warmed to room temperature and stirred until TLC indicated the disappearance of the starting material. Afterward, the solvent was evaporated in vacuo, affording an orangish oil. Flash chromatography (cyclohexane/ethyl acetate 9:1) was performed to obtain 430 mg (0.90 mmol) of the pure product **36** as a white oil. TLC (cyclohexane/ethyl acetate 9:1); *R_f_* = 0.67. ^1^H NMR (CD_3_OD): δ 7.56–7.09 (m, 15H), 6.94–6.72 (m, 4H), 4.30 (d, *J* = 9.1 Hz, 1H), 4.21–4.01 (m, 2 Hz), 3,88-3,72 (m, 1H), 2.14–1.94 (m, 1H), 1,94–1.75 (m, 1H).

#### 3-Azido-3-(1,4-benzodioxan)-1-propanol (**37**)

Amberlyst 15 (600 mg) was added to a solution of 1-azido-1-(1,4-benzodioxan)-3-trityloxypropane **36** (450 mg, 0.99 mmol) in 19 mL of methanol and 8 mL of dichloromethane. Then, the mixture was stirred at reflux until TLC indicated the disappearance of the starting material. Afterward, the solvent was evaporated in vacuo, affording a gray oil. Flash chromatography (cyclohexane/ethyl acetate 7:3) was performed to obtain 96 mg (0.41 mmol) of the pure product **37** as an orange oil. TLC (cyclohexane/ethyl acetate 9:1); *R_f_* = 0.17. ^1^H NMR (CD_3_OD): δ 6.88–6.69 (m, 4H), 4.27 (dd, *J* = 11.2, 2.2 Hz, 1H), 4.23–4.13 (m, 1H), 4.07 (dd, *J* = 11.2, 7.1 Hz, 1H), 3.81–3.58 (m, 3H), 1.98–1.85 (m, 2H).

#### 1-Azido-1-(1,4-benzodioxan)-propylmethanesulfonate (**38**)

3-Azido-3-(1,4-benzodioxan)-1-propanol **37** (300 mg, 1.43 mmol) was dissolved in dichloromethane (12 mL), and triethylamine (0.20 mL, 1.68 mmol) was added, and after the reaction mixture was cooled down to 0 °C, mesyl chloride (0.13 mL, 1.68 mmol) was added dropwise. The solution was stirred at room temperature until TLC indicated the disappearance of the starting material. The reaction mixture was washed with a 10% aqueous solution of HCl and then with brine. The organic phase was dried over anhydrous sodium sulfate and filtered, and the solvent was evaporated in vacuo, providing the pure product **38** as 413 mg of a yellow oil (1.32 mmol). TLC (dichloromethane/methanol 95:5); *R_f_* = 0.78. ^1^H NMR (CD_3_OD): 6.96–6.76 (m, 4H), 4.42 (dd, *J* = 7.1, 5.0 Hz, 2H), 4.37–4.18 (m, 2H), 4.13 (dd, *J* = 11.3, 6.8 Hz, 1H), 3.83–3.68 (m, 1H), 3.10 (s, 3H), 2.24–1.97 (m, 2H).

#### 4-Hydroxybenzenesulfonamide (**39**)

At 0 °C, 5 g of sulfanilamide (29 mmol) was dissolved in water (40 mL) and concentrated sulfuric acid (20 mL). Then, a solution of sodium nitrite (2 g, 29 mmol) in 20 mL of water was added dropwise, and the reaction was stirred at reflux until the evolution of nitrogen ceased. The mixture was cooled down to room temperature and kept overnight at 4 °C. Afterward, crystals of **39** (3.78 g, 21.83 mmol) were collected by filtration. Mp 175 °C. ^1^H NMR (CDCl_3_): δ 7.73 (d, *J* = 8.8 Hz, 2H), 6.87 (d, *J* = 8.8 Hz, 2H).

#### *N*,*N*-Dimethylaminomethylene-4-hydroxybenzenesulfonamide (**40**)

*N*,*N*-Dimethylformamide dimethyl acetal (3.87 mL, 29.12 mmol) was added to a solution of 4-hydroxybenzenesulfonamide **39** (4.20 g, 24.27 mmol) in dimethylformamide (4 mL) at 0 °C, and the mixture was stirred for 2 h at room temperature. The reaction mixture was treated with ethyl acetate, and the obtained white solid was filtered to afford the title compound **40** (5.21 g, 22.82 mmol). Mp 178 °C. ^1^H NMR (CD_3_OD): δ 8.12 (s).

#### 3-Bromo-4-hydroxybenzenesulfonamide (**41**)

To a stirred solution of 2 g of 4-hydroxybenzenesulfonamide **39** (11.55 mmol) in tetrahydrofuran (10 mL) was added NBS (2.06 g, 11.55 mmol) at −20 °C. The reaction mixture was stirred at the same temperature until TLC indicated the disappearance of the starting material. After that, the reaction mixture was evaporated under reduced pressure and the crude product was diluted with ethyl acetate. The organic phase was washed with a 10% aqueous solution of NaHCO_3_ and brine, dried over anhydrous sodium sulfate, and filtered. The solvent was then evaporated in vacuo, providing a green oil. Flash chromatography (cyclohexane/ethyl acetate 1:1) was performed to obtain 972 mg (3.85 mmol) of the pure product **41** as a white oil. TLC (cyclohexane/ethyl acetate 3:7); *R_f_* = 0.70. Mp 161 °C. ^1^H NMR (CDCl_3_): δ 7.99 (d, *J* = 2.3 Hz, 1H), 7.69 (dd, *J* = 8.6, 2.3 Hz, 1H), 6.97 (d, 8.6 Hz, 1H).

#### 3-Methoxy-4-hydroxybenzenesulfonamide (**42**)

Under a nitrogen atmosphere, 3-bromo-4-hydroxybenzenesulfonamide **41** (270 mg, 1.07 mmol) and CuI (815 g, 4.28 mmol) were dissolved in 10 mL of a solution of sodium methoxide. The mixture was stirred at reflux for 18 h, checking the progress of the reaction via TLC. Afterward, the mixture was cooled down to room temperature and it was acidified with dilute hydrochloric acid and extracted with ethyl acetate. The organic layers were washed with brine and dried over anhydrous sodium sulfate. After evaporating to dryness under reduced pressure, 215 mg of crude was obtained. Flash chromatography (cyclohexane/ethyl acetate 6:4) was performed to obtain 160 mg (0.78 mmol) of the pure product **42** as a white solid. TLC (cyclohexane/ethyl acetate 7:3); *R_f_* = 0.52. Mp 103 °C. ^1^H NMR (CDCl_3_): δ 7.42 (d, *J* = 2.2, 1H), 7.37 (dd, *J* = 8.3, 2.2 Hz, 1H), 6.89 (d, *J* = 8.3 Hz, 1H), 3.78 (s, 3H).

#### *N*,*N*-Dimethylaminomethylene-3-methoxy-4-hydroxybenzenesulfonamide (**43**)

*N*,*N*-Dimethylformamide diethyl acetal (0.12 mL, 0.94 mmol) was added to a solution of 3-methoxy-4-hydroxybenzenesulfonamide **42** (160 mg, 0.78 mmol) in dimethylformamide (1 mL) at 0 °C and the mixture was stirred for 2 h at room temperature; the starting material was consumed (TLC monitoring). The reaction mixture was treated with ethyl acetate, and the obtained solid was filtered to afford the title compound **43** as a white solid (223 mg, 0.86 mmol). TLC (dichloromethane/methanol 95:5); *R_f_* = 0.59. Mp 108 °C. ^1^H NMR (CDCl_3_): δ 8.12 (s, 1H), 7.47 (dd, *J* = 7.5, 1.4 Hz, 1H), 7.41 (d, *J* = 1.3 Hz, 1H), 7.04 (d, *J* = 7.5 Hz, 1H), 3.78 (s, 3H), 3.10 (s, 3H), 2.88 (s, 3H).

#### 3,5-Dibromo-4-hydroxybenzenesulfonamide (**44**)

A solution of bromine (1.47 mL, 28.87 mmol) in 12 mL of dichloromethane and methanol in a mixing ratio of 1:1 was added dropwise to an ice-cooled solution of 2 g of 4-hydroxybenzenesulfonamide **39** (11.55 mmol) dissolved in the same solvent mixture. Then, the reaction mixture was stirred at room temperature until TLC indicated the disappearance of the starting material. The solid was filtered and washed with dichloromethane to obtain the desired product **44** as a white solid (3.28 g, 9.93 mmol). TLC (cyclohexane/ethyl acetate 7:3); *R_f_* = 0.86. Mp 228 °C. ^1^H NMR (CDCl_3_): δ 7.97 (s, 2H).

#### 3,5-Dimethoxy-4-hydroxybenzenesulfonamide (**45**)

Under a nitrogen atmosphere, 3,5-dibromo-4-hydroxybenzenesulfonamide **44** (2.04 g, 6.16 mmol) and CuI (4.70 g, 2.64 mmol) were dissolved in 20 mL of a solution of sodium methoxide. The mixture was stirred at reflux for 18 h, checking the progress of the reaction via TLC. Afterward, the mixture was cooled down to room temperature, acidified with dilute hydrochloric acid, and extracted with ethyl acetate. The organic layers were washed with brine and dried over anhydrous sodium sulfate. After evaporating to dryness under reduced pressure, 934 mg of crude was obtained. Flash chromatography (cyclohexane/ethyl acetate 6:4) was performed to obtain 160 mg (0.68 mmol) of the pure product **44** as a white solid. TLC (cyclohexane/ethyl acetate 7:3); *R_f_* = 0.26. Mp 165 °C. ^1^H NMR (CDCl_3_): δ 7.18 (s, 2H), 3.90 (s, 6H).

#### *N*,*N*-Dimethylaminomethylene-3,5-dimethoxy-4-hydroxybenzenesulfonamide (**46**)

*N*,*N*-Dimethylformamide diethyl acetal (0.93 mL, 6.96 mmol) was added to a solution of 3,5-dimethoxy-4-hydroxybenzenesulfonamide **45** (1 g, 4.28 mmol) in dimethylformamide (2 mL) at 0 °C, and the mixture was stirred for 2 h at room temperature, checking the progress of the reaction via TLC. The reaction mixture was treated with ethyl acetate, and the obtained solid was filtered to afford the title compound **46** as a white solid (1.13 g, 3.94 mmol). TLC (dichloromethane/methanol 95:5); *R_f_* = 0.59. Mp 171 °C. ^1^H NMR (CDCl_3_): δ 8.12 (s, 1H), 6.96 (s, 2H), 3.78 (s, 6H), 3.10 (s, 3H), 2.88 (s, 3H).

### In Vitro DPP IV Activity Assay

Four independent in vitro experiments were carried out, and each of those was performed in triplicate in a white half-volume 96-well solid plate. Each reaction (50 μL) was prepared by adding the reagents in a microcentrifuge tube in the following order: 1× assay buffer [20 mM tris–HCl, pH 8.0, containing 100 mM NaCl and 1 mM EDTA] (30 μL), final compounds (**1**–**14**) (from 10^–10^ to 10^–3^ M) or vehicle (10 μL), and finally the DPP IV enzyme (10 μL). Afterward, the samples were mixed and 50 μL was transferred to each plate well. Each reaction was started by adding 50 μL of the substrate solution (200 μM H-Gly-Pro-7-amido-4-methylcoumarin (AMC)) to each well and incubated at 37 °C for 30 min. Fluorescence signals were measured using a Synergy H1 fluorescent plate reader from Biotek (excitation and emission wavelengths 360 and 465 nm, respectively). The DPP IV enzyme and the substrate solution were provided by Cayman Chemicals (Michigan).

### Evaluation of the Inhibitory Effect of Compound **12** on Cellular DPP IV Activity

A total of 3 × 10^4^ Caco-2 cells/well were seeded in black 96-well plates with clear bottoms. On the second day after seeding, the spent media was discarded and the cells were treated with 100.0 nM and 5.0 μM of compound **12** and with the reference compound sitagliptin at final concentrations of 20.0 nM and 1.0 μM, or vehicle, in growth medium for 60 min at 37 °C. Subsequently, treatments were removed, and Caco-2 cells were washed once with 100 μL of PBS, before the addition to each well of 50 μL of Gly-Pro-AMC substrate at a concentration of 25 μM in PBS. The fluorescence signal (excitation and emission wavelengths 350 and 450 nm, respectively) was recorded using a Synergy H1 microplate reader.

### Cell Culture

HepG2 cells were bought from ATCC (HB-8065, ATCC from LGC Standards, Milan, Italy), and Caco-2 cells were obtained from INSERM (Paris, France). HepG2 was cultured in DMEM high glucose with stable l-glutamine, supplemented with 10% fetal bovine serum (FBS), 100 U/mL penicillin, and 100 μg/mL streptomycin (complete growth medium) with incubation at 37 °C under a 5% CO_2_ atmosphere. Caco-2 cells were routinely subcultured at a 50% density and maintained under the same HepG2 cultured conditions.

### 3-(4,5-Dimethylthiazol-2-yl)-2,5-diphenyltetrazolium Bromide (MTT) Assay

A total of 3 × 10^4^ Caco-2 and HepG2 cells/well were seeded in 96-well plates and treated with **11** and **12** from 10^–8^ to 10^–5^ M or vehicle (H_2_O) in complete growth media for 48 h at 37 °C under a 5% CO_2_ atmosphere. Subsequently, the treatment solvent was aspirated and 100 μL/well of 3-(4,5-dimethylthiazol-2-yl)-2,5-diphenyltetrazolium bromide (MTT) filtered solution was added. After 2 h of incubation at 37 °C under a 5% CO_2_ atmosphere, 0.5 mg/mL solution was aspirated and 100 μL/well of the lysis buffer (8 mM HCl + 0.5% NP-40 in DMSO) was added. After 10 min of slow shaking, the absorbance at 575 nm was read on a Synergy H1 fluorescence plate reader (Biotek, Bad Friedrichshall, Germany).

### CA Inhibition

An Applied Photophysics stopped-flow instrument was used to assay the CA-catalyzed CO_2_ hydration activity.^37^ Phenol red (at a concentration of 0.2 mM) was used as an indicator, working at an absorbance maximum of 557 nm, with 20 mM Hepes (pH 7.4) as a buffer, and 20 mM Na_2_SO_4_ (to maintain constant ionic strength), following the initial rates of the CA-catalyzed CO_2_ hydration reaction for a period of 10–100 s. The CO_2_ concentrations ranged from 1.7 to 17 mM for the determination of the kinetic parameters and inhibition constants.^43^ Enzyme concentrations ranged between 5 and 12 nM. For each inhibitor, at least six traces of the initial 5–10% of the reaction were used to determine the initial velocity. The uncatalyzed rates were determined in the same manner and subtracted from the total observed rates. Stock solutions of the inhibitor (0.1 mM) were prepared in distilled–deionized water, and dilutions up to 0.01 nM were done thereafter with the assay buffer. Inhibitor and enzyme solutions were preincubated together for 15 min at room temperature prior to the assay to allow for the formation of the E–I complex. The inhibition constants were obtained by nonlinear least-squares methods using PRISM 3 and the Cheng–Prusoff equation, as reported earlier, and represent the mean from at least three different determinations. All CA isoforms were recombinant proteins obtained in-house, as reported earlier.^44−46^

### Computational Methods

The crystal of human DPP IV was retrieved from RCSB PDB (entry code: 1X70)^47^ in complex with ligand 715 (sitagliptin), and the crystal of human CA II was likewise retrieved from RCSB PDB (entry code: 3k34)^48^ in complex with ligand SUA (a benzenesulfonamide inhibitor). Since DPP IV is a homodimer, the best chain has been evaluated, choosing the one with the lower number of outliers and the best fitting according to section 6 “Fit of the model and data” of the PDB report.

The obtained structures were then minimized using AMMP, as implemented in the VEGA environment.^49^ Both the crystals were then prepared, adding eventual missing residues and checking for alternative conformations of the side chains, chirality, trans-peptide bond, and ring interaction. Hydrogen atoms were added to all crystal structures according to the physiological pH, and they underwent an energy minimization using NAMD2,^50^ CHARMM22 as a force field, and constraints on the backbone atoms. To prevent the binding site collapse, the respective ligand was inserted into their crystal structure. Moreover, for CA II, the Zn^2+^ ion was reintroduced into the structure by overlapping it with the PDB downloaded structure.

Both binding sites were calculated by redocking, and the bound ligands were removed from the structure for docking experiments. The docking simulations were performed by PLANTS, focusing the search on a 10.0 Å radius sphere around the bound ligands. The conformational profile of the considered ligands was explored by Monte Carlo procedures, as described elsewhere.^51^ For each ligand, 10 poses were generated and scored by the ChemPLP score with a speed equal to 1. The complexes were finally minimized by keeping fixed all atoms outside a 10 Å radius sphere around the bound ligand. Interactions between the most potent DDP IV inhibitor compounds and the active site of DPP IV were further investigated. Since compounds of the second and third generations are characterized by two chiral centers, molecular docking investigations were performed on all four diastereomers. For the sake of simplicity, we report only the best results.

## Supporting Information Available

Supplementary Tables and Figures; ^1^H NMR and ^13^C NMR spectra; and HPLC purity analyses and HRMS of the final compounds (PDF)
SMILES chemical notation for the final compounds (CSV)

## Abbreviations Used

AR: adrenergic receptor
Boc_2_O: di-*tert*-butyl dicarbonate
DCM: dichloromethane
DEAD: diethyl azodicarboxylate
DMF: *N*,*N*-dimethylformamide
DMF-DMA: *N*,*N*-dimethylformamide dimethyl acetal
DPPA: diphenylphosphorylazide
IPA: isopropanol
MeOH: methanol
MsCl: mesyl chloride
NBS: *N*-bromosuccinimide
SAR: structure–activity relationship
TBAB: tetrabutylammonium bromide
TBDMSiCl: tert-butyldimethyl silylchloride
TEA: triethylamine
THF: tetrahydrofuran
TrtCl: trityl chloride
