# Supplementary material for: Discovery of a Potent and Highly Selective Dipeptidyl Peptidase IV and Carbonic Anhydrase Inhibitor as “Antidiabesity” Agents Based on Repurposing and Morphing of WB-4101
Source: J Med Chem. 2022 Oct 6;65(20):13946–66. doi: 10.1021/acs.jmedchem.2c01192 (PMC9937538; doi:10.1021/acs.jmedchem.2c01192)
Supplement: Supplementary file 1 — jm2c01192_si_001.pdf [file jm2c01192_si_001.pdf]

Supporting Information

Discovery of a Potent and Highly Selective Dipeptidyl Peptidase IV and Carbonic Anhydrase Inhibitor as "Antidiabesity" Agent Based on Repurposing and Morphing of WB-4101

Angelica Artasensi†, Andrea Angeli‡, Carmen Lammi†, Carlotta Bollati†, Silvia Gervasoni†⊥, Giovanna Baron†, Rosanna Matucci§, Claudiu T. Supuran‡, Giulio Vistoli† , Laura Fumagalli*†.

†Università degli Studi di Milano, Department of Pharmaceutical Sciences "DISFARM", Via Mangiagalli 25, I-20133 Milan, Italy;
‡ University of Florence, Department of Pharmaceutical Sciences "NEUROFARBA", Via Ugo Schiff 6, 50019 Sesto Fiorentino, Florence, Italy;
⊥ University of Cagliari, Department of Physics, Citt. Universitaria, I-09042 Monserrato, Cagliari, Italy;
§University of Florence, Department of Pharmacology and Toxicology "NEUROFARBA", Viale Pieraccini 6, 50134 Florence, Italy.

Corresponding Author:
*Laura Fumagalli - Università degli Studi di Milano, Department of Pharmaceutical Sciences "DISFARM", Via Mangiagalli 25, I-20133 Milan, Italy;
Email: laura.fumagalli@unimi.it

TABLE OF CONTENTS

Supplementary tables and figures 2
^{1}H and ^{13}C NMR spectra of final compounds **1-14** 6
Mass and HPLC spectra of final compounds **1-14** 20

SUPPLEMENTARY TABLES AND FIGURES

Figure S1. Preliminary docking investigations on WB-4101 para substituted with a sulfonamide moiety in the binding site of CA-II. (PDB ID: 3k34)

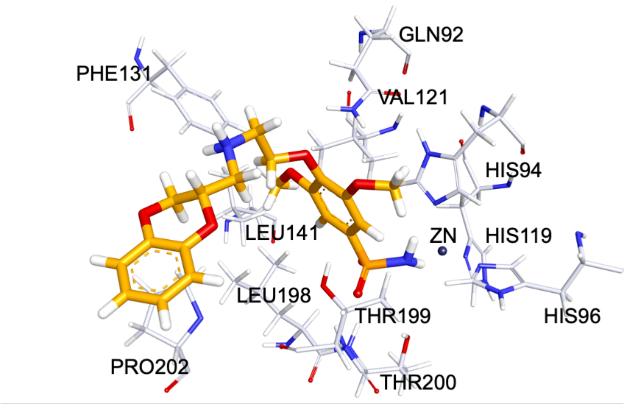

Figure S2. Preliminary docking investigations on WB-4101 para substituted with a sulfonamide group in the binding site of DPP-IV (PDB ID: 1x70).

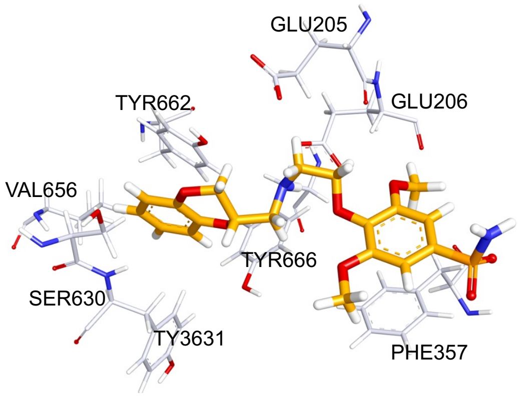

Figure S3. Inhibition of $[^3H]$prazosin specific binding by **11** and **12** in membrane homogenates of CHO cells stably expressing the α_{1A}-AR. Curves from single experiments were fitted using the standard four parameter logistic equation and represent the mean ± S.E.M. of 4 observations, 2 experiments. X-axis: Log concentrations of competitive test compounds. Y-axis: normalized $[^3H]$prazosin Bound/Total bound.
In the table are reported the affinity constants, expressed as pKi (-log Ki), of compounds **11**, **12** and WB-4101 for human cloned α_{1a}-AR expressed in HEK293 cells. The values represent the arithmetic mean ± S.E.M. of 4 observations, 2 experiments.

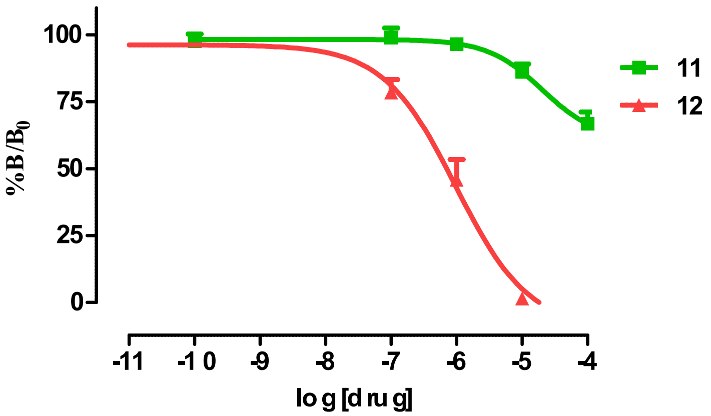

Compound	pK_i	*nH*
**11**	<4	ND
**12**	6.61±0.11	0.97±0.27
WB-4101[1]	9.36 ± 0.04	NA

ND, not determinable; NA, not available.

Note: data generated from binding assays were analyzed using Prism 5.02 (GraphPad Software Inc., San Diego, CA) and were fitted to a parametric function to derive best estimates of the IC_{50} and slope factor (nH); IC_{50} values were then converted to binding constant Ki according to Cheng-Prusoff equation.
The details on the α1a-AR binding assay have been described before[2].

Figure S4. MTT assay to quantify primary hepatocyte and Caco-2 cells viability after treatment with compounds 11 in purple (**A**) and 12 in blue (**B**).

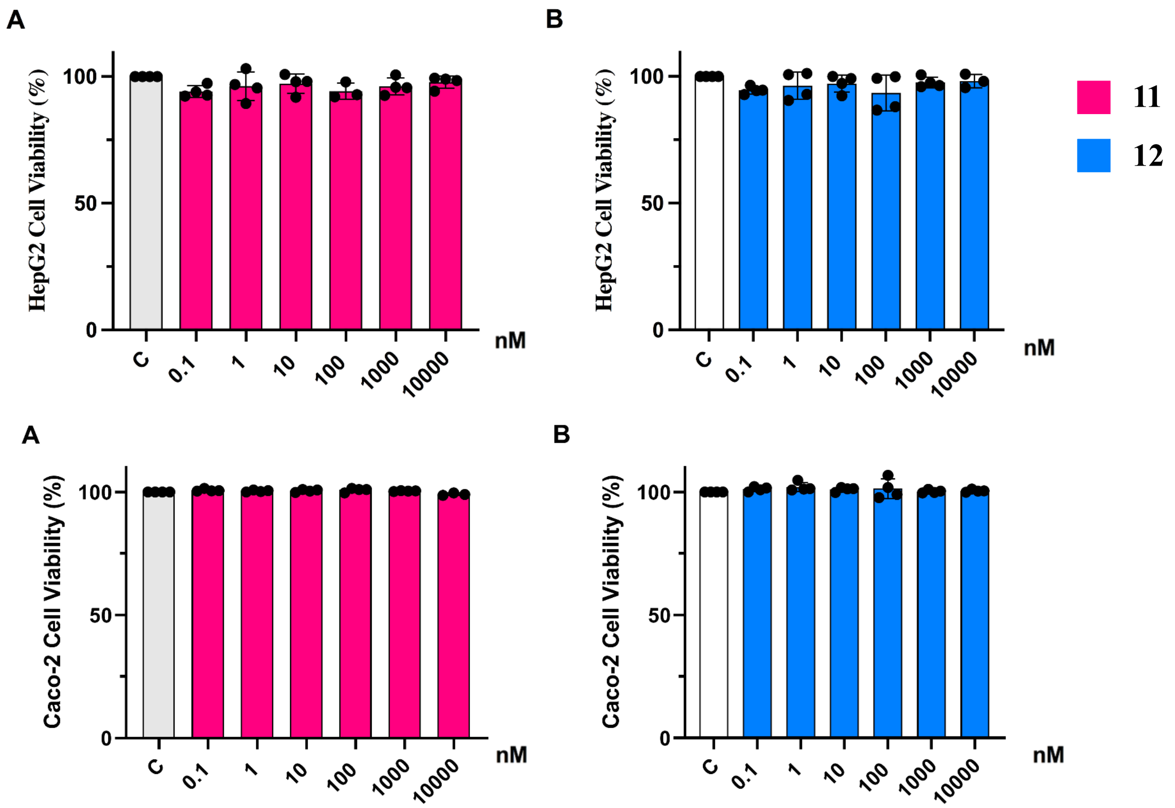

Table S1. *In silico* pharmacokinetic and pharmacodynamics properties of compounds **11** and **12**.

	**WB-4101 HCl**	**11**	**12**
Lipophilicity			
iLOGP	0.00	2.82	0.00
XLOGP3	3.69	3.18	2.22
WLOGP	3.31	3.72	-1.36
MLOGP	1.43	-0.29	-3.12
Silicos-IT Log P	3.15	0.57	0.93
Consensus Log P	2.32	2.00	-0.27
Water Solubility			
ESOL Log S	-4.35	-4.33	-3.67
ESOL Solubility (mg/ml)	1.72e-02	2.11e-02	8.58e-02
ESOL Solubility (mol/l)	4.51e-05	4.69e-05	2.14e-04
ESOL Class	Moderately soluble	Moderately soluble	Soluble
Pharmacokinetics			
Ali Log S	-4.60	-6.30	-4.46
Ali Solubility (mg/ml)	9.55e-03	2.24e-04	1.40e-02
Ali Solubility (mol/l)	2.50e-05	4.96e-07	3.50e-05
Ali Class	Moderately soluble	Poorly soluble	Moderately soluble
Silicos-IT LogSw	-5.94	-5.16	-4.47
Silicos-IT Solubility (mg/ml)	4.37e-04	3.15e-03	1.35e-02
Silicos-IT Solubility (mol/l)	1.15e-06	7.00e-06	3.37e-05
Silicos-IT class	Moderately soluble	Moderately soluble	Moderately soluble
GI absorption	High	Low	High
BBB permeant	Yes	No	No
Pgp substrate	Yes	No	No
CYP1A2 inhibitor	No	No	No
CYP2C19 inhibitor	Yes	Yes	No
CYP2C9 inhibitor	No	No	No
CYP2D6 inhibitor	Yes	Yes	No
CYP3A4 inhibitor	Yes	No	No
log Kp (cm/s)	-6.01	-6.79	-7.17
Druglikeness			
Lipinski #violations	0	1	0
Ghose #violations	0	0	1
Veber #violations	0	1	0
Egan #violations	0	1	0
Muegge #violations	0	2	0
Bioavailability Score	0.55	0.11	0.55
Medicinal Chemistry			
PAINS #alerts	0	1	0
Brenk #alerts	0	3	0
Leadlikeness #violations	3	2	1
Synthetic Accessibility	3.53	4.54	3.88

^{1}H AND ^{13}C NMR SPECTRA OF FINAL COMPOUNDS 1-14

Figure S5. ^{1}H NMR spectrum of compound **1**

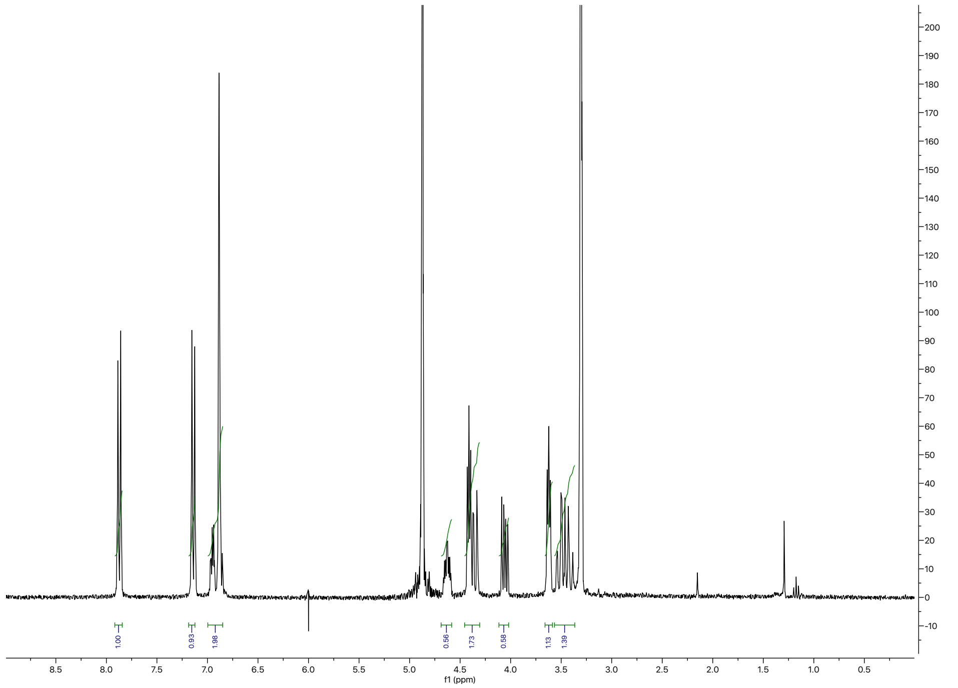

Figure S6. ^{13}C NMR spectrum of compound **1**

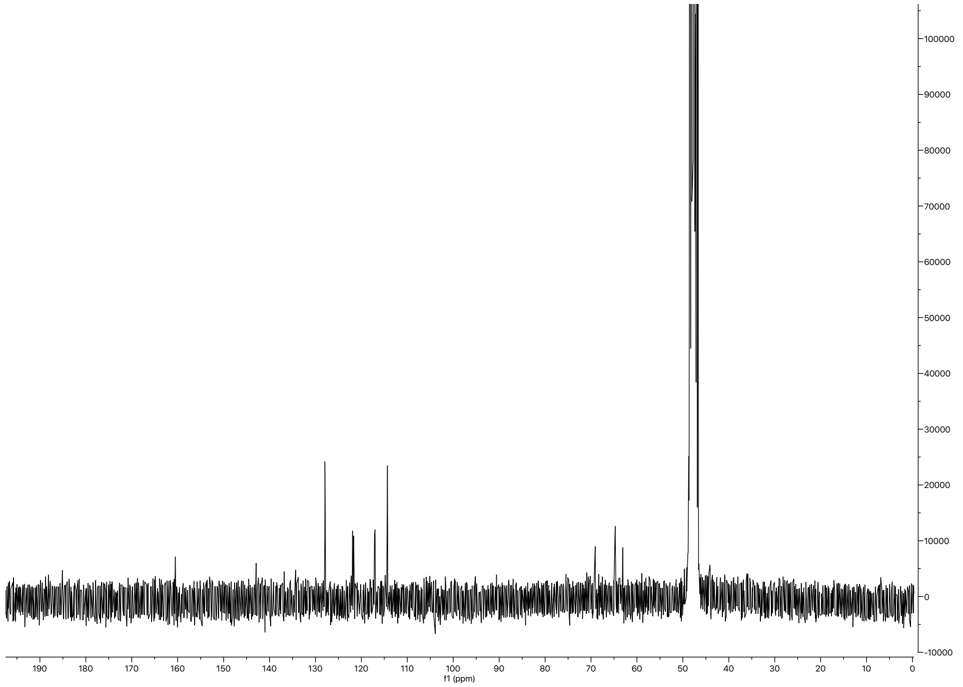

Figure S7. ^{1}H NMR spectrum of compound **2**

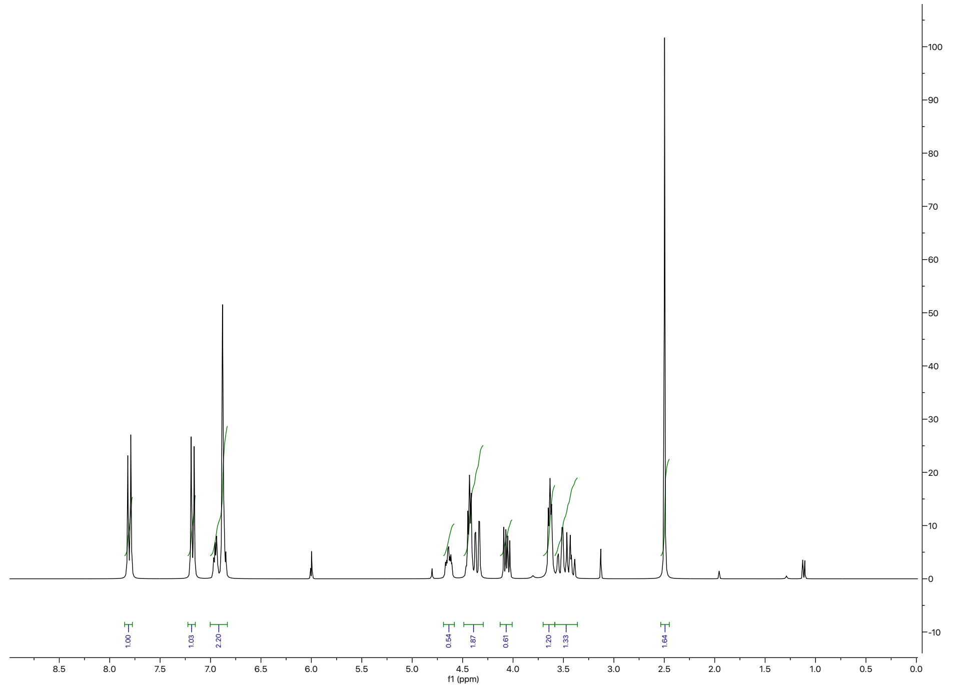

Figure S8. ^{13}C NMR spectrum of compound **2**

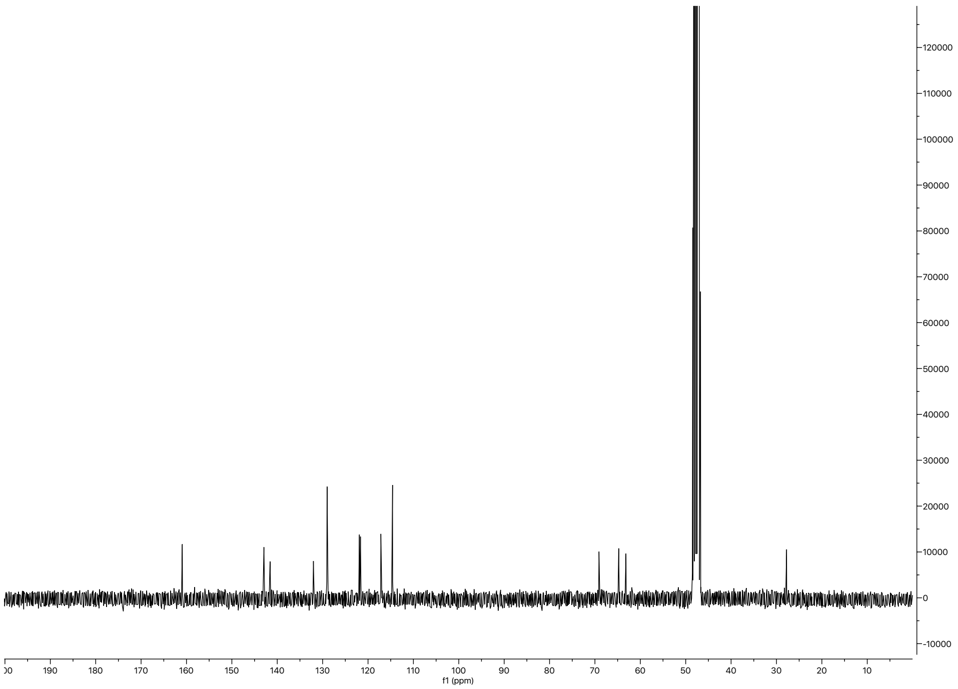

Figure S9. ^{1}H NMR spectrum of compound **3**

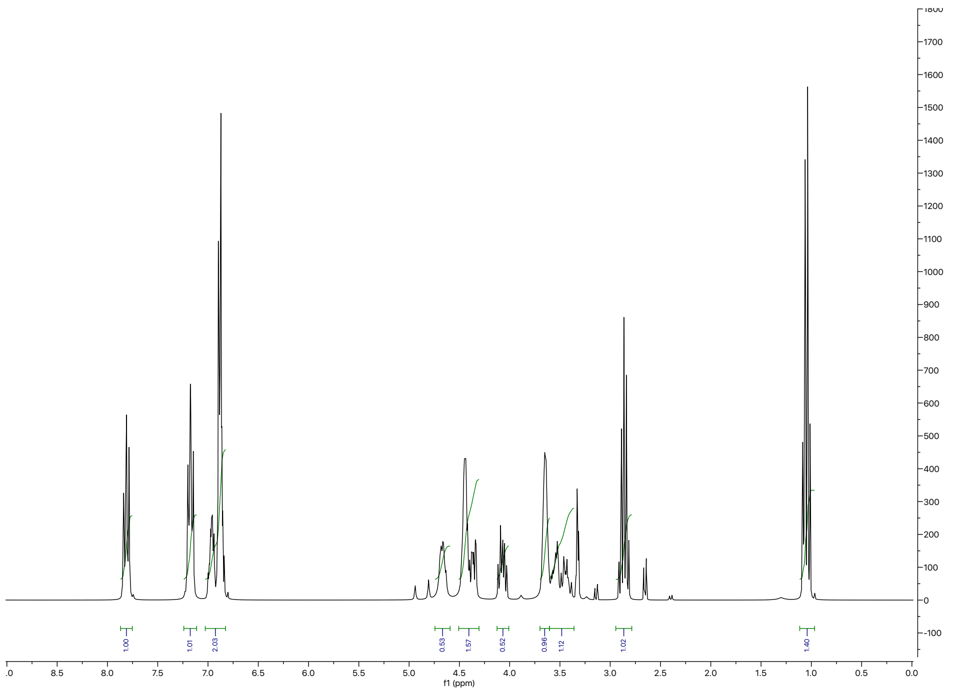

Figure S10. ^{13}C NMR spectrum of compound **3**

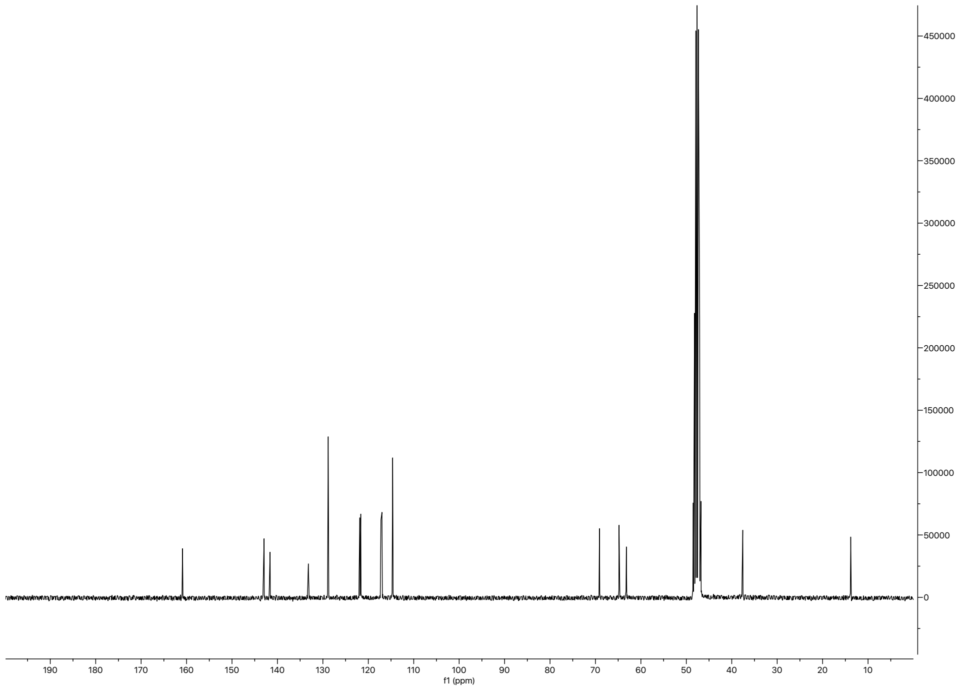

Figure S11. ^{1}H NMR spectrum of compound **4**

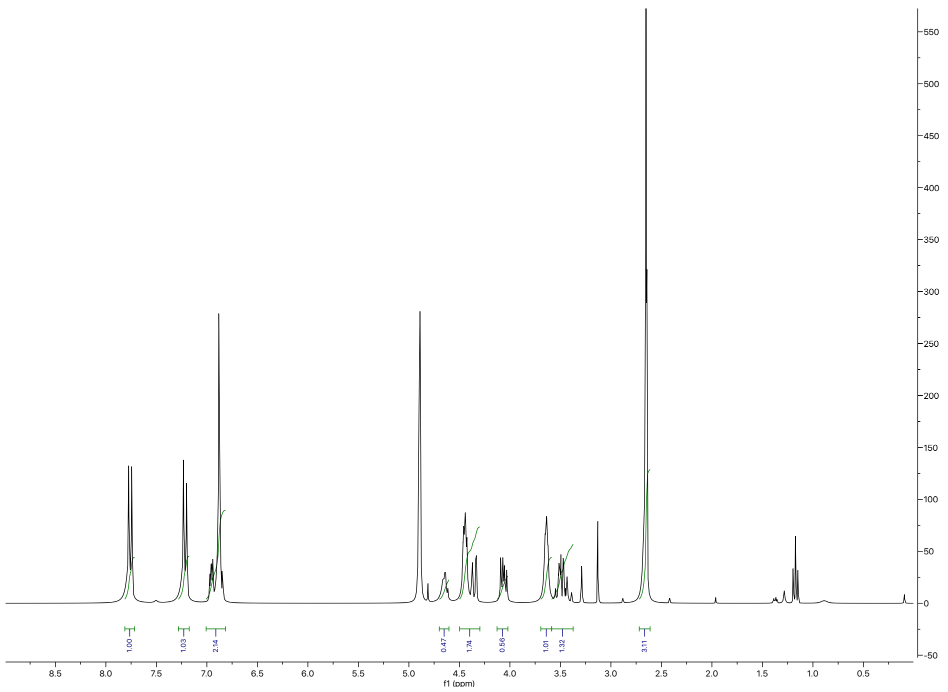

Figure S12. ^{13}C NMR spectrum of compound **4**

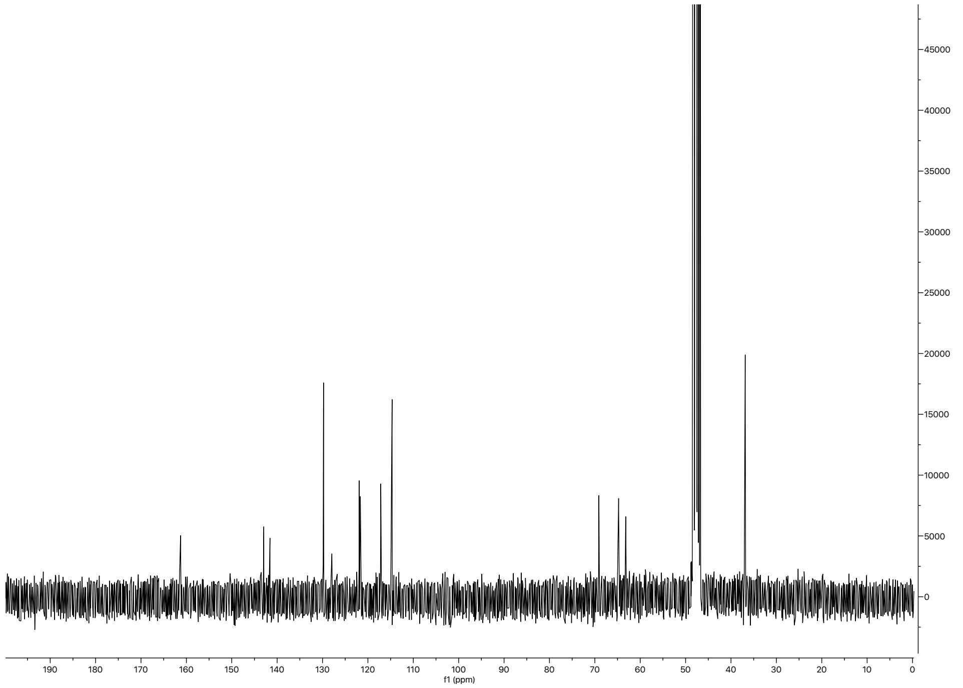

Figure S13. ^{1}H NMR spectrum of compound **5**

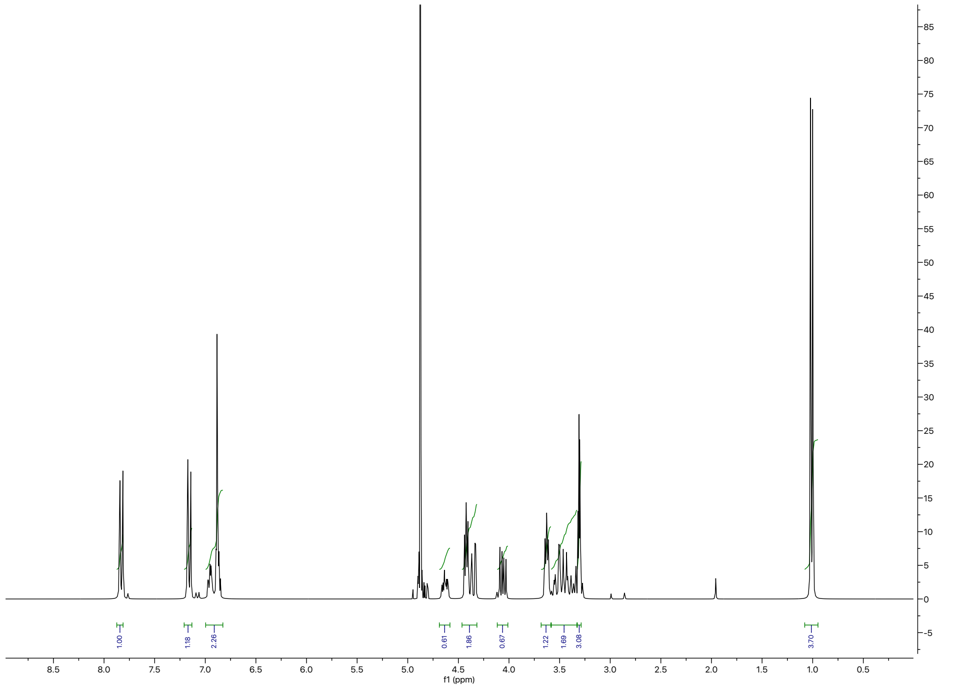

Figure S14. ^{13}C NMR spectrum of compound **5**

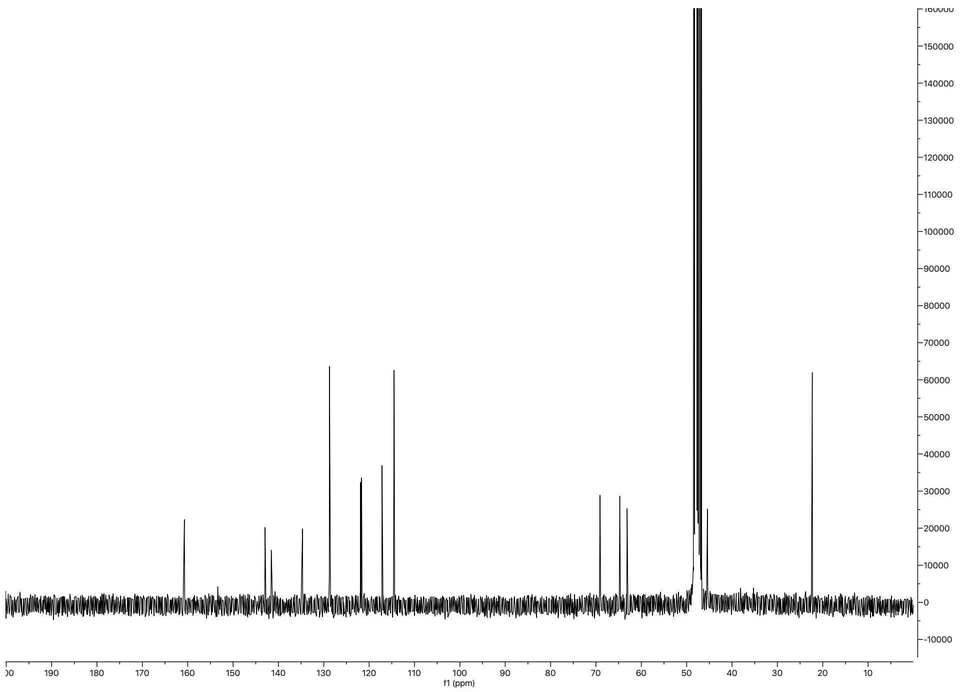

Figure S15. ^{1}H NMR spectrum of compound **6**

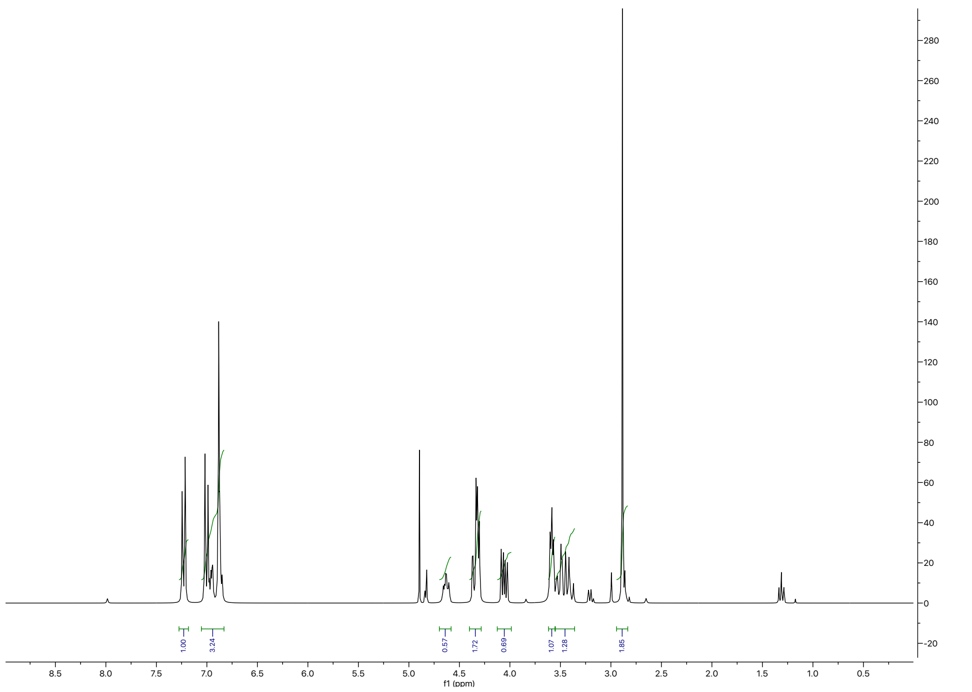

Figure S16. ^{13}C NMR spectrum of compound **6**

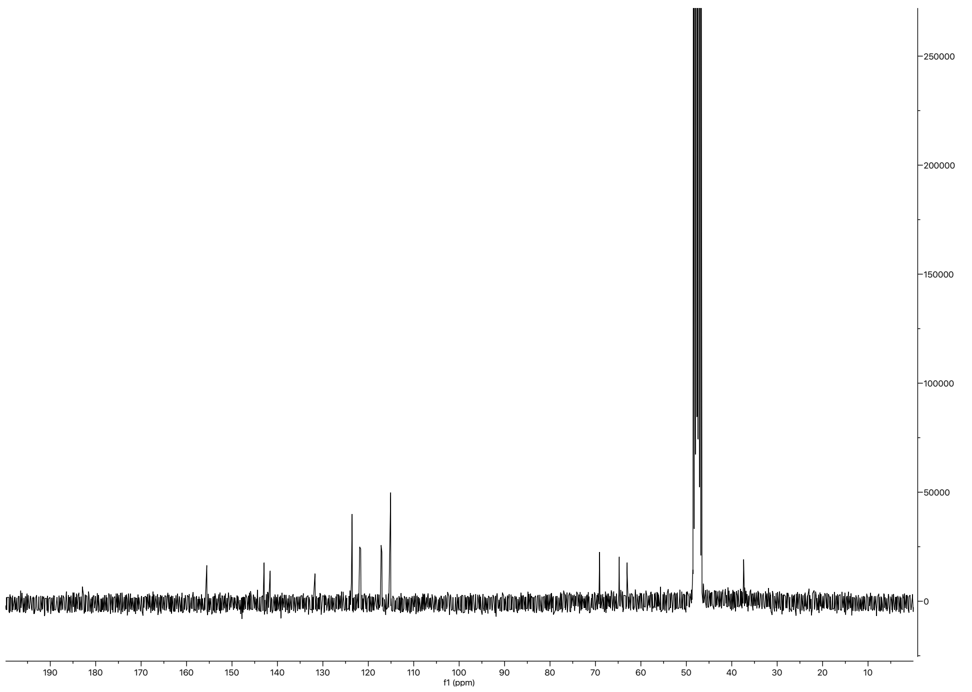

Figure S17. ^{1}H NMR spectrum of compound **7**

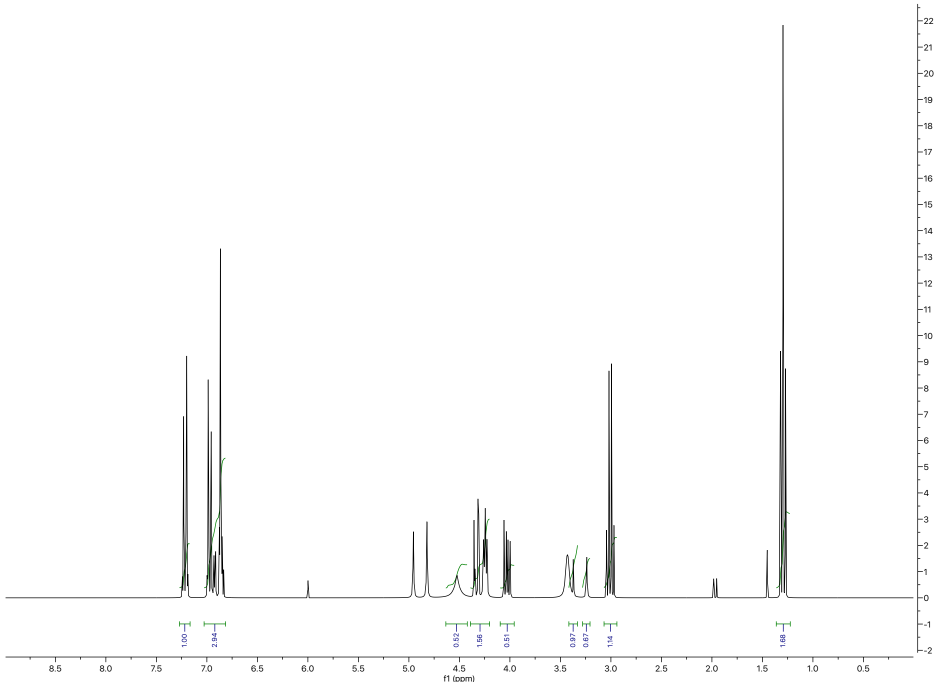

Figure S18. ^{13}C NMR spectrum of compound **7**

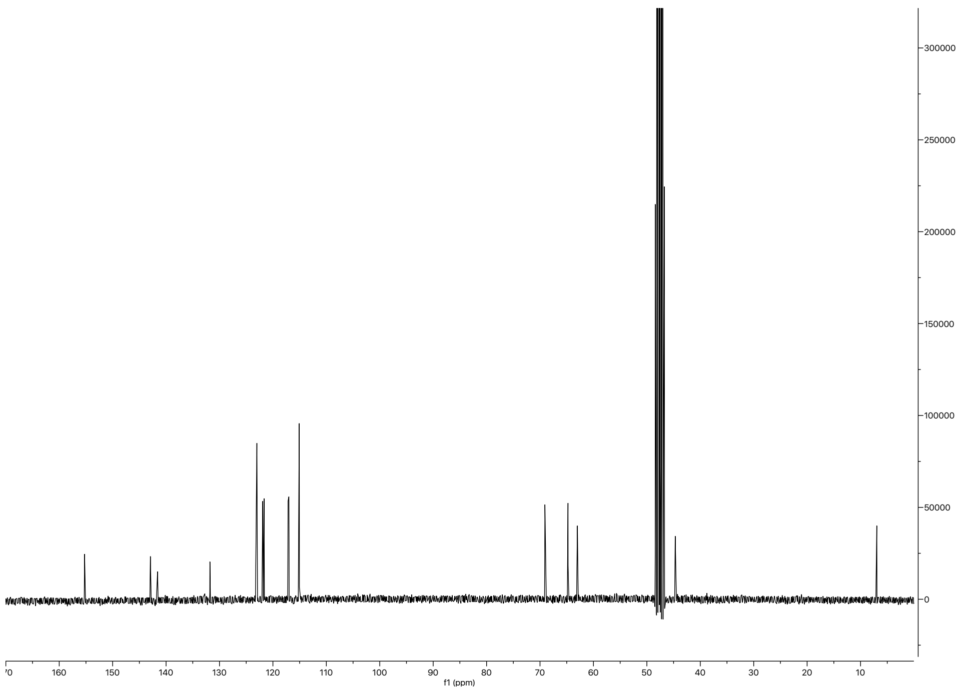

Figure S19. ^{1}H NMR spectrum of compound **8**

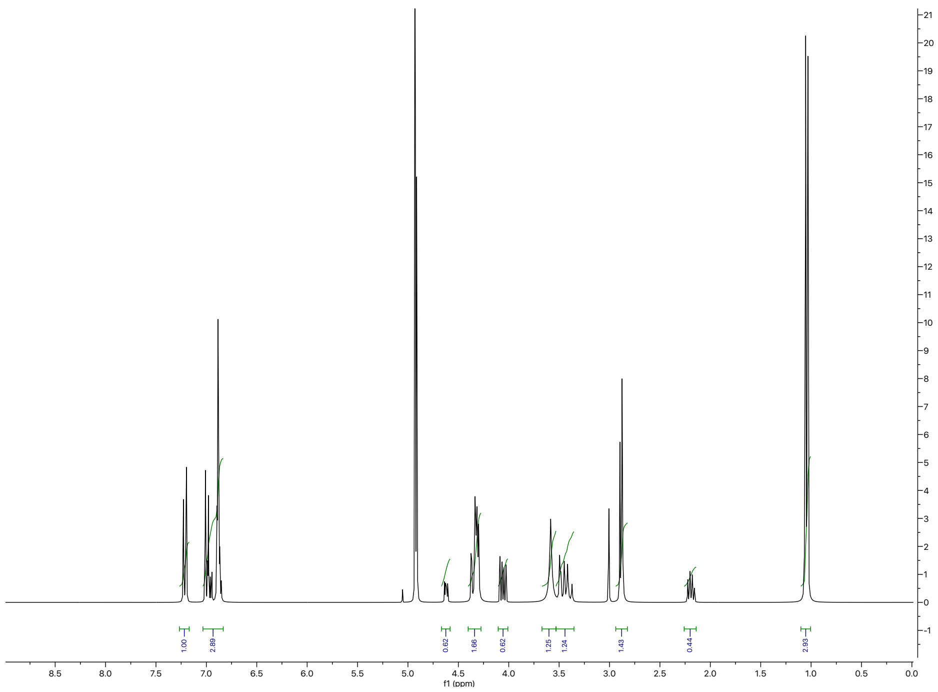

Figure S20. ^{13}C NMR spectrum of compound **8**

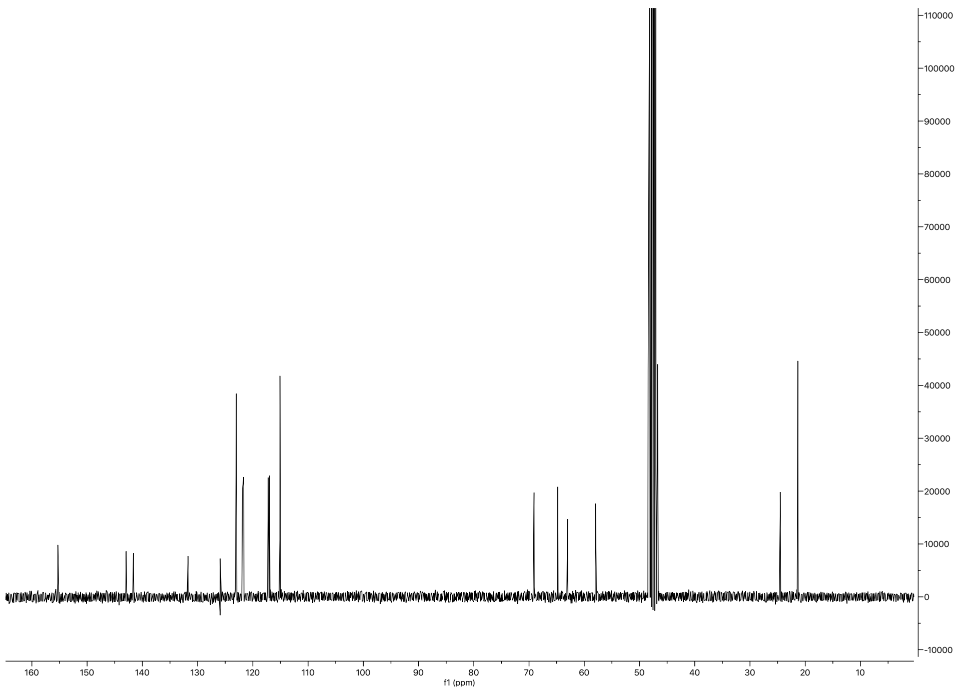

Figure S21. ^{1}H NMR spectrum of compound **9**

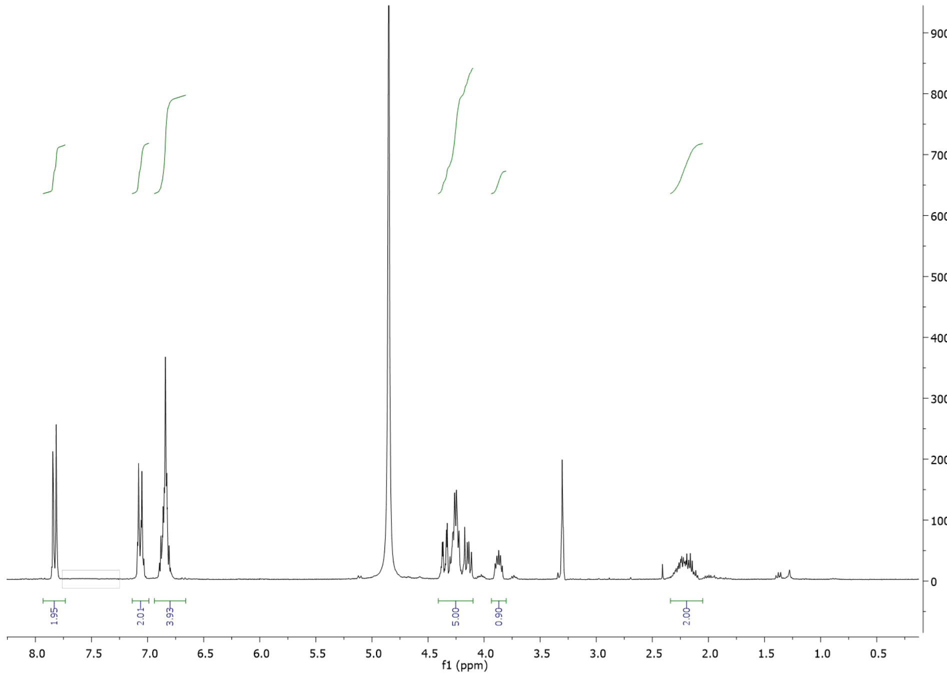

Figure S22. ^{13}C NMR spectrum of compound **9**

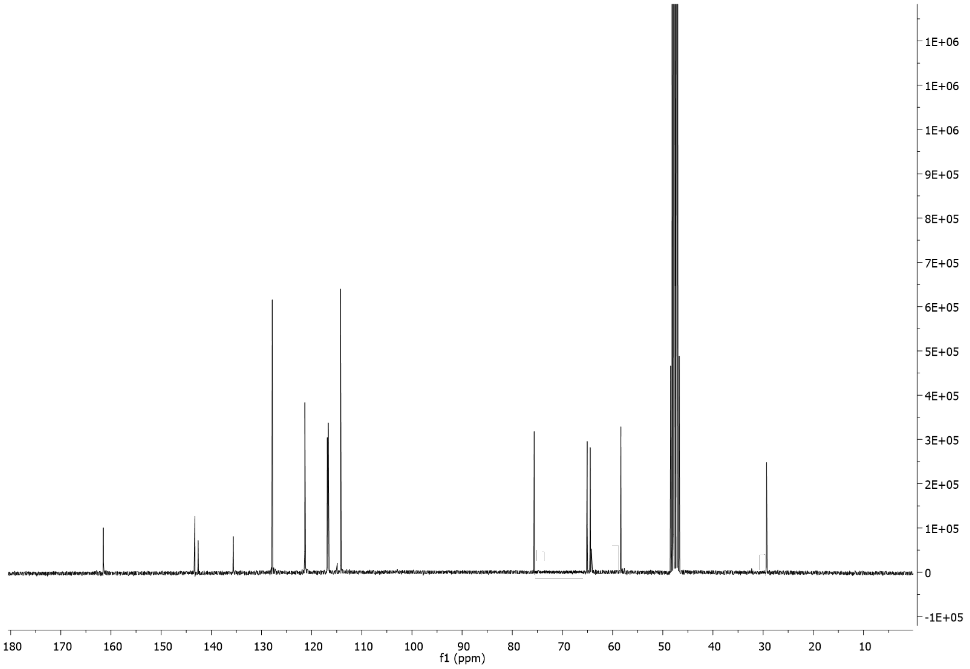

Figure S23. ^{1}H NMR spectrum of compound **10**

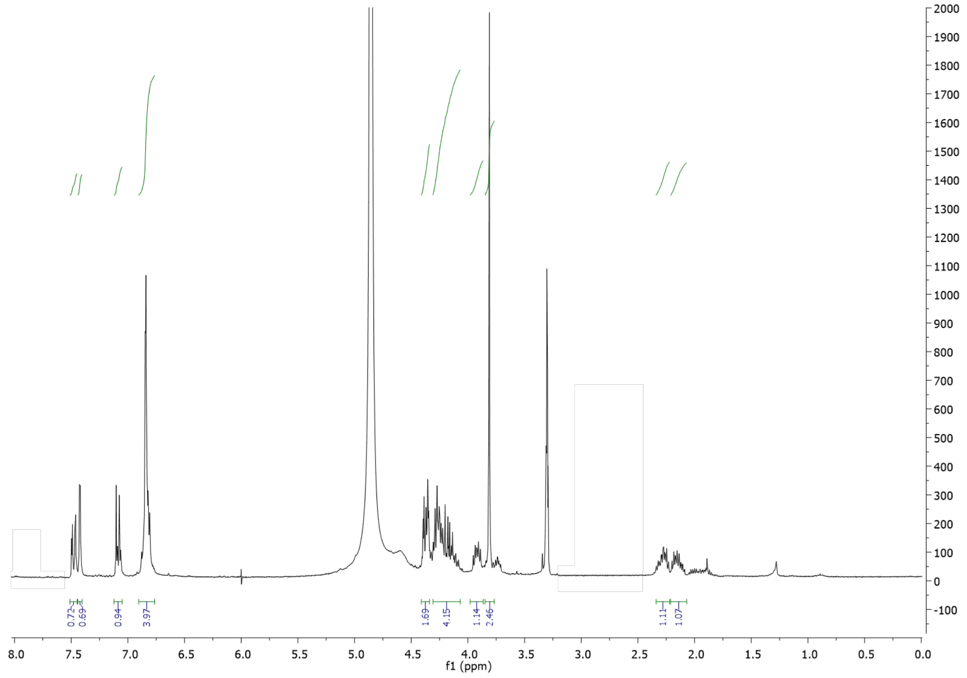

Figure S24. ^{13}C NMR spectrum of compound **10**

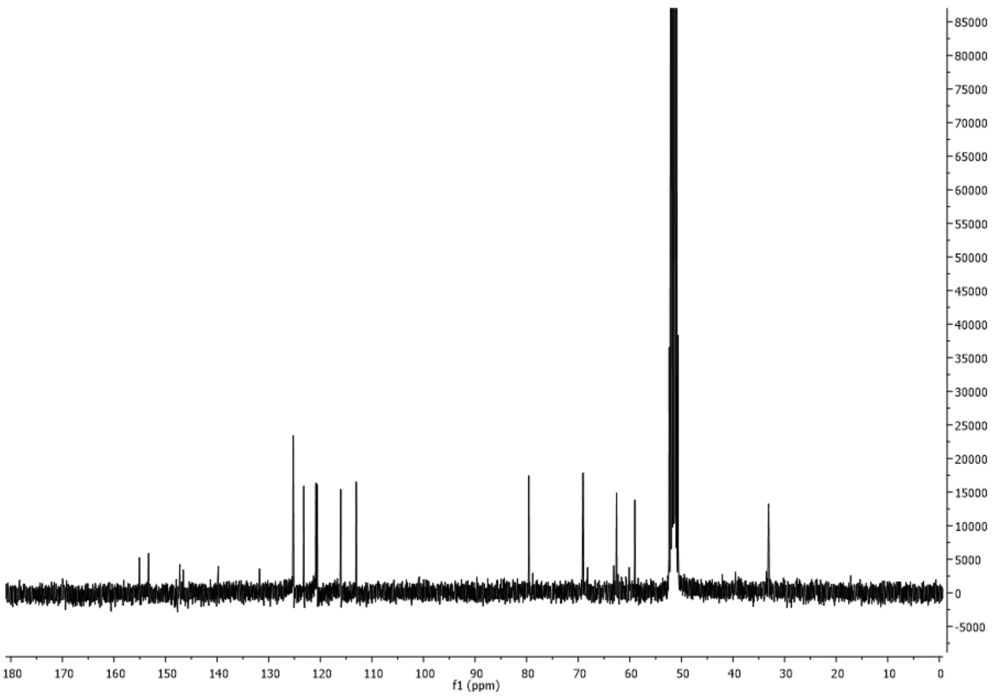

Figure S25. ^{1}H NMR spectrum of compound **11**

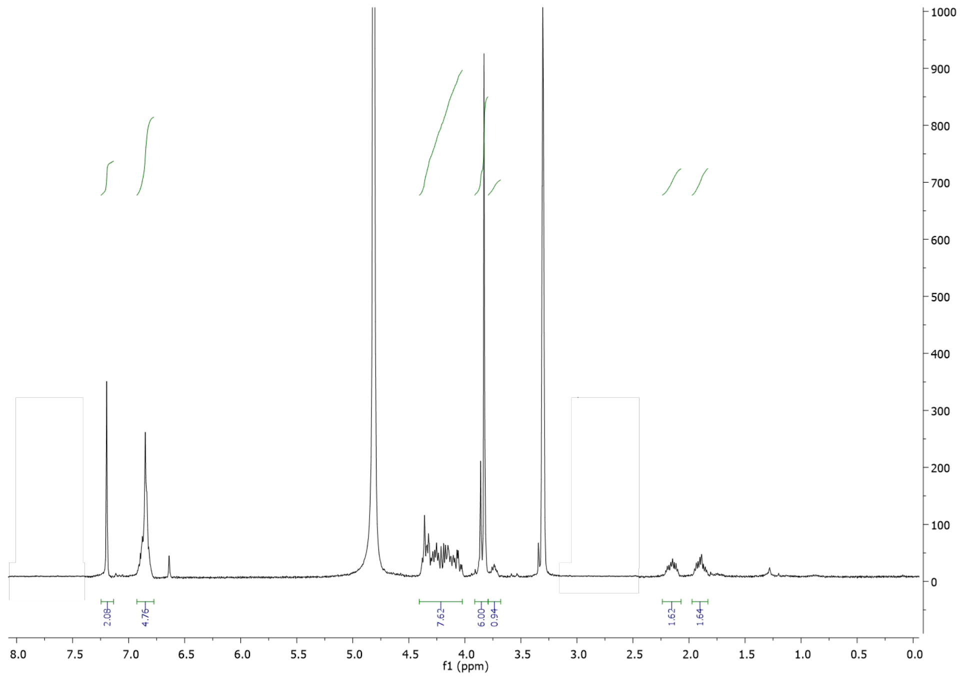

Figure S26. ^{13}C NMR spectrum of compound **11**

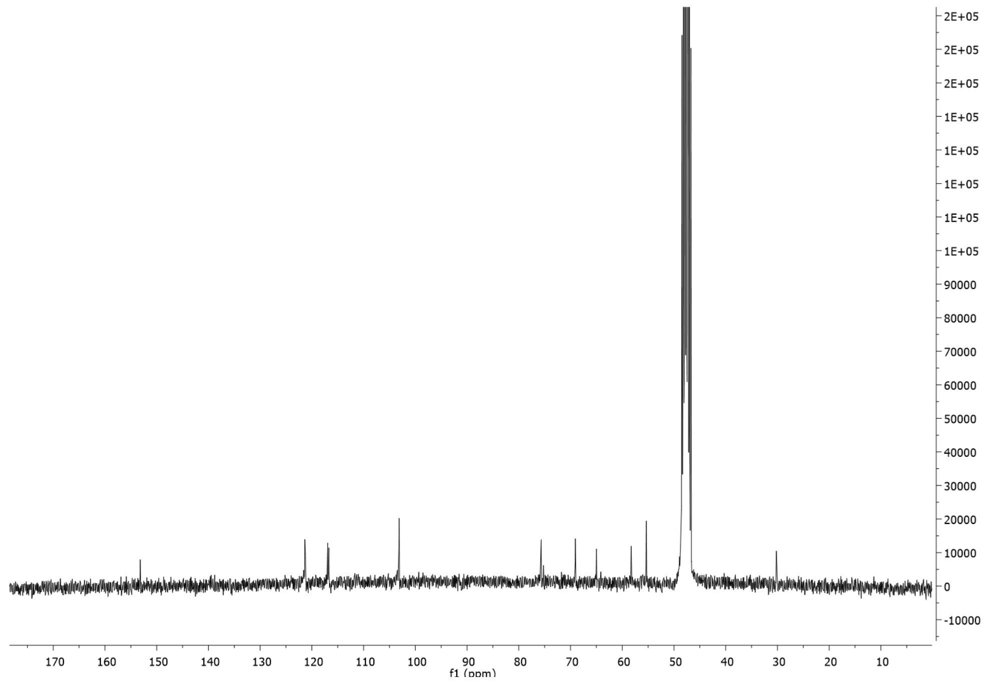

Figure S27. ^{1}H NMR spectrum of compound **12**

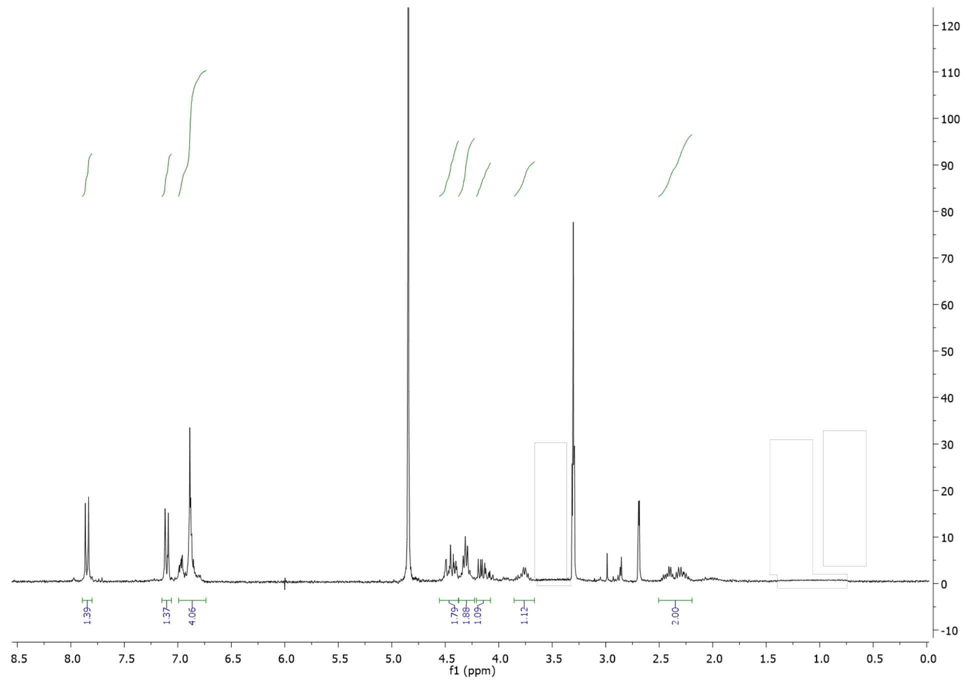

Figure S28. ^{13}C NMR spectrum of compound **12**

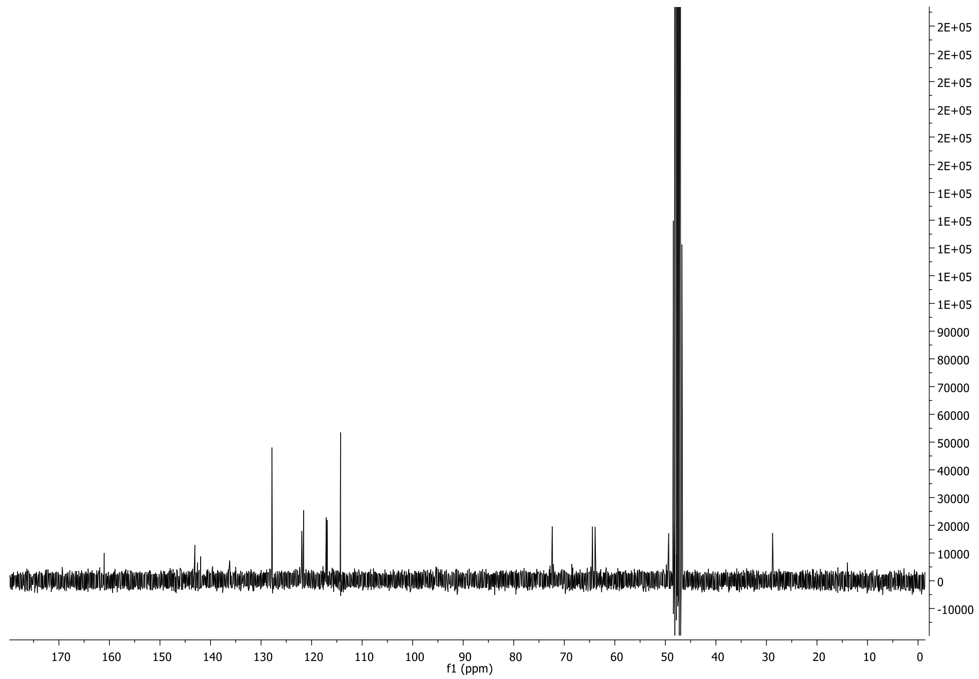

Figure S29. ^{1}H NMR spectrum of compound **13**

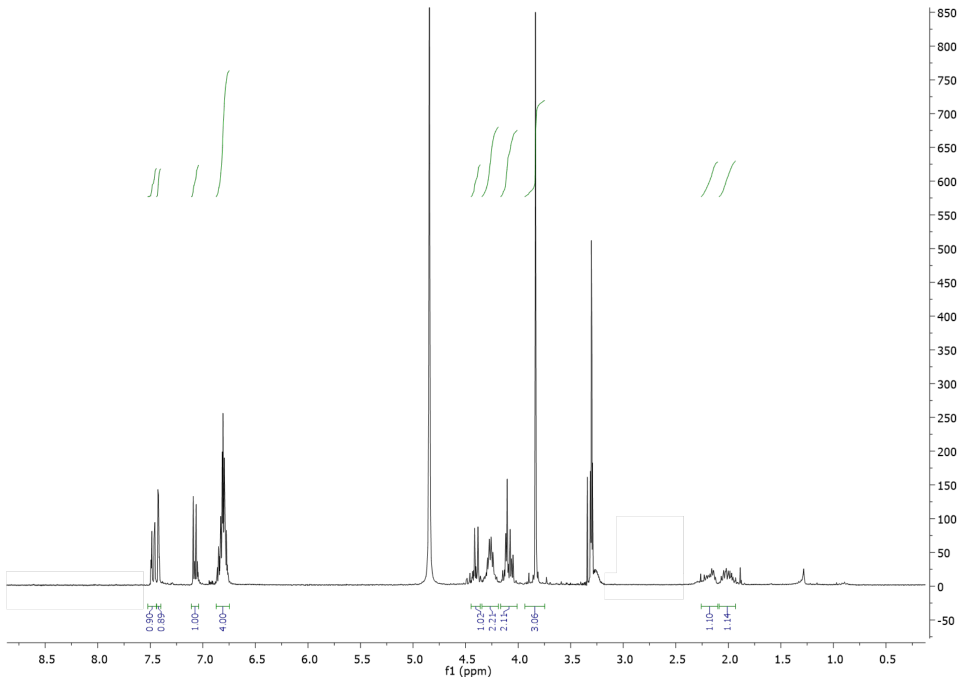

Figure S30. ^{13}C NMR spectrum of compound **13**

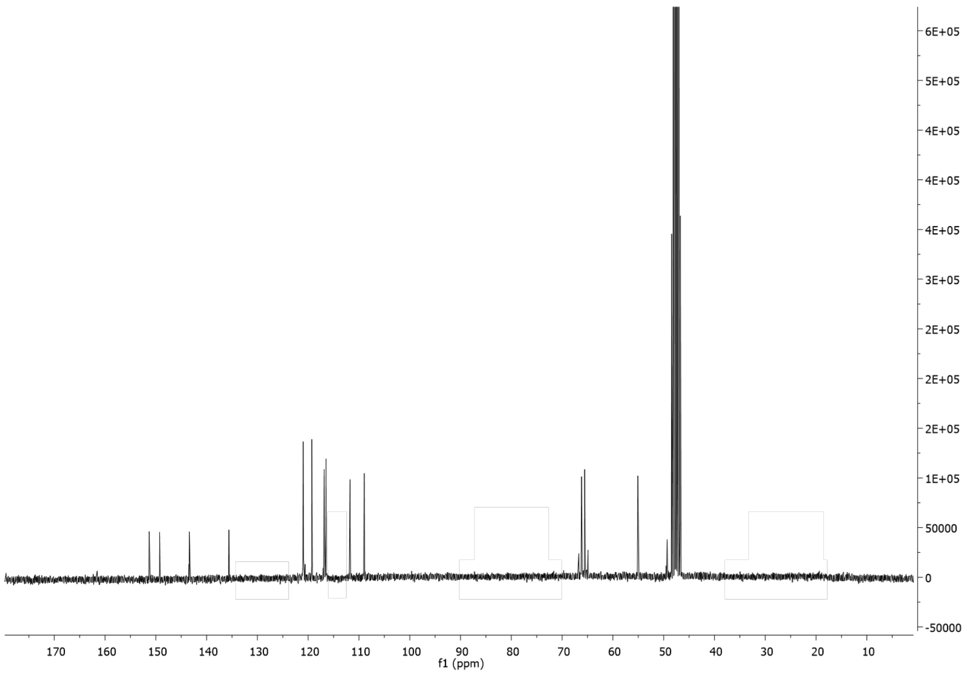

Figure S31. ^{1}H NMR spectrum of compound **14**

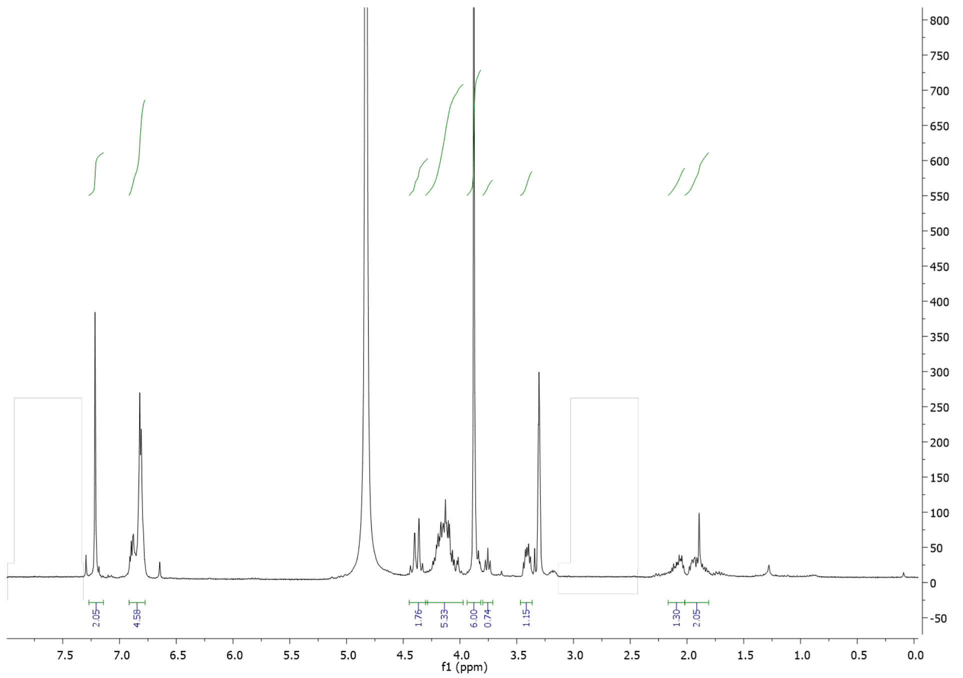

Figure S32. ^{13}C NMR spectrum of compound **14**

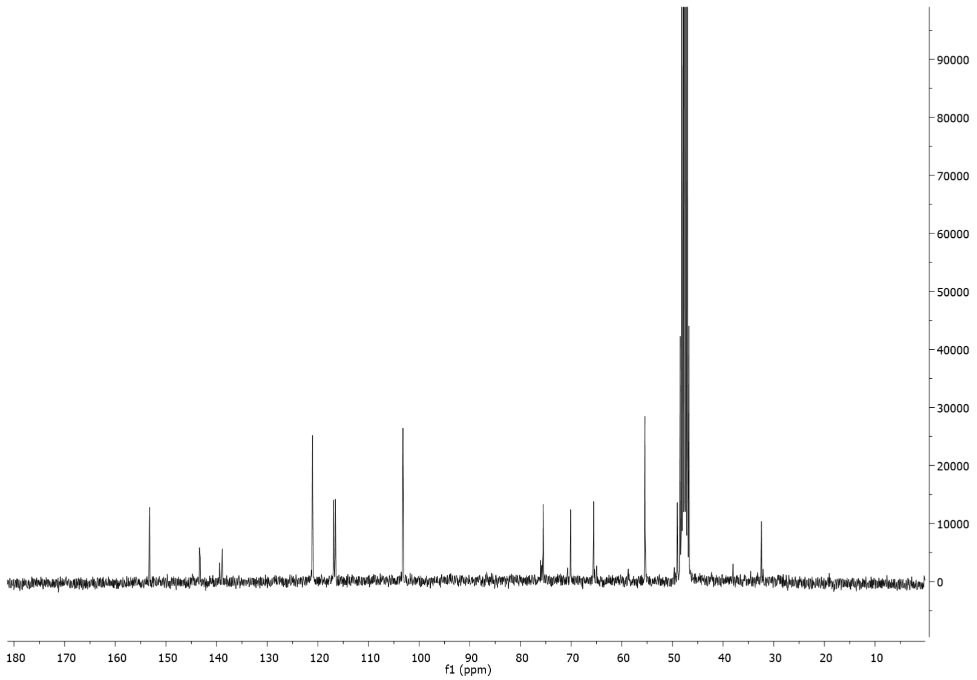

MASS AND HPLC SPECTRA OF FINAL COMPOUNDS 1-14

High-resolution mass spectra were acquired with an LTQ Orbitrap XL mass spectrometer (Thermo Scientific, Milan, Italy) equipped with an ESI source. Source parameters for the positive ion mode were set as follows: spray voltage 4.0 kV, capillary temperature 275 °C, sheath gas 10 a.u., capillary voltage 42 V, tube lens offset 90 V; for the negative ion mode: spray voltage -3.5 kV, capillary temperature 275 °C, sheath gas 10 a.u., capillary voltage -33 V, tube lens offset 180 V. Full MS spectra were acquired in profile mode by the FT analyzer in a scan range of m/z 120–800, using a resolution of 30,000 FWHM at *m/z* 400. The software Xcalibur 4.0 was used for instrument control and spectra analysis. Each compound was dissolved in methanol to obtain a stock solution at a concentration of 1 mg/mL and then further diluted 1:25 with $CH_3CN/H_2O/HCOOH$ 50:50:0.1 (% v/v) before the direct infusion into the mass spectrometer.

HPLC analysis were performed on VARIAN Pro Star running Galaxie software using a Gemini C18 250 x 4.60 mm 5 μm, (Phenomenex, USA) column.; DAD, at a wavelength of 254 nm

Method **A**
Gradient elution conditions: 0.1% TFA in H_2O - ACN; 30% ACN.
Flow rate: 0.6 mL/min

Method **B**
Gradient elution conditions: H_2O - ACN; 0-5', 60% ACN; 5-15', 60-70% ACN; 15-25', 70% ACN.
Flow rate : 0.6 mL/min

Figure S33. A) high-resolution mass spectrum of compound **1** characterized by the $[M+H]^+$ ion at m/z 365.1165 ($[M+H]^+$ calc. 365.1166, Δ ppm 0.137) and B) its isotopic pattern.

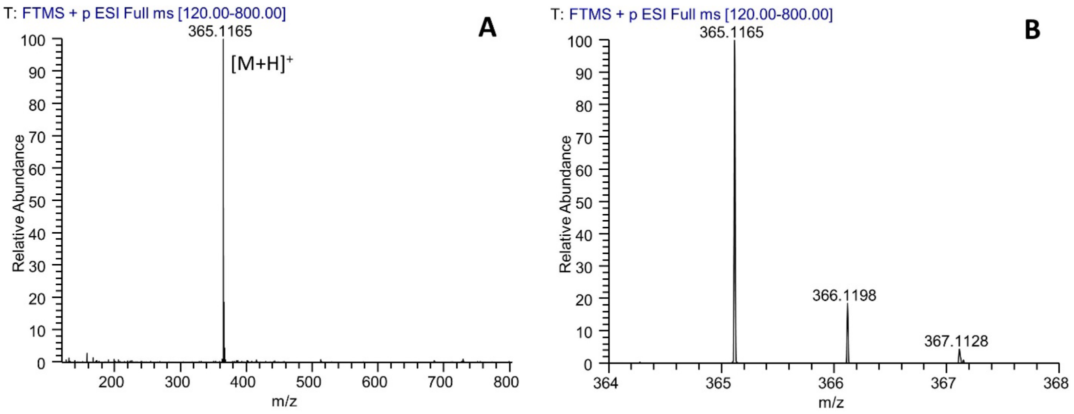

Figure S34. HPLC chromatogram of compound **1** - Method **A**

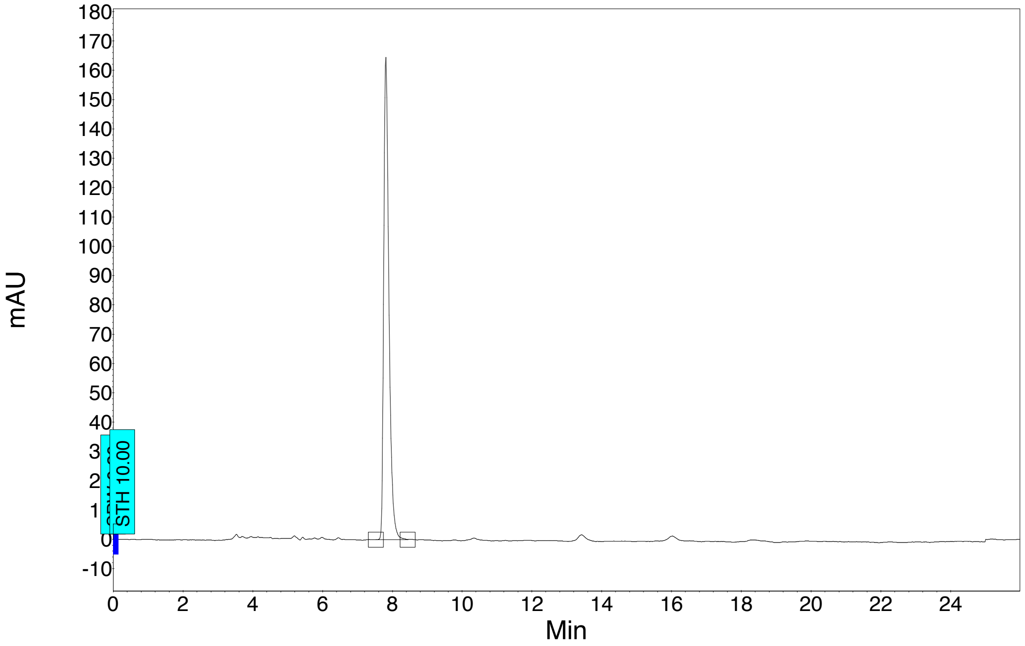

	Name	Area %	Area	Time
		[%]	[mAU.Min]	[Min]
	UNKNOWN	100.000	28.3	7.81
Total		100.000	28.3	

Figure S35. A) high-resolution mass spectrum of compound **2** characterized by the $[M+H]^+$ ion at m/z 379.1318 ($[M+H]^+$ calc. 379.1321, Δ ppm 0.923) and B) its isotopic pattern.

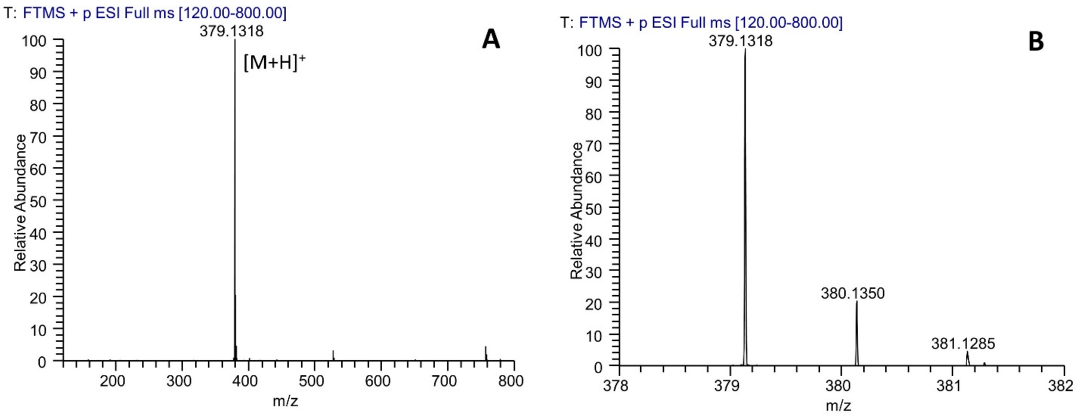

Figure S36. HPLC chromatogram of compound **2** - Method **A**

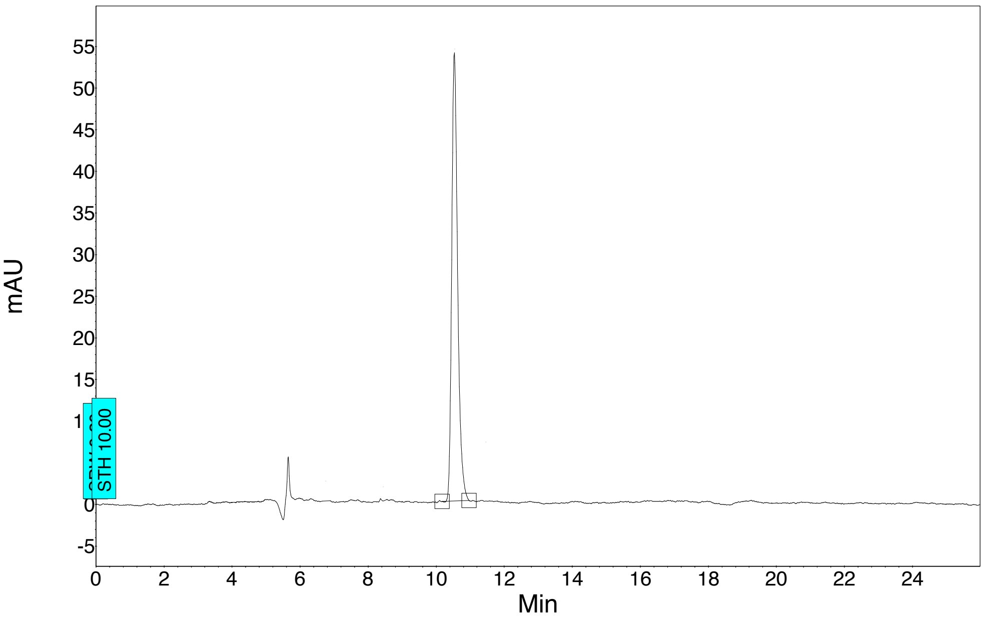

	Name	Area %	Area	Time
		[%]	[mAU.Min]	[Min]
	UNKNOWN	100.000	10.4	10.53
Total		100.000	10.4	

Figure S37. A) high-resolution mass spectrum of compound **3** characterized by the $[M+H]^+$ ion at m/z 393.1473 ($[M+H]^+$ calc. 393.1479, Δ ppm 1.399) and the $[2M+H]^+$ ion at m/z 785.2882, and B) the $[M+H]^+$ isotopic pattern.

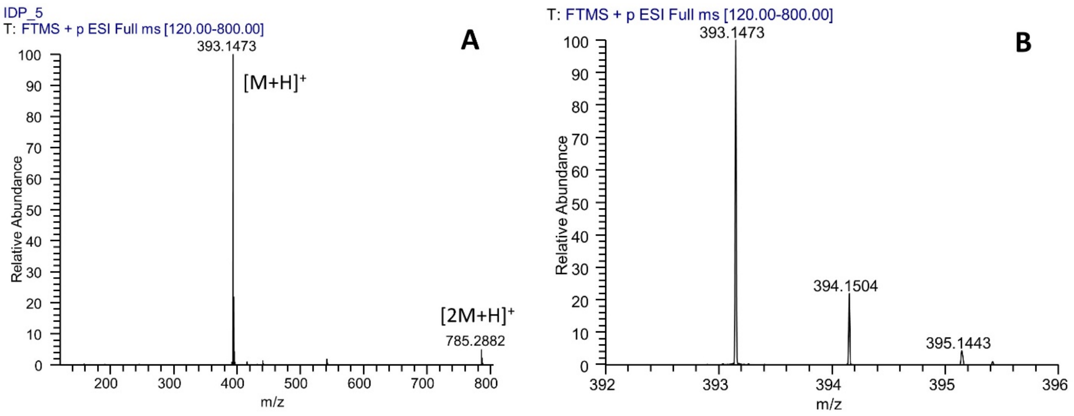

Figure S38. HPLC chromatogram of compound **3**

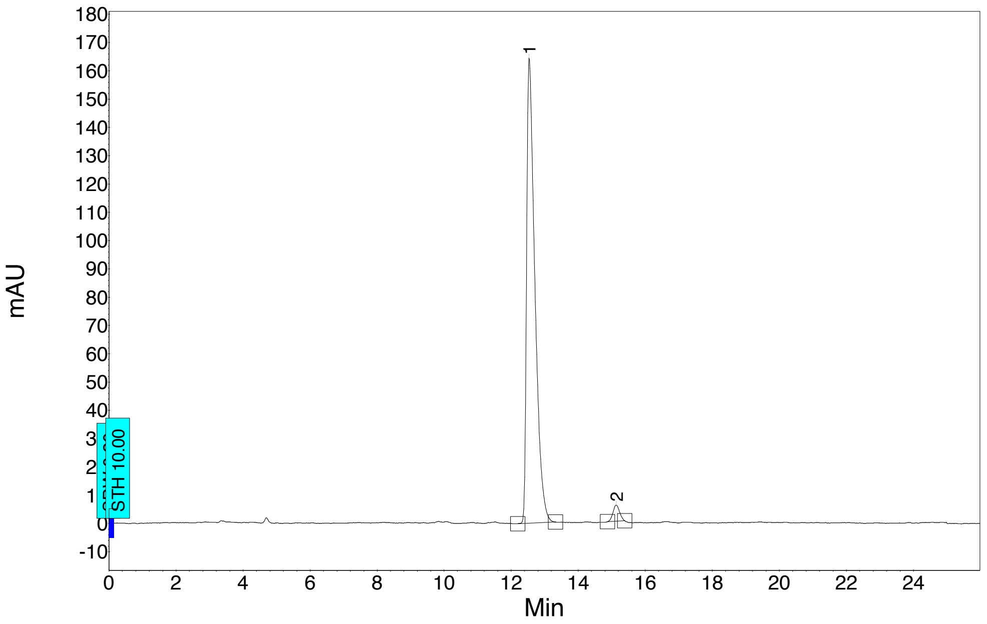

	Name	Area %	Area	Time
		[%]	[mAU.Min]	[Min]
	1	97.319	47.2	12.53
	2	2.681	1.3	15.13
Total		100.000	48.5	

Figure S39. A) high-resolution mass spectrum of compound **4** characterized by the [M+H]$^+$ ion at m/z 393.1475 ([M+H]$^+$ calc. 393.1479, Δ ppm 0.890) and the [2M+H]$^+$ ion at m/z 785.2881, and B) the [M+H]$^+$ isotopic pattern.

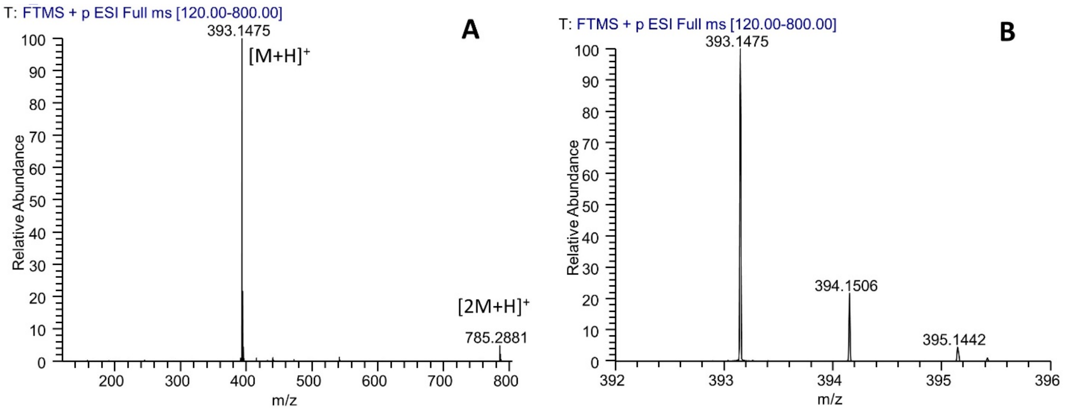

Figure S40. HPLC chromatogram of compound **4** - Method **A**

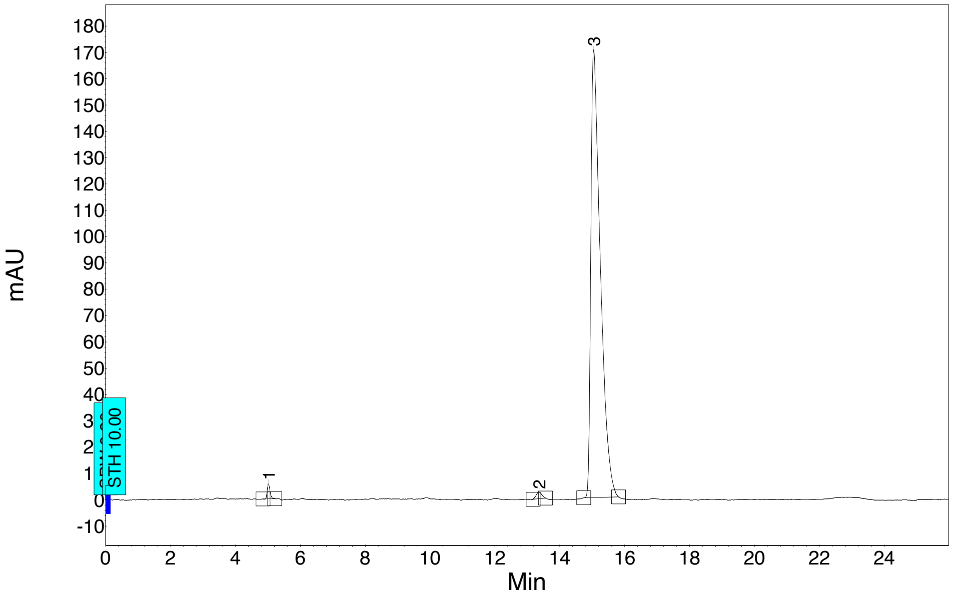

	Name	Area %	Area	Time
		[%]	[mAU.Min]	[Min]
	1	0.940	0.6	5.01
	2	0.815	0.5	13.36
	3	98.245	57.8	15.04
Total		100.000	58.9	

Figure S41. A) high-resolution mass spectrum of compound **5** characterized by the [M+H]+ ion at m/z 407.1639 ([M+H]+ calc. 407.1636, Δ ppm -0.860) and B) its isotopic pattern.

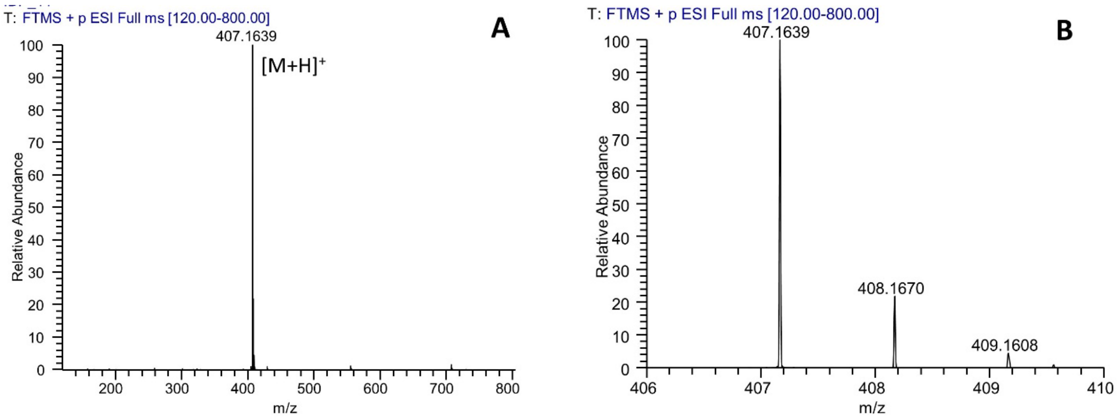

Figure S42. HPLC chromatogram of compound **5** - Method **A**

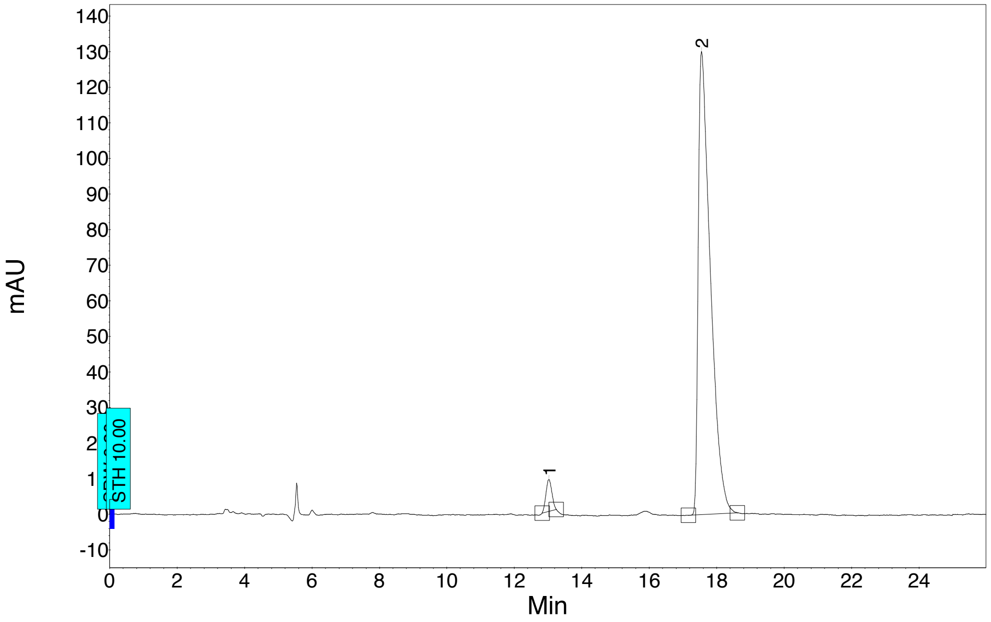

	Name	Area %	Area	Time
		[%]	[mAU.Min]	[Min]
	1	3.209	1.8	13.03
	2	96.791	54.1	17.55
Total		100.000	55.9	

Figure S43. A) high-resolution mass spectrum of compound **6** characterized by the $[M+H]^+$ ion at m/z 379.1319 ($[M+H]^+$ calc. 379.1321, Δ ppm 0.659) and B) its isotopic pattern.

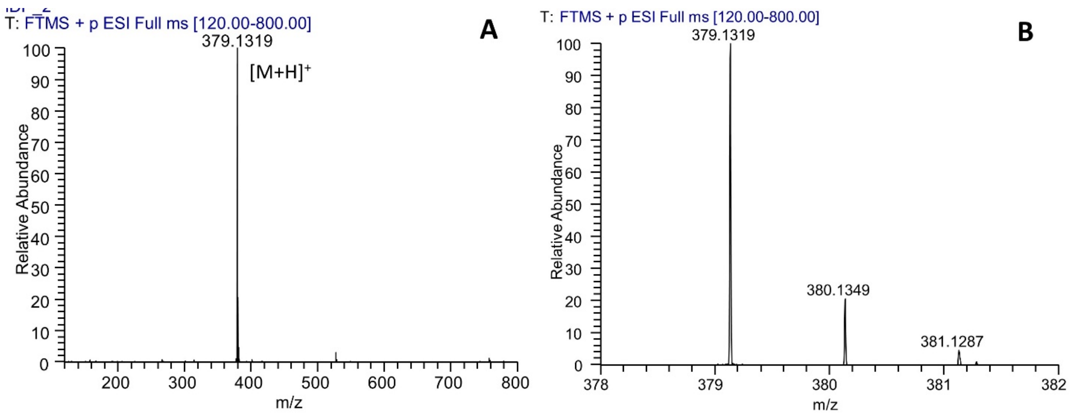

Figure S44. HPLC chromatogram of compound **6** - Method **A**

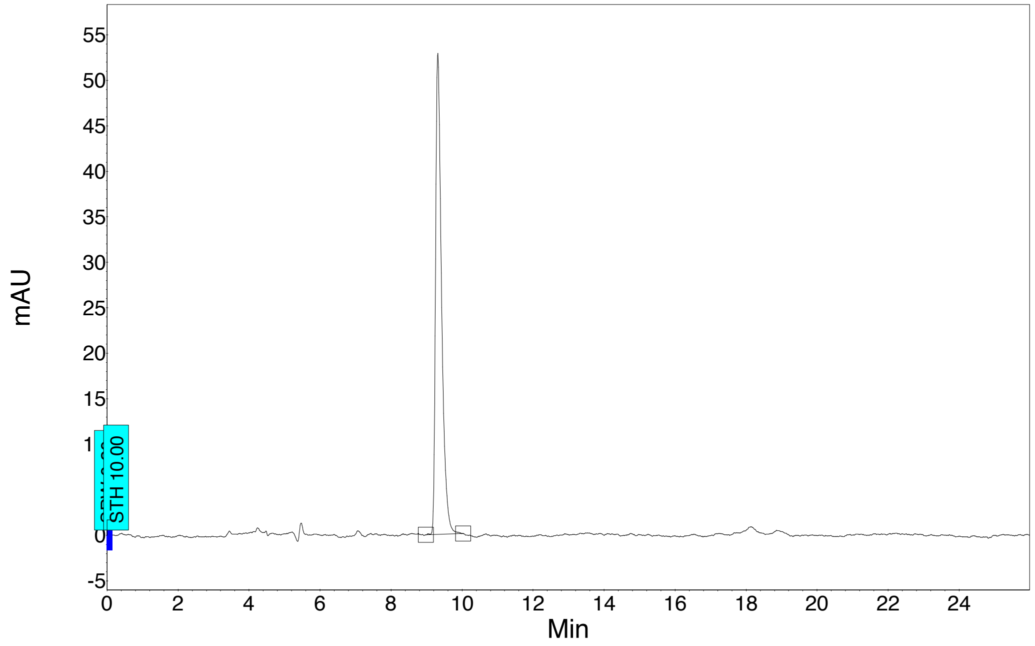

	Name	Area %	Area	Time
		[%]	[mAU.Min]	[Min]
	UNKNOWN	100.000	10.4	9.32
Total		100.000	10.4	

Figure S45. A) high-resolution mass spectrum of compound **7** characterized by the $[M+H]^+$ ion at m/z 393.1475 ($[M+H]^+$ calc. 393.1479, Δ ppm 0.890) and B) its isotopic pattern.

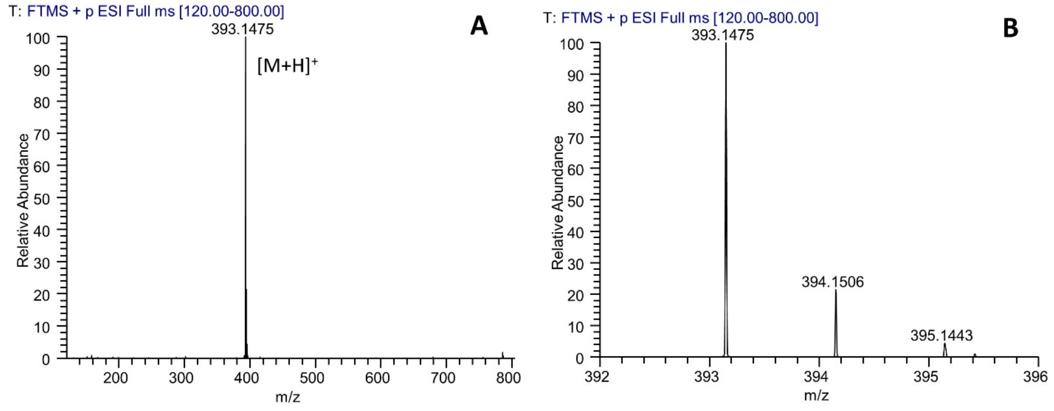

Figure S46. HPLC chromatogram of compound **7** - Method **A**

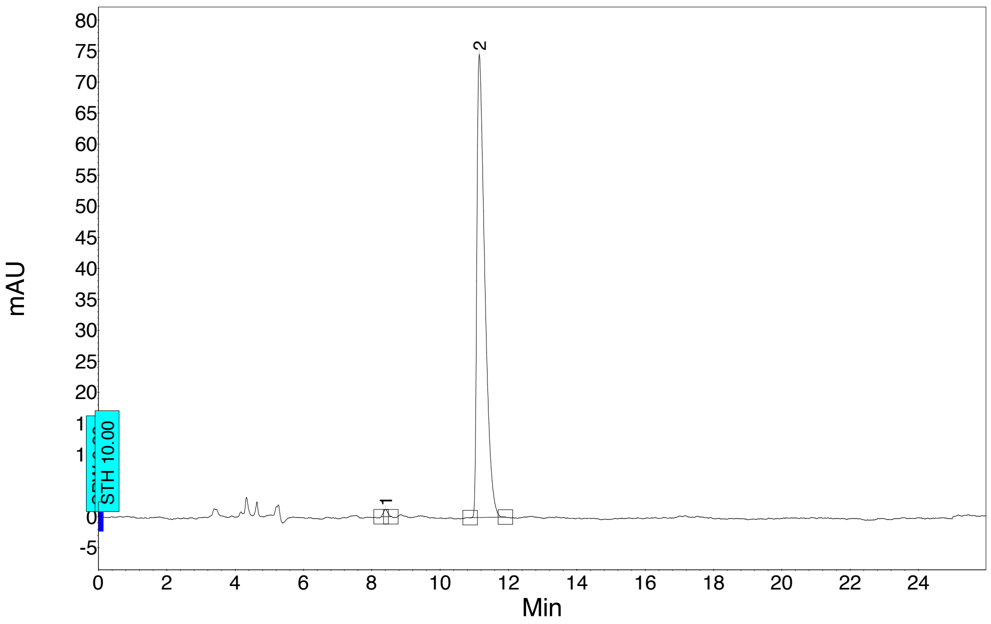

	Name	Area %	Area	Time
		[%]	[mAU.Min]	[Min]
	1	0.853	0.2	8.40
	2	99.147	20.1	11.15
Total		100.000	20.2	

Figure S47. A) high-resolution mass spectrum of compound **8** characterized by the [M+H]$^+$ ion at m/z 421.1785 ([M+H]$^+$ calc. 421.1792, Δ ppm 1.543) and B) its isotopic pattern.

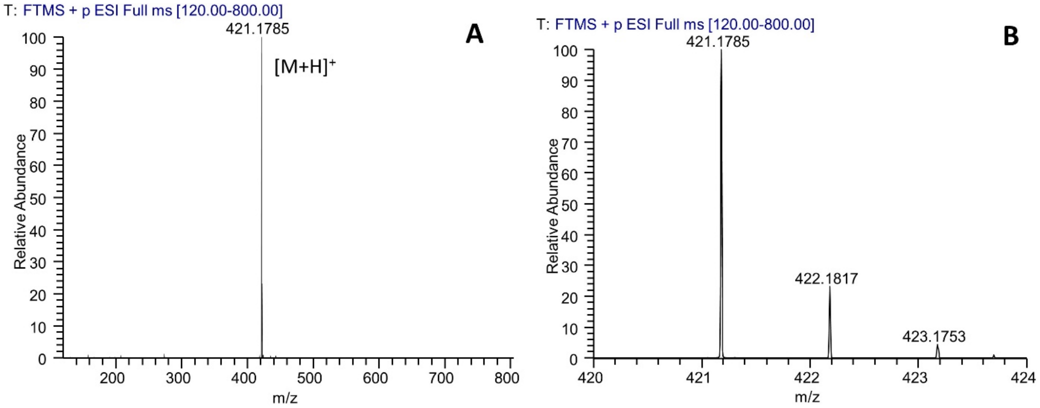

Figure S48. HPLC chromatogram of compound **8** - Method **A**

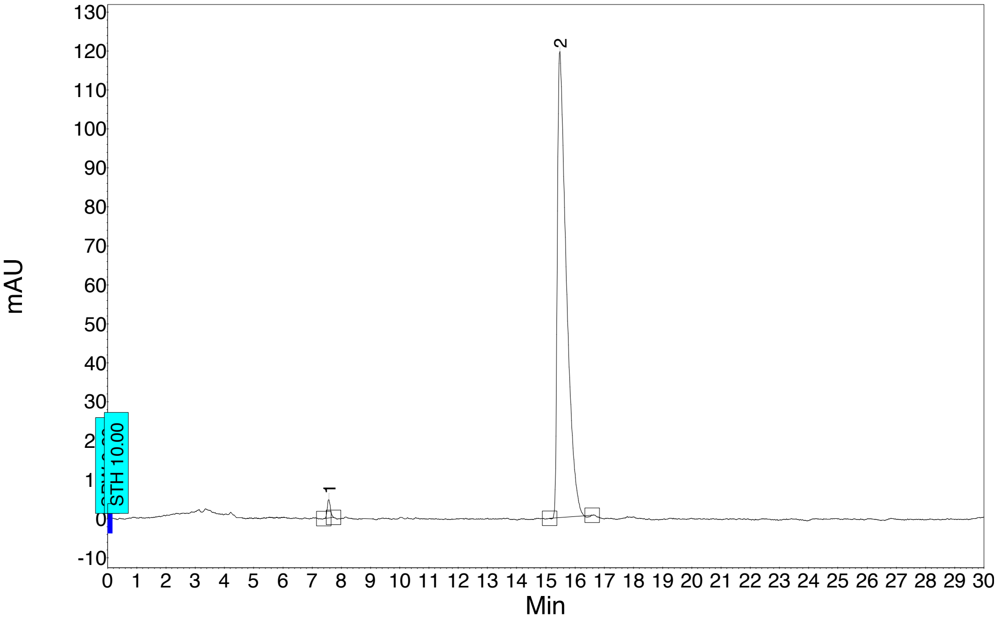

	Name	Area %	Area	Time
		[%]	[mAU.Min]	[Min]
	1	1.335	0.6	7.57
	2	98.665	42.8	15.48
Total		100.000	43.4	

Figure S49. A) high-resolution mass spectrum of compound **9** characterized by the [M-H]- ion at m/z 389.0923 ([M-H]- calc. 389.0926, Δ ppm 0.643) and B) its isotopic pattern.

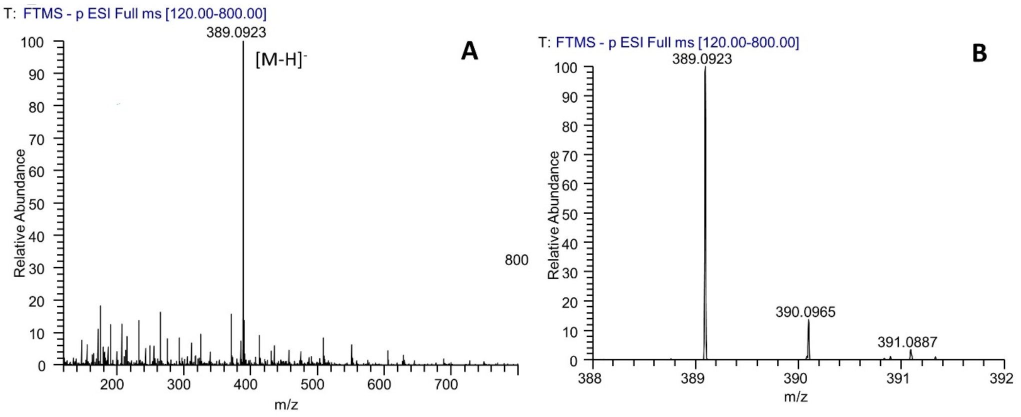

Figure S50. HPLC chromatogram of compound **9** - Method **A**

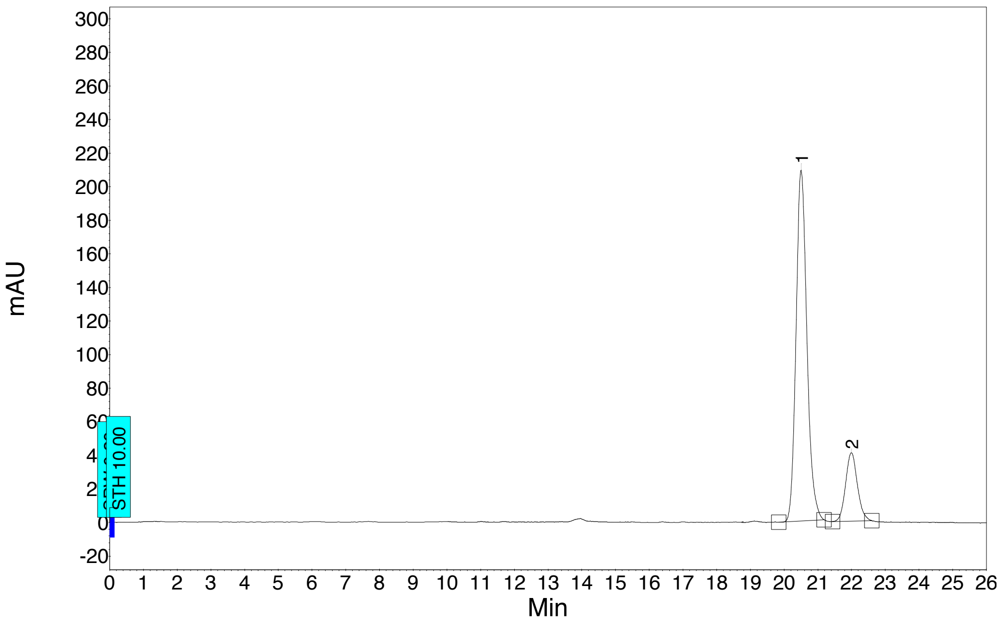

	Name	Area %	Area	Time
		[%]	[mAU.Min]	[Min]
	1	82.556	77.9	20.51
	2	17.444	16.5	22.00
Total		100.000	94.3	

Figure S51. A) high-resolution mass spectrum of compound **10** characterized by the [M-H]⁻ ion at m/z 419.1027 ([M-H]⁻ calc. 419.1031, Δ ppm 0.835) and B) its isotopic pattern.

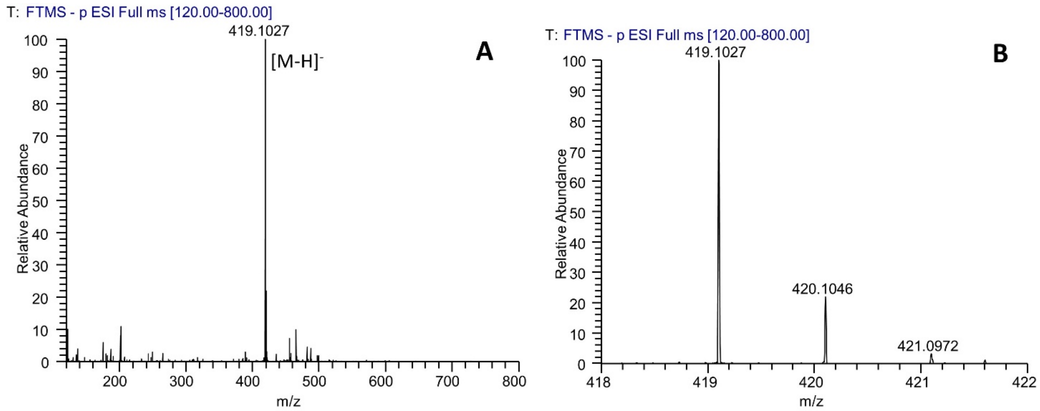

Figure S52. HPLC chromatogram of compound **10** - Method **B**

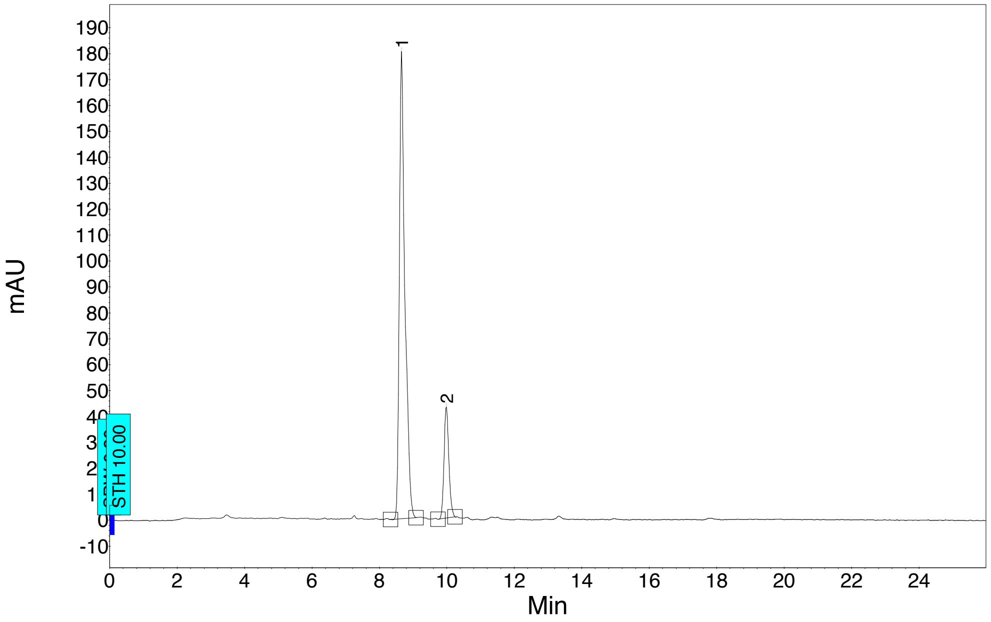

	Name	Area %	Area	Time
		[%]	[mAU.Min]	[Min]
	1	83.097	34.9	8.65
	2	16.903	7.1	9.99
Total		100.000	41.9	

Figure S53. A) high-resolution mass spectrum of compound **11** characterized by the [M-H]- ion at m/z 449.1142 ([M-H]- calc. 449.1137, Δ ppm -1.225) and B) its isotopic pattern.

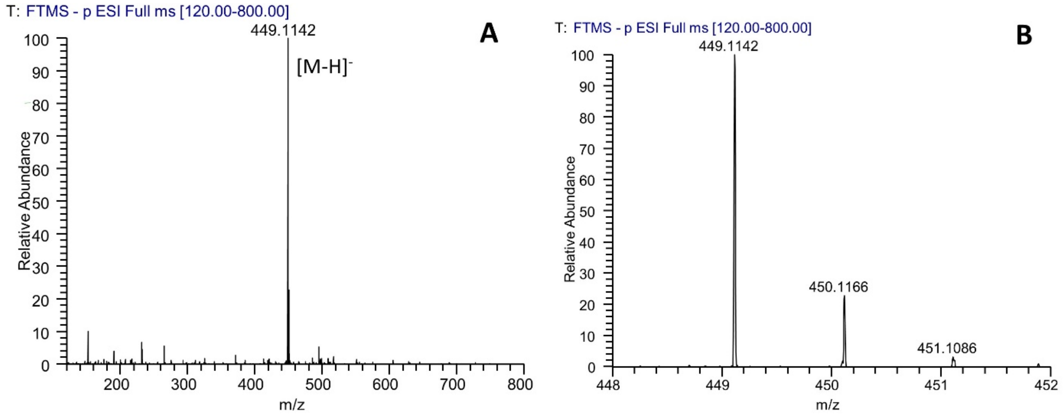

Figure S54. HPLC chromatogram of compound **11** - Method **B**

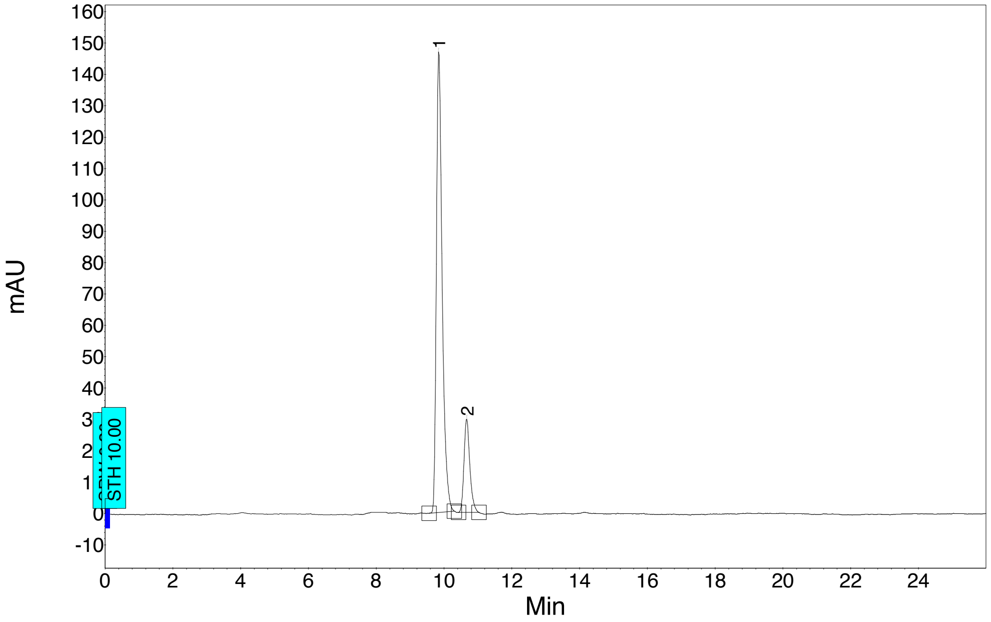

	Name	Area %	Area	RT
		[%]	[mAU.Min]	[Min]
	1	81.515	29.5	9.84
	2	18.485	6.7	10.67
Total		100.000	36.2	

Figure S55. A) high-resolution mass spectrum of compound **12** characterized by the $[M+H]^+$ ion at m/z 365.1166 ($[M+H]^+$ calc. 365.1166, Δ ppm -0.137) and B) its isotopic pattern.

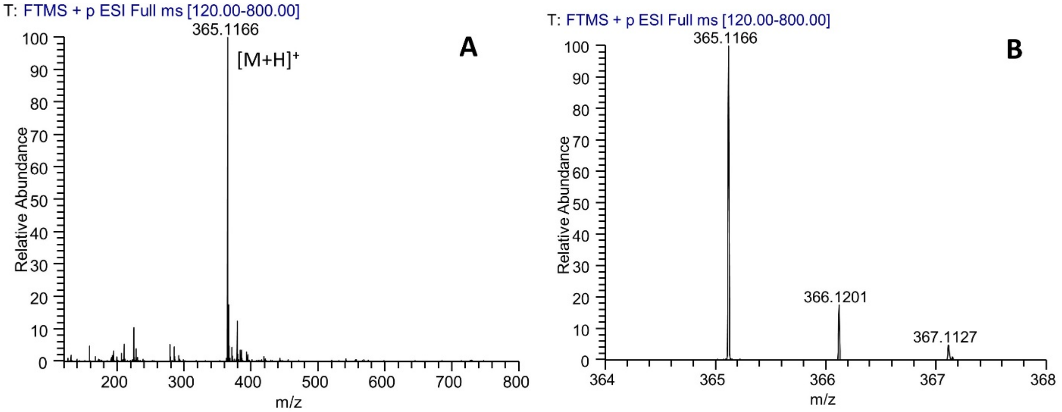

Figure S56. HPLC chromatogram of compound **12** - Method **A**

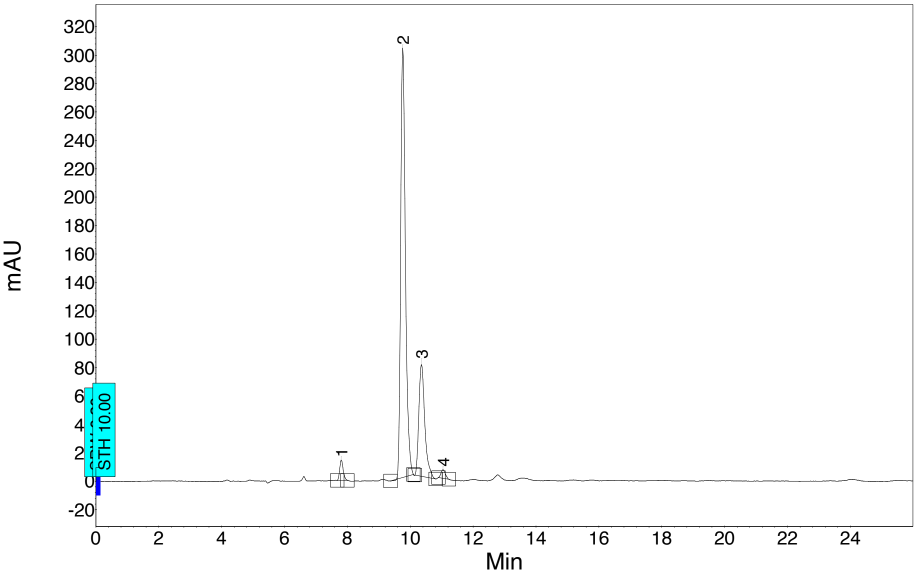

	Name	Area %	Area	Time
		[%]	[mAU.Min]	[Min]
	1	2.484	1.8	7.80
	2	72.628	52.8	9.76
	3	23.551	17.1	10.36
	4	1.336	1.0	11.04
Total		100.000	72.7	

Figure S57. A) high-resolution mass spectrum of IDP 17 characterized by the [M+H]$^+$ ion at m/z 365.1166 ([M+H]$^+$ calc. 365.1166, Δ ppm -0.137) and B) its isotopic pattern.

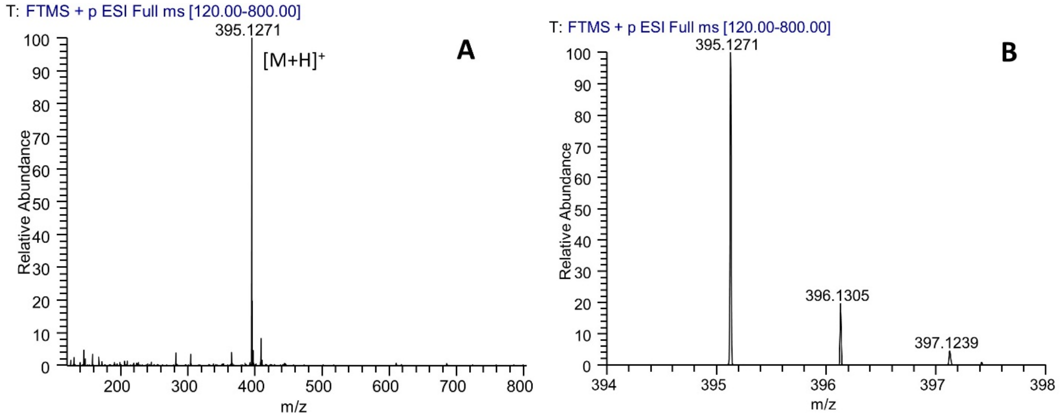

Figure S58. HPLC chromatogram of compound **13** - Method **A**

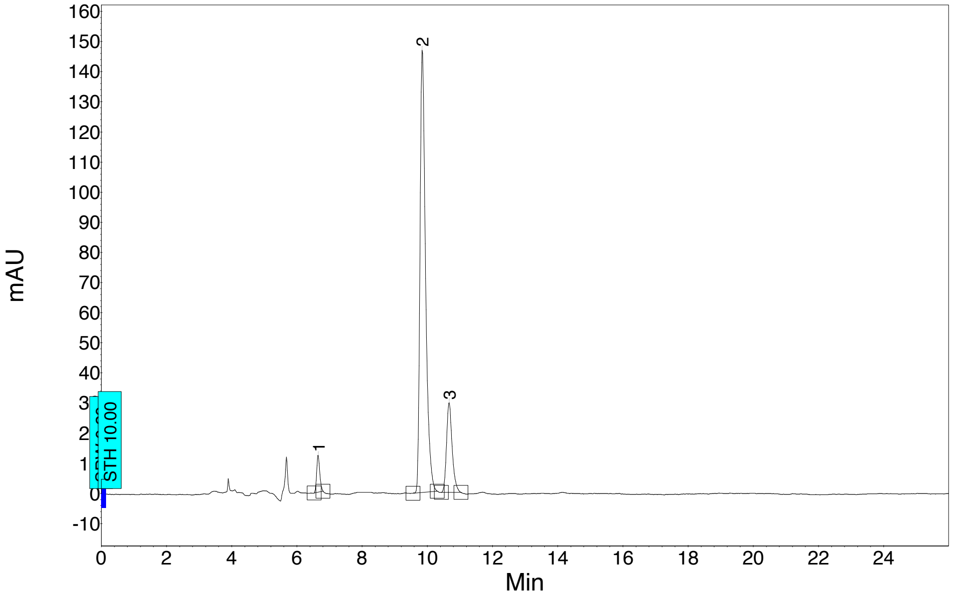

	Name	Area %	Area	RT
		[%]	[mAU.Min]	[Min]
	1	3.798	1.4	6.65
	2	79.616	28.8	9.84
	3	16.586	6.0	10.67
Total		100.000	36.2	

Figure S59. A) high-resolution mass spectrum of compound **14** characterized by the $[M+H]^+$ ion at m/z 425.1371 ($[M+H]^+$ calc. 425.1377, Δ ppm 1.294) and B) its isotopic pattern.

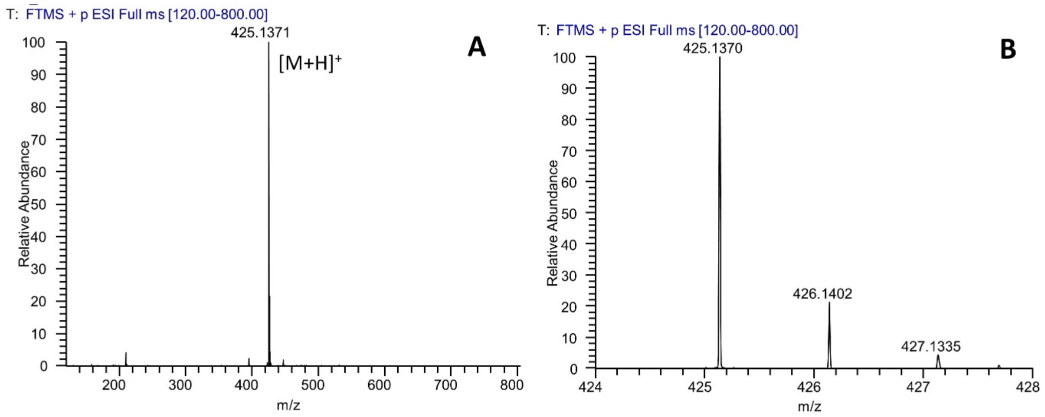

Figure S60. HPLC chromatogram of compound **14** - Method **A**

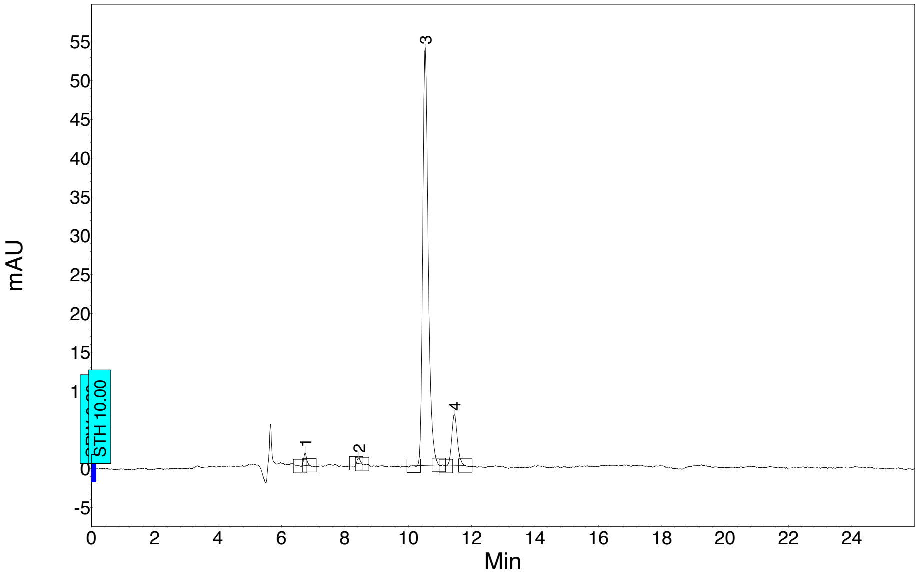

	Name	Area %	Area	Time
		[%]	[mAU.Min]	[Min]
	1	1.407	0.2	6.75
	2	0.580	0.1	8.44
	3	86.972	10.8	10.53
	4	11.040	1.4	11.45
Total		100.000	12.5	

REFERENCES

[1] Pallavicini M., Budriesi R., Fumagalli L., Ioan P., Chiarini A., Bolchi C., Ugenti M. P., Colleoni S., Gobbi M., Valoti E. "WB4101-Related Compounds: New, Subtype-Selective α1-Adrenoreceptor Antagonists (or Inverse Agonists?)" *Journal of Medicinal Chemistry,* **2006***,* 49,24, 7140-9. https://doi.org/10.1021/jm060358r.

[2] Del Bello, F.; Bonifazi, A.; Giannella, M.; Giorgioni, G.; Piergentili, A.; Petrelli, R.; Cifani, C.; Micioni Di Bonaventura, M. V.; Keck, T. M.; Mazzolari, A.; Vistoli, G.; Cilia, A.; Poggesi, E.; Matucci, R.; Quaglia, W. "The replacement of the 2-methoxy substituent of N-((6, 6-diphenyl-1, 4-dioxan-2-yl) methyl)-2-(2-methoxyphenoxy) ethan-1-amine improves the selectivity for 5-HT1A receptor over α1-adrenoceptor and D2-like receptor subtypes." *European Journal of Medicinal Chemistry,* **2017**, 125, 233– 244. https://doi.org/10.1016/j.ejmech.2016.09.026.
